# *ScreenMill*: A freely available software suite for growth measurement, analysis and visualization of high-throughput screen data

**DOI:** 10.1186/1471-2105-11-353

**Published:** 2010-06-28

**Authors:** John C Dittmar, Robert JD Reid, Rodney Rothstein

**Affiliations:** 1 Columbia University, Dept. of Biological Sciences, New York, NY 10027, USA; 2 Columbia University Medical Center, Dept. Genetics & Development, 701 West 168th Street, New York, NY 10032-2704, USA

## Abstract

**Background:**

Many high-throughput genomic experiments, such as Synthetic Genetic Array and yeast two-hybrid, use colony growth on solid media as a screen metric. These experiments routinely generate over 100,000 data points, making data analysis a time consuming and painstaking process. Here we describe *ScreenMill*, a new software suite that automates image analysis and simplifies data review and analysis for high-throughput biological experiments.

**Results:**

The *ScreenMill*, software suite includes three software tools or "engines": an open source *Colony Measurement Engine *(*CM Engine*) to quantitate colony growth data from plate images, a web-based *Data Review Engine *(*DR Engine*) to validate and analyze quantitative screen data, and a web-based *Statistics Visualization Engine *(*SV Engine*) to visualize screen data with statistical information overlaid. The methods and software described here can be applied to any screen in which growth is measured by colony size. In addition, the *DR Engine *and *SV Engine *can be used to visualize and analyze other types of quantitative high-throughput data.

**Conclusions:**

*ScreenMill *automates quantification, analysis and visualization of high-throughput screen data. The algorithms implemented in S*creenMill *are transparent allowing users to be confident about the results *ScreenMill *produces. Taken together, the tools of *ScreenMill *offer biologists a simple and flexible way of analyzing their data, without requiring programming skills.

## Background

Based on genome sequence information, comprehensive clone and gene deletion libraries have been created where each gene is individually expressed or deleted. Genetic techniques have been developed to exploit these resources, which has led to an explosion in the number of high-throughput biological experiments for many organisms. Advances in automation technology are also increasing the efficiency and driving down the costs of performing these experiments. The budding yeast *Saccharomyces cerevisiae *has provided a robust platform for many high-throughput experiments, examples include: yeast-two hybrid screens to discover novel protein-protein interactions [1-3], chemical genetic screens to determine the target of a particular inhibitory compound [4], and synthetic lethal (SL) and synthetic dosage lethal (SDL) screens to discover novel genetic interactions [5-12]. The readout from these screens is typically growth of yeast colonies arranged in a grid on solid media and comparisons are made between experimental and control conditions to evaluate a biological effect.

Visual inspection has been used effectively to evaluate high-throughput growth data, but the task is time consuming and subjective [8]. Raw data may need to be reviewed several times to ensure accuracy, a feat made difficult by the fact that high-throughput screens often produce 100,000 or more data points, with significant growth differences, or "hits," representing only a small fraction of the total. In addition, without quantitative data the results are typically binary (growth vs. no growth) and therefore, biologically relevant information may be lost.

Quantitative data can help identify more biologically relevant information from a screen such as weak vs. strong interactions or even suppressors [13]. Imaging tools such as *ImageJ *[14], *CellProfiler *[15], or *HT Colony Grid Analyzer *[16] can be used to quantify colony growth, but these tools can only provide raw colony size data, with no statistical analysis of the data they produce. In addition, *ImageJ *and *CellProfiler *require knowledge of image manipulation and programming to adapt them for use with a particular experiment. Statistical analysis can be achieved with programs such as *Growth Detector *[17], *Colony Imager *[18] and *Colony Scorer *[18]. However, none of these programs have all of the features expected in a fully automated system. For instance, the analysis methods used in *Colony Imager *and *Colony Scorer *are not completely described. A transparent system would allow more evaluation, and perhaps, customization of statistical methods. In contrast, *Growth Detector *provides source code, so the methods are transparent, however the code must be edited prior to use and requires the proprietary software, *MATLAB*, to run. Finally, none of these programs provide visualization tools to help evaluate numerical output.

Here we present a new software suite called *ScreenMill *that overcomes the limitations described above. The methods employed by *ScreenMill *are transparent and require no proprietary software or sophisticated programming knowledge for their use. *ScreenMill *allows users to obtain quantitative data from high-throughput growth experiments, streamlines the statistical analysis, and offers a novel web-based application to review and visualize data.

## Implementation

**Figure 1:**
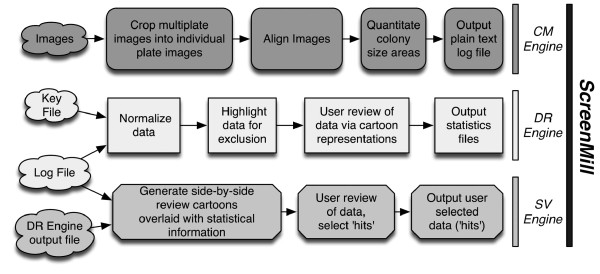
***ScreenMill *workflow**. Clouds represent inputs for each of the programs. Processing steps in *CM*, *DR*, and *SV Engines *are represented as dark gray, white or light gray boxes, respectively. See **Additional File **1 for details on how to properly format input files.

The *ScreenMill *software suite is composed of three software tools: Colony Measurement Engine (*CM Engine*), Data Review Engine (*DR Engine*), and Statistics Visualization Engine (*SV Engine*) (**Figure **1). The *CM Engine *automates the processing of digital plate images, computes colony areas, and saves the raw growth data to a file. This file is uploaded to the *DR Engine*, which provides a graphic user interface (GUI) with a visual representation of the raw data. The GUI provides tools for visualizing data and identifying anomalies. The *DR Engine *then calculates population statistics for a dataset and saves the results in a text file. The *SV Engine *accepts the results generated by the *CM *and *DR *engines as inputs and uses a GUI similar to the *DR Engine *to provide a unique visualization of the statistics for side-by-side comparison of control and experimental plates from a screen. This tool helps to review and refine the list of significant interactions identified in a screen.

### Colony Measurement Engine (*CM Engine*)

The *CM Engine *has been developed as an open-source macro for the freely available program, *ImageJ *from the National Institutes of Health (NIH) [14]. The function of *CM Engine *is to automatically translate into quantitative data, a directory containing digital images of yeast plates with colonies arranged in a rectangular grid. Prior to using *CM Engine*, images of agar plates are generated using a flatbed scanner or digital camera. Digital images of plates presented in this paper were captured using a ScanMaker 9800XL flatbed scanner (MicroTek International, Inc.). Plates were scanned in 8-bit grayscale at a resolution of 300 dpi using the Transparent Media Adapter. Transparency mode scans eliminate many artifacts caused by reflected light and result in higher contrast images than reflected light scans. A black Plexiglas mask was cut to make a 3 × 3 plate grid to align them on the scanner bed and mask the light between them.

**Figure 2:**
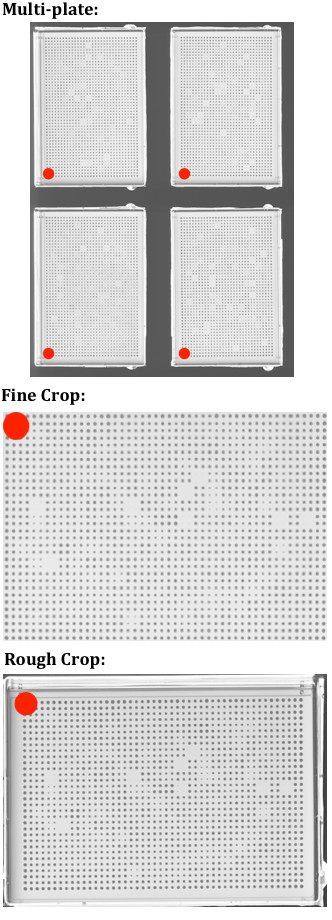
***CM Engine *acceptable image formats**. Red dots in the Figure indicate position A1. Red dots are for illustrative purpose only and should not appear on images processed by *CM Engine*. Images are assumed to be in this orientation by the *CM Engine*. *Note: images are not shown at 100% scale. Full size example images may found in ***Additional File **2* or may be downloaded from the Rothstein Lab website*: http://www.rothsteinlab.com/tools/screen_mill/cm_engine.

*CM Engine *accepts three different image layouts as input: (1) multi-plate images that contain several plates arranged in a defined array, (2) "rough crops", in which images are comprised of individual plates where the borders of the image align roughly with the edge of the plate (and may not reflect precise alignment to the colony grid) and (3) "fine crops", in which the images contain individual plates whose borders align exactly with the edge of the colony grid layout (**Figure **2).

Upon invoking the *CM Engine *macro within *ImageJ*, the user is prompted to select a "parent directory" containing plate images (**Additional File **1 - **Figure S2)**. These images may be in any file format that *ImageJ *can open, including JPEG, GIF, PNG, and TIFF, although images in a lossless format (e.g. PNG and TIFF) at a resolution of at least 300 dpi are recommended. *CM Engine *then allows users to enter several parameters: the mode in which they would like to quantify colony growth, the colony array format used in the screen (384 or 1536 colonies), a label for the file that will store the colony area sizes, and the labels of any conditions (e.g., drug exposure, UV).

**Figure 3:**
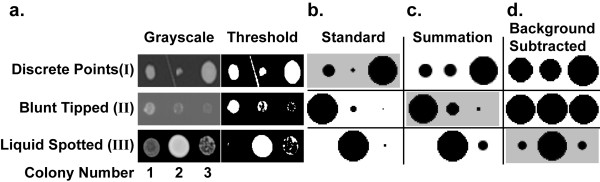
***CM Engine *measurement modes**. (**a**) Images of colonies in grayscale before and after application of a threshold filter. These images represent the three main ways cells are deposited onto agar plates in high-throughput growth experiments (discrete points, blunt tipped and liquid spotted). Three different *CM Engine *measurement modes (Standard, Summation and Background Subtracted) have been developed to handle each of these colony deposition methods. The grayed boxes in **b**, **c **and **d **indicate the preferred measurement mode. Prior to analysis, each image is symmetrically divided into partitions, one for each colony. (**b**) Standard mode is designed for robot pinned colonies that come from a discrete point. Images are subjected to a threshold filter and only the largest particle of each partition is considered. Anomalies are presented to the user and may be removed manually. All colonies in image I are measured accurately since the linear artifact in partition 2 was detected and removed. In image II, only colony 1 is correctly measured since Standard mode chooses the largest particle in each partition, incorrectly measuring the non-contiguous growth in partitions 2 and 3. None of the colonies in image III were measured accurately since the automatic threshold filter used in Standard mode is insensitive to density differences between partitions. (**c**) Summation mode is designed for colonies from blunt tipped pins. Images are subjected to a threshold filter and all particles within each partition are considered part of the colony. In this case, all colonies in image II are measured accurately. Colony 2 of image I was incorrectly measured due to the inclusion of the linear artifact in that partition. This mode also fails for image III since, like Standard mode, it is insensitive to density differences. (**d**) Background Subtracted mode is designed for cells spotted from a liquid culture. The background of the grayscale image is subtracted from each partition and the remaining pixel values are averaged to quantify cell density. In this mode, image III colonies are measured accurately, while image I was not due to the linear artifact in partition 2. Additionally, image II was not measured accurately due to the high background value of the grayscale image. For **b**, **c **and **d**, values are normalized to the largest colony size. Accuracy was determined by manual threshold and measurement as described in the documentation of *ImageJ *software.

Three quantification modes are available (Standard, Summation and Background Subtracted) and are chosen based on how the cells are deposited on the agar plates. For all modes, the fine-cropped plate image is symmetrically partitioned based on the colony array format and growth in each partition is measured. Standard and Summation modes use binary representations of plate images after applying a threshold filter, whereas Background Subtracted mode uses grayscale images (**Figure **3a).

#### *CM Engine *- Standard Measurement Mode

Standard mode is typically used when strains are deposited on plates as discrete points, such as in replica-pin transfers. In these experiments, growth area of a continuous circular colony is measured (**Figure **3b). Standard mode offers robust artifact detection prior to calculating colony areas. In addition, measurements are recorded in Standard mode only after passing a quality control step that is based on a comparison of the number of colonies expected on a plate to the number of colonies detected on a plate.

Similar to *HT Colony Grid Analyzer *[16] and *Growth Detector *[17], the first step of Standard mode applies a binary threshold filter to each image to render plate images as black colonies (particles) with a white background. Every particle on a plate is then analyzed using the *ImageJ *function "Analyze Particles", returning the centroid coordinates, particle size (number of pixels), and circularity (a value between 0 and 1.0, where 0 is the circularity of a straight line and 1.0 is the circularity of a perfect circle). This circularity parameter is used to help identify artifacts that may be present in the image. Any particle whose circularity is below a threshold value (e.g., 0.7) and whose area measurement is more then one standard deviation from the mean of particle area sizes on the plate is considered to be an anomaly. Examples of such artifacts include scratches on the plate or two colonies that have grown together due to excessive moisture on the plate. Any anomalies detected in an image are presented to the user in a list indicating the location of the artifact. An editable image of the plate is displayed for the user to manually remove artifacts using *ImageJ'*s built in editing tools. This artifact detection system is similar to the one offered in *Growth Detector*, which analyzes colony circularity to locate artifacts [17]. Once corrections are made, *CM Engine *assigns colony size values.

The colony assignment algorithm takes advantage of the fact that colonies are positioned on the plate in a regular grid, meaning that the approximate position where each colony should lie can be calculated based on the dimensions of the image. This information is correlated with the centroid coordinates that the *ImageJ *particle analysis function returns. For each cell of the grid, every particle whose centroid lies within the cell is analyzed, but only the one with the largest area is assigned as the measurement value of that cell. The information of every other particle within that cell is discarded.

In a feature unique to Standard mode, after all particles are processed a quality control algorithm is applied. This algorithm compares the number of particles assigned to the total number of particles in the image and is run to determine if the image has been successfully quantified. If these two values do not deviate more than 25% from one another, then the plate has been successfully quantified and the image is moved into the "measurement_passed" folder. Otherwise a quantification error has occurred and the image is moved to the "measurement_errors" directory.

#### *CM Engine *- Summation Measurement Mode

Depending on the way cells are deposited on plates, colony growth may not be represented by the largest particle within a cell of the grid layout, but instead by the growth of several particles within a cell. This type of growth may occur when colonies are pinned onto agar plates using blunt-tipped pins (typical of hand-pinned experiments) (**Figure **3a). In this situation, Standard mode is not optimal since it only considers the largest particle within a cell to represent colony growth. To accommodate this type of growth, we developed Summation mode. Like Standard mode, Summation mode applies a threshold to make a binary image, however all particles areas within each cell of the grid are summed to derive a growth value (**Figure **3c). In Summation mode, since all particle areas are summed, there is no attempt to remove artifacts (**Figure **3a).

#### *CM Engine *- Background Subtracted Measurement Mode

Cells deposited on agar plates from liquid cultures typically result in colonies having equivalent growth areas and growth differences are manifested by density variation. Such density information is lost when a binary threshold is applied, thus Background Subtracted mode was developed (**Figure **3a**and **3d). Our implementation of Background Subtracted mode is similar to a measurement method described by Jafari-Khouzani *et al*. for colorimetric assays [19]. In our implementation, images are first converted to 8-bit grayscale images. The background gray value of each cell of the grid is then determined by taking the median gray value of pixels in the four corners of a cell. This background gray value is subtracted from the cell and the remaining pixel values greater than 0 represent colony growth. The mean gray value of the cell is then reported as colony growth (**Figure **3d). Like Summation mode, Background Subtracted mode has the potential limitation that everything within a cell above the background value will contribute to the growth quantification of that cell, even if this includes stray marks or other artifacts. However, Background Subtracted mode has the benefit of not having to apply a threshold filter to the image prior to quantification. In addition, since the background value is calculated on a per cell basis, uneven lighting across a plate image does not affect quantification.

#### *CM Engine *- Cropping Algorithms

**Figure 4:**
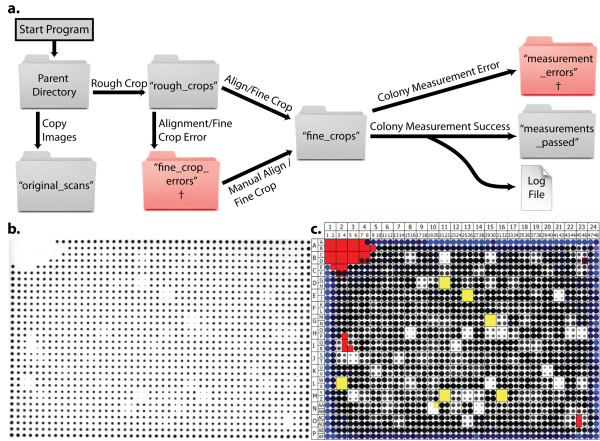
***CM *and *DR Engine *Details**. (**a**) Workflow of the *CM Engine*. Processing of images typically begins with multi-plate image files in a "Parent Directory." Prior to processing, *CM Engine *copies the multi-plate image files to the "original_scans" folder. The rough crop algorithm divides multi-plate images into individual plate images and moves them to the "rough_crops" folder. Rough cropped images that are successfully aligned and fine cropped are moved to the "fine_crops" folder. If unsuccessful, the affected images are moved to the "fine_crop_errors" folder. In most cases, the images in the "error" folder can be manually aligned and fine cropped and then moved to the "fine_crops" folder for processing in a subsequent run of the *CM Engine*. Next, colonies are measured, the results are appended to a log file and the images are moved to the "measurements_passed" folder. In the event of a measurement error, results are not appended to the log file and the image is moved to the "measurement_errors" folder (See **Additional File **1 for details). Red "error" folders are also marked with a dagger (†). (**b**) Image of a 1536 SDL screen plate containing four 2 × 2 replicates of each strain (384 strains total) that has been fine cropped by the *CM Engine*. (**c**) *DR Engine *normalized representation of the plate in **b **based on Standard mode measurements by the *CM Engine*. Circles are generated for each yeast colony on the plate. Circle size and color is directly correlated to normalized colony size. Colony color ranges from light gray for small colonies, to black for those that are equal to the plate median, to blue for those colonies larger than the plate median. Colonies highlighted in yellow are below 25% of the median. Colonies highlighted in red have been marked for exclusion from statistical consideration.

After the measurement mode and other analysis parameters are entered, *CM Engine *examines the structure of the parent directory to determine the format of the images to be quantified (multi-plate, "rough crops" or "fine crops"; see **Figure **2 and **Additional Files **1** and **2 for examples). Once this determination is made, *CM Engine *processes images in a stepwise fashion, moving them from a starting directory into specific folders as they are processed, eventually arriving in a final directory called "measurements_passed" (**Figure **4a). Multi-plate images are copied to a folder labeled "original_scans" and split into individual plate images, which are placed into a folder labeled "rough_crops". For proper processing the software requires multi-plate image backgrounds to be completely black with plates arranged as detailed in **Figure **2. *CM Engine *next applies a straightening algorithm to rotate each plate image in the "rough_crops" folder so that every row of colonies lies in a straight line. The straightening algorithm works by analyzing a selection of nine colonies that lie in a 3x3 square within the interior of a rough-cropped plate image. The coordinates of the center of each of these colonies (centroid coordinates) are determined and used to calculate the angles of the diagonals and edges of the square. In a perfect square, diagonals should be 45 or 135 degrees, vertical edges should be 90 degrees, and horizontal edges should be 0 degrees. The differences between the angles measured and the ideal angles of a perfect square are calculated and averaged to determine the direction and magnitude to rotate the image to straighten it. This process is repeated eight more times at other positions symmetrically distributed across the image. All resulting calculated angles are averaged and then used to rotate the image appropriately.

Following alignment, images are fine cropped so that borders are close to the edge of colony growth, and are moved into a folder labeled "fine_crops". The first step of the cropping algorithm determines the approximate width of a row (or column) of colonies as well as the average distance between the colonies. Once these measurements are determined, four rectangular selections are analyzed in the interior of the image. The shorter dimensions of the rectangles are equivalent to the sum of the average colony width and the average distance between colonies (i.e. encompasses one row and one interstitial space). The longer edge of each rectangle encompasses approximately three-quarters of the colonies in a row or column. Each rectangle is parallel to one of the edges of the image and is centered over a row or column of colonies. After initial positioning, each rectangle is moved independently towards the edge of the image in a stepwise process by a distance equivalent to the smaller dimension of each rectangle. At each step, the rectangles are queried for the presence of colonies within their borders. Once no colonies are found within the borders of a rectangle, its movement stops. The interior edge of that rectangle is considered to be the border of colonies. Using these algorithmically defined borders, the images are cropped and will contain a grid of colonies that lie in a specific number of rows and columns based on the density of the screen conducted (**Figure **4b). The integrity of this grid layout is validated by drawing a thin selection line between each row and column of colonies based on where they are expected to lie if they were properly aligned and cropped. The ImageJ "Measure" command is used to determine if any of these lines significantly overlap colonies. If they do not, the integrity of the grid layout is validated and the image is successfully cropped.

In our tests, over 98% (1313 of 1333) of plate images were successfully processed into properly rotated, fine-cropped images. If an error occurs with an image during this process (i.e., cropping or alignment), the image is moved into a folder labeled "fine_crop_errors". In this case, the images may be manually cropped, as described below. When fine cropping is completed, the *CM Engine *calculates colony growth using one of the three user-designated processing modes (Standard, Summation or Background Subtracted).

After successful colony measurement, images are moved to the "measurements_passed" folder. *CM Engine *produces a text-based log file that lists all colony measurements for every plate that was successfully processed. If errors occur at any point in image processing, additional log files are generated that describe which plates are affected and the type of error that occurs (error folders, **Figure **4a). Most images can be manually edited to overcome a particular error. For instance, images in the "fine_crop_errors" folder can be manually aligned and fine-cropped then moved by the user into the "fine_crops" folder. Upon re-running *CM Engine*, these manually-adjusted images are detected in the "fine_crops" folder, processed and the measurements are saved to a new log file allowing specific plates to be reanalyzed without having to re-process all plate images. Once all plates have been successfully measured, the user may upload the log file to the web-based *DR Engine *program. If multiple log files are present, they must be combined into one "master" log file before proceeding with *DR Engine*.

### Data Review Engine (*DR Engine*)

The *DR Engine *is a web-based application that normalizes raw screen data, provides a visual interface for removing common pinning errors and generates population statistics. The standard input for the *DR Engine *is the log file of growth data generated by the *CM Engine*, but other appropriately formatted quantitative data (e.g., fluorescence intensity, microtiter plate readings etc.) can be used. Strain information is associated with the log file by specifying a text-based key file with information about the strain library format. Key files for several strain libraries are available directly from the *ScreenMill *web interface, but custom key files for other libraries can be uploaded prior to analysis (see **Additional File **1 for formatting instructions).

The *DR Engine *normalizes uploaded data to allow colony sizes to be compared between plates, even if there is a general growth effect due to experimental treatment or media differences. Through the web interface, the user may choose to normalize data to the "Plate Median" (default) where each raw value is divided by the median growth value from its plate. The plate median growth value was chosen for normalization instead of the mean growth normalization method implemented in other work [20,21]. We chose the median value since it is less sensitive to fluctuation in cases where multiple positions on the plate are blank due to pinning errors or slow growth of specific strains. Alternatively, data can be normalized to the median growth value of "Designated Controls" located at specific positions on the plates. This is the preferred method if the user expects most of the tested strains to show a growth effect (e.g., when known affected strains from a larger screen are being validated). For this option, the positions of designated controls must be indicated in the key file (**See Additional File **1** - *Log and key file format information***). Finally, the user may choose to turn off normalization altogether ("Do NOT normalize") so that only raw growth values are used when calculating descriptive statistics. Further information about normalization is available in **Additional File **1. Normalized colony values are used by the *DR Engine *to determine the size and color of the circles that depict colonies in cartoon representations of plate images (**Figure **4c).

#### *DR Engine *- Data Exclusion Algorithms

Plate images rendered by the *DR engine *are color-highlighted to aid the review process. First, the data is evaluated for colonies that fall below 25% of the plate growth median and these are highlighted with a yellow background (**Figure **4c). Next, two algorithms are run to identify, and highlight in red, colonies to be excluded from statistical consideration. The first exclusion algorithm is employed to identify pinning errors in which a section of a plate was not inoculated (e.g., the upper left of **Figure **4b). The algorithm recursively selects colonies for exclusion by examining the size of each colony and its adjacent neighbors. If at least six out of eight neighboring colonies fall below 25% of the plate growth median (parameter 1) or at least two neighboring colonies have already been excluded (parameter 2), the program highlights the colony in red for exclusion. We tested values in the range of 2-7 for parameters 1 and 2 prior to assigning "6" and "2" as their optimized values (see **Additional File **3 for details). An additional algorithm to exclude spurious data is applied to plates that have 4 replicate samples of each strain arranged in 2 × 2 arrays (n.b., it does not run with other replicate configurations). This algorithm starts by comparing the value of each replicate to one another. Significant differences are determined by comparing the normalized size of each colony to the median of the 4 replicates. If a colony is within 45% of this median value, it is highlighted in red for exclusion. This range for exclusion was empirically chosen after analyzing the performance of many different ranges and determining that it most successfully excluded spurious data without affecting valid data (see **Additional File **3 for details).

We find that these two exclusion algorithms provide an appropriate check on spurious data for many types of pin-transfer experiments and is not available in any of the previously described software [16,17]. However, any algorithmically-defined exclusion can be changed in the user interface using a mouse click on the colony image to toggle the exclusion on or off. In addition, the behavior of the exclusion algorithms can be modified or turned off using the advanced settings of *DR Engine *(**Additional File **1).

#### *DR Engine *- Statistical Methods

After the data review is complete, plate normalized growth values are used to calculate p-values between strains on comparer and experimental plates. P-values are calculated using the normal distribution or t-test for parametric data, or the Mann-Whitney test for nonparametric data; the user selects the test that will be used at runtime. In addition, p-values may be calculated with or without multiple test correction using the Bonferroni method [22] (also selected at runtime). For small sample sizes, (e.g. ≤4 replicates) it is often difficult to determine if data is parametric (normal) or nonparametric. A graphing tool to generate histograms of raw data has been included on the *DR Engine *website to help evaluate data distribution. Histogram bin widths are calculated as in Shimizaki *et al *[23].

When the population as a whole (e.g. all the data on all the plates, or the collection of log(comparer/experimental) ratios) approximates a parametric distribution, the normal distribution method should be used to calculate p-values. Using this method, normalized growth values for the replicates of each strain are averaged and the resulting values are used to compute the log growth ratio. This ratio is defined as log(average comparer strain growth/average experimental plate growth). The population of log ratios for a screen experiment is assumed to exhibit a normal distribution, if they do not, it is up to users to transform their data appropriately before using this method within the *DR Engine*. Since the data are assumed to be normal, the mean and standard deviation are easily calculated. From these values, z-scores and two-tailed p-values are calculated. Two tailed p-values are calculated from z-scored using "uprob" function of the Perl Statistical Distributions module and multiplying the values returned by 2 [24].

The normal distribution method has the added benefit of automatically performing multiple test correction since each p-value is assigned based on the rank of the corresponding z-score within the entire distribution of data. As a result, Bonferroni correction of p-values should not be selected for this method.

As an alternative to the normal distribution method, the t-test method may be used when it is known that the value of each set of replicates independently is parametric. For this option, a Welch's t-test is carried out between comparer and experimental strain replicates [25,26]. T-test calculations are performed using the unpaired t-test for samples of unequal variances. T-scores and the degrees of freedom are calculated for each set of values using the traditional method [25]. A p-value is determined by passing these two values to the "tprob" function of the Perl Statistical Distributions module [24].

Finally, if the underlying distribution of the data is nonparametric or unknown, the Mann-Whitney test should be performed [27]. The Mann-Whitney test was implemented in Perl and calculates two-sided p-values. Exact p-values calculations are made unless there are more than 20 samples, in which case the normal approximation is used to calculate p-values from the U values (using the "uprob" function of the Perl Statistical Distributions module [24]) [28].

For both the Welch's t-test and the Mann-Whitney test it is recommended to apply Bonferonni correction the p-values.

#### *DR Engine *- Output

The data generated by the *DR Engine *web application (growth ratios, plate position, log ratios, z-scores, and p-values) are merged with the strain position information from the user-selected key file and displayed on a results page. These results are presented to the user in three tab-delimited downloadable text files. The first contains data from every strain that has been screened in the experiment (date-ScreenMillStats-all.txt). The second file contains information from those strains whose growth on the experimental plate is 50% or less than the control value (date-ScreenMillStats-positive-hits.txt). The third file contains information from strains whose growth on the experimental plate is greater than or equal to twice that of the control plate (date-ScreenMillStats-suppressors.txt).

### Statistics Visualization Engine (*SV Engine*)

**Figure 5:**
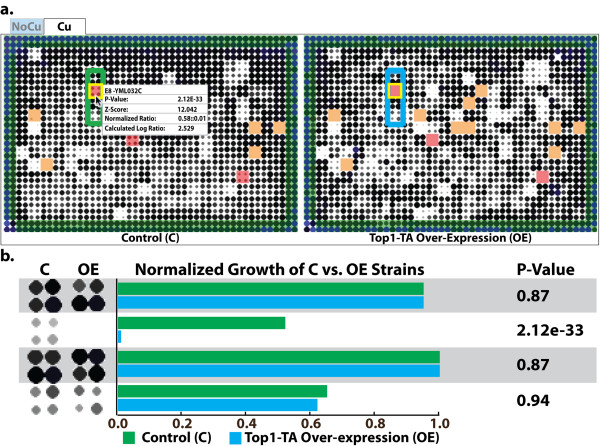
***SV Engine *Details**. (**a**) Typical display from the *SV Engine*. Cartoons represent control and experimental plates from a yeast SDL screen. Tabs above the plate cartoons indicate conditions, in this case 0 or 100 μM CuSO_4 _("NoCu" and "Cu"). Clicking a tab brings the corresponding plate cartoons to the front, in this case the "Cu" condition is visible. Strains are automatically highlighted based on their growth: Dead on control and experimental (orange), excluded (green), and significant (red). The plate position, ORF identifier, p-value, z-score, and growth ratios are displayed when the mouse pointer (black arrow) is over that strain. In this image, the pointer is hovering over position E8 (highlighted with a yellow box). (**b**) Magnified detail of the four strains (16 colonies) indicated by the green and blue boxes in **a**. Normalized growth values and their associated p-values are shown on the right.

The final element of *ScreenMill *is a novel web-based program called *SV Engine*, which has been developed to visualize the statistical data contained within the "ScreenMillStats-all.txt" file on a plate-by-plate basis. In this application, cartoon representations of a control and a corresponding experimental plate are presented side by side. The user can "mouse" over a set of colonies simultaneously highlighting both the control and corresponding experimental colonies. At the same time, the calculated statistical data for that strain and experimental treatment as well as identifier information present in the key file are displayed. Additionally, the tabbed interface enables the user to easily toggle views between plates allowing visualization of data from multiple experimental conditions (**Figure **5). The user can then select strains considered interesting by a mouse click. Statistical data from the user-selected strains may be downloaded to a Microsoft Excel file.

The *SV Engine *uses the p-value threshold calculated by the *DR Engine *to determine which strains to highlight as possibly significant. Although this value is the default, the user may adjust the threshold via the web interface. For instance, if users are interested in more subtle growth defects (e.g., only a 20% or greater difference between control and experimental strains) or more stringent defects (e.g., more than 70% growth difference), they can increase or decrease, respectively, the p-value threshold. Upon adjusting this threshold, the webpage is redrawn to highlight only those strains that reflect the changed parameter, thus allowing the user to immediately visualize the outcome.

## Results

*ScreenMill *has been extensively tested in our lab during the analysis of multiple SDL screens. A bottleneck in these high-throughput screens is the quantification and analysis of results. Using *ScreenMill*, we are able to eliminate this bottleneck, analyzing the data from up to 8 high-throughput screens in less than a day (almost 500,000 data).

One of the advantages of *ScreenMill*'s first tool, *CM Engine*, is that it contains three different measurement modes to allow accurate quantification of colonies derived from the most common cell deposition protocols (e.g., robotic pinning, hand pinning or liquid transfer, see **Figure **3). The ability to choose a specific measurement mode is unavailable in any published software and gives the *CM Engine *the flexibility to accurately measure a variety of colonies. To test that *CM Engine *properly quantifies colony sizes we compared the measurement modes of *CM Engine *to those previously described and readily available [16,17]. Images were quantified using all three modes of *CM Engine *and then visual comparisons were conducted between cartoons representations of the quantifications generated by *DR Engine*. In all cases *CM Engine *performed as well, and in some situations, much better than the other software (see **Additional File **4).

An additional benefit of *CM Engine *is the ability to accurately align and crop plate images. To validate the alignment and cropping algorithms we ran hundreds of different multi-plate images through *CM Engine*. As previously stated, over 98% (1313 of 1333) of plate images were successfully processed into properly rotated, fine-cropped images (data not shown).

To reduce costs to users, the *CM Engine *is written as an open source macro for the NIH *ImageJ *program. The *CM Engine *source code is extensively commented and it has an accompanying usage document. Together, the comments and the usage document clearly define all of the functions and variables contained within the macro (**Additional File **1**and **5). Additionally, since the *ImageJ *macro language itself is simply structured and well documented, the *CM Engine *source code can be easily interpreted to allow customization. As a result, users may change *CM Engine *as they wish, and submit updates to the code base to improve and extend the functionality of the macro.

The second tool in *ScreenMill *is the *DR Engine*, which provides a unique data review process to remove artifacts before generating the descriptive statistics of screen data. This web-based tool automatically renders cartoon representations of plates, greatly reducing the time and computing demands associated with uploading and displaying high-resolution digital images of plates processed by the *CM Engine*. The *DR Engine *also contains novel automatic and manual tools to exclude spurious data thereby improving the quality of the statistics generated. Furthermore, parameters of the *DR Engine *affecting plate normalization and exclusion algorithms can be modified through its web-interface to adapt the program to diverse data sets.

The third tool, the *SV Engine*, overlays statistics generated by the *DR Engine *on top of side-by-side cartoon representations of control and experimental plates. The cartoons and statistical calculations are based on normalized colony sizes calculated by the *DR Engine *and allow for comparison between plates, even if their overall growth differs. Users may refine lists of statistically significant data by toggling between views of different conditions, while also viewing relevant statistical information and strain identities. For example, the user may wish to limit data to only those having significant p-values across multiple experimental conditions. Thus the review process integrates the speed and ease of automation with the accuracy and flexibility of human decision-making to select only data of interest for further study.

## Conclusions

The *ScreenMill *software suite provides unique tools to measure, visualize, and review colony growth data from high-throughput screens. All *ScreenMill *components have been written using open-source software, eliminating any costs for academic users. Since *ScreenMill *is not proprietary, all algorithms are transparent and can be judged accordingly. Furthermore, the data files used by the individual *ScreenMill *components are defined in detail, so that each "*Engine*" can function independently of the others. For example, measurements from other sources can be formatted for use with the *DR *and *SV Engines*. Additionally, data visualization in the *DR and SV engines *is unique to *ScreenMill *and allows users to manipulate and review screen data in a manner previously unavailable. All of these features, combined with a simple user interface, make *ScreenMill *a valuable tool for analyzing high-throughput experiments.

## Availability and requirements

Project name: *ScreenMill*

Web page: http://www.rothsteinlab.com/tools/

Operating system: Platform independent

Programming languages: Perl, Ruby, ImageJ macro language, JavaScript, HTML and CSS

Other requirements: ImageJ 1.42 or higher (*CM Engine*). *DR *and *SV Engines *require JavaScript enabled browsers and have successfully been tested on Google Chrome 4.0, Safari 4.0 and Firefox 3.5

License: GNU GPL

Any restrictions to use by non-academics: license needed

## Competing interests

The authors declare that they have no competing interests.

## Authors' contributions

JCD designed and developed *ScreenMill *and wrote the paper. RJDR contributed to *ScreenMill *design and development. RJDR and RR designed the research and aided in evaluation of *ScreenMill *and writing of the manuscript. All authors read and approved the final version of the manuscript.

## Supplementary Material

Additional file 1 ***ScreenMill *- Instructions for use (Dittmar et al, additional file 1.pdf) **This file contains the supplementary information and figures referenced in the main text. This information includes: • *CM Engine *instructions for use ◦ Provides step-by-step instructions on how to use *CM Engine*. • *CM Engine *image orientation and naming conventions ◦ Describes how plates must be laid out in images and how to properly name them prior to processing with *CM Engine*. • *CM Engine *straightening and fine cropping algorithms ◦ Describes the straightening and fine cropping algorithms used when *CM Engine *processed images. • Log and key file format information ◦ Describes the way data is formatted in log and key files used/generated by *ScreenMill*. • *DR Engine *Normalization Options ◦ Describes the data normalization methods available in *DR Engine *• *DR Engine *advanced options ◦ Describes the advanced options available when using *DR Engine*. Click here for file

Additional File 2 **Image formats for use with *CM Engine *(Dittmar et al, additional file 2.zip)**. This file includes 5 files: • Additional file 2 - readme.pdf: included instructions on how to process the example images with *CM Engine *• Additional File 2 - multiplate setup.zip: a sample multi-plate image (.tif). • Additional File 2 - rough crops setup.zip: sample "rough cropped" images (.tif). • Additional File 2 - fine crops setup.zip: sample "fine cropped" images (.tif). • Additional File 2 - colonyAreas.txt: contains the quantification of the multi-plate image in *Additional File *2* - multiplate setup.zip *using *CM Engine - Standard mode*. Click here for file

Additional File 3 **Optimization of the parameters used in exclusion algorithms (Dittmar et al, additional file 3.zip) **This file contains 3 files: • Additional File 3 - Exclusion Algorithm Parameters.pdf: explains the analysis performed. • Additional File 3 - GlobalExclusionCartoons.zip: contains all of the cartoons generated when optimizing the global exclusion parameters • Additional File 3 - ReplicateExclusionCartoons.zip: contains all of the cartoons generated when optimizing the global exclusion parameters Click here for file

Additional File 4 **Comparison of Measurement Modes (Dittmar et al, additional file 4.zip)**. This file contains data comparing *CM Engine's *three measurement modes to *HT Colony Grid Analyzer *[16] and *Growth Detector *[17]. This file contains data in several files: • Additional File 4 - Comparison of Measurement Modes.pdf: A summary of the results and notes on how the analysis was performed. • Cartoons: Cartoon representations of raw measurements generated in *DR Engine *(.png file formats). • *CM Engine:* the original images analyzed by *CM Engine *(.tif). • Growth Detector Data: Original images (.tif files) and results of running Growth Detector (.png files). • HT Colony Grid Data: Original images (.jpg) and results of running HT Colony Grid Analyzer (.dat and .png files). Click here for file

Additional File 5 **Source code for *CM, DR *and *SV *Engines (Dittmar et al, additional file 5.zip)**. This file include two zip files: • ScreenMill - CM Engine.zip: contains two copies the *CM Engine*. One for ImageJ version 1.42, one for version 1.43+ (.txt files). • ScreenMill - DR and SV Engines.zip: contains the HTML, CSS, JavaScript, Image, and CGI (Perl) files needed to run *DR *and *SV Engines*. Click here for file
