# Supplementary material for: ScreenMill: A freely available software suite for growth measurement, analysis and visualization of high-throughput screen data
Source: BMC Bioinformatics. 2010 Jun 28;11:353. doi: 10.1186/1471-2105-11-353 (PMC2909220; doi:10.1186/1471-2105-11-353)
Supplement: Additional file 1 — ScreenMill - Instructions for use (Dittmar et al, additional file 1.pdf) This file contains the supplementary information and figures referenced in the main text. This information includes: • CM Engine instructions for use ◦ Provides step-by-step instructions on how to use CM Engine. • CM Engine image orientation and naming conventions ◦ Describes how plates must be laid out in images and how to properly name them prior to processing with CM Engine. • CM Engine straightening and fine cropping algorithms ◦ Describes the straightening and fine cropping algorithms used when CM Engine processed images. • Log and key file format information ◦ Describes the way data is formatted in log and key files used/generated by ScreenMill. • DR Engine Normalization Options ◦ Describes the data normalization methods available in DR Engine • DR Engine advanced options ◦ Describes the advanced options available when using DR Engine. [file 1471-2105-11-353-S1.PDF]

Additional file 1 – *ScreenMill* - Instructions for use

Instructions for *CM Engine* use

1) Download *CM Engine* from SourceForge (http://cm-engine.sourceforge.net/) or from the Rothstein Lab website (http://www.rothsteinlab.com/cm-engine.zip).

2) Download *ImageJ* (http://rsbweb.nih.gov/ij/).

3) Start *ImageJ*, install *CM Engine* as a macro (instructions here: http://rsbweb.nih.gov/ij/docs/menus/plugins.html#macros).

4) To start *CM Engine,* select it from *ImageJ*'s menu bar (Plugins -> Macros -> ScreenMill – CM Engine [c]).

5) Upon starting *CM Engine,* a dialog box will show up asking you to select a directory. This directory should be the parent directory of your images. The parent directory should only contain multi-plate scans or sub directories called "rough_crops" or "fine_crops". See **Supplementary Figure 3** and the Supplement labeled "***CM Engine* - Image orientation and naming conventions**" for further information.

6) After selecting a parent directory, you will be presented with an input dialog (**Supplementary Figure 1**, note, appearance may differ slightly based on operating system).

   Input dialog explanation:

   Colony Measurement mode: Default value = "Standard". For further information see supplement labeled **"*CM Engine - Colony* Measurement Modes"**.

   Plate Density: 384 or 1536 formats are currently supported.

   File name to save measurements to (log file): default = "colonyAreas".

   Running Mode: Default = "Standard". To see your images as they are being processed, along with popup messages that display relevant information as images are being processed, select "Debug". Note that debug mode is MUCH slower and memory intensive then Standard mode.

   Condition Input: This input is only present when multi-plate images containing conditions are detected by *CM Engine.* Enter identifiers for any conditions present in your multi-plate images. See the Supplement below labeled **"Image orientation and naming conventions"** for further information on conditions.

Incorrect Data: This input is only present when multi-plate images containing conditions are detected by *CM Engine.* Check this box if there is a mistake in the conditions displayed in the previous input.

7) After entering information in the input dialog box, click “Ok” and image processing begins. Please note that image processing is memory intensive. If *ImageJ* crashes due to an out of memory error, close *ImageJ*, restart it, and reload and rerun *CM Engine*. In the event of a crash, no data will be lost. When *CM Engine* is rerun, it will restart where it stopped at the time of the crash.

CM Engine - Image orientation and naming conventions

Image file names convey all the necessary information about plates for each *ScreenMill* component. The convention in *ScreenMill* is that the filenames of plate images convey information about experimental treatments as well as information about the strains contained on a plate. Therefore, images that are input into *CM Engine* must conform to orientation and naming conventions to ensure that comparisons between plate data are performed appropriately. Single plate images ("rough crops" and "fine crops") must be oriented horizontally with position A1 in the upper left-hand corner (see **Supplementary Figure 2**). Multi-plate image orientation is described in the next section.

For all images, file names are comprised of four parameters: a query identifier, a plate number(s), a condition(s) (if present) and a file extension. One of the queries is designated as the comparer (e.g., control) and is defined as the query to which all other queries will be compared (the user is prompted for this designation by the *DR Engine)*.

File name parameter definitions:

- Query = common identifier for all plates within one set. For example, if the plates in a set were control plates, then Query could be named "Control".
- Plate Number = typically an integer.
- Condition = descriptor for any additional treatment of the plate (e.g., drug or inducer).
- File Extension = file extension of the image. A lossless format, such as tif, is recommended, however, other image formats may be used (e.g., jpg or gif).

For example, a screen in which the *TOP1* gene and a mutant allele (*top1-TA*) were over-expressed in the yeast deletion library, some of the rough crop tif files could be named as follows:

- Control,1.tif
- Control,1,Cu.tif
- Control,2.tif
- Control,2,Cu.tif
- Top1,1.tif
- Top1,1,Cu.tif
- Top1,2.tif
- Top1,2,Cu.tif
- top1-TA,1.tif
- top1-TA,1,Cu.tif
- top1-TA,2.tif
- top1-TA,2,Cu.tif

In this example "Control," "Top1" and "top1-TA" are query identifiers, "1" and "2" are plate numbers and "Cu" is a condition. In this case, Cu indicates the presence of copper, which was used to induce the over-expression of either *TOP1* or *top1-TA*.

If "Control" were designated as the comparer in the *DR Engine*, then the following comparisons would be made:

- Control,1.tif to Top1,1.tif
- Control,1.tif to top1-TA,1.tif

- Control,1,Cu.tif to Top1,1,Cu.tif
- Control,2.tif to Top1,2.tif
- Control,2,Cu.tif to Top1,2,Cu.tif
- Control,1,Cu.tif to top1-TA,1,Cu.tif
- Control,2.tif to top1-TA,2.tif
- Control,2,Cu.tif to top1-TA,2,Cu.tif

In this example, to compare *TOP1* to *top1-TA*, *TOP1* would be designated the comparer in the *DR Engine*.

Other Single Plate Image Name Examples:

- "Cln3,4,Cu.tif" This is an example image name typical for a SDL screen. It identifies "Cln3" as the query in plate 4, and that copper is present in the plate (the Rothstein lab uses copper to induce gene expression when conducting SDL screens).
- "Control,plate1,.tif" In this example a generic label of "Control" has been entered as the query. This plate contains data from plate 1 and no condition is present.
- "glucose,1,.tif" In this example "glucose" is the query in plate 1 and no condition is present.

Multi-plate Images:

For proper multi-plate image processing by the *CM Engine*, plates must be in a grid with a small space separating them. All space between plates in multi-plate images must be black. Plates must be positioned vertically with coordinate A1 in the lower left hand corner. No orientation marks are required. Plate images are processed sequentially from left to right by row. (**Supplementary Figure 2**).
Images of rectangular agar plates should be named using the following convention:

- One plate in image = "Query,PlateNumber1*conditionSymbol*.fileExtension".
- Two plates in image = "Query,PlateNumber1*conditionSymbol*,PlateNumber2*conditionSymbol*. fileExtension".
- Three plates in image = "Query,PlateNumber1*conditionSymbol*,PlateNumber2*conditionSymbol*,PlateNumber3*conditionSymbol*. fileExtension".
- Etc.

Note that there are <u>no</u> spaces between the commas in the file names.
File name parameter definitions:

- Query = common identifier for all plates within one set. For example, if the plates in a set were control plates, then Query could be named "Control". Only <u>one</u> query can be present per image.
- PlateNumber = typically an integer.
- *conditionSymbol* = Optional. Any combination of +, ~, ^, #, @ (excluding commas, see below for examples). When *CM Engine* is run, these symbols are detected and the user is prompted to provide a descriptive label for each

condition.

- fileExtension = file extension of the image. A lossless format, such as tif, is recommended, however, other image formats may be used (e.g., jpg or gif).

Example multi-plate image names:

TOP1TA,3,3+,4,4+.tif

The query in this image is "TOP1TA". The plate numbers are 3 and 4. The *conditionSymbol* present is "+", which, in this example, indicates the presence of copper, while no *conditionSymbol* after a plate number indicates the absence of copper.

In **Supplementary Figure 2**, plate 3 would be in the top left corner, 3+ in the top right, 4 in the bottom left, and 4+ in the bottom right.

Control,1,1+,1~,1@.tif

The query in this image is "Control". The plate number is 1. The *conditionSymbols* present are "+", "~" and "@", which, in this example, indicate the presence of copper (+), nocodazole (~) and copper AND nocodazole (@).

To expand the number of conditions, symbols may be used in combination: e.g., ++, ~+, @^ etc. The *CM Engine* will prompt the user to label these *conditionSymbols* properly.

Log File Format

The log file generated by the *CM Engine* contains all of the quantitative information from an experiment. The data are organized by plate names, which identify the queries, plate numbers and conditions from each experiment. This organization is required for proper processing by *DR* and *SV Engines*. *CM Engine* automatically formats log files appropriately. However, data from other sources can be formatted for use with *DR* and *SV Engines*.

The rules for the log file format are as follows:

- Log files must be in plain text (".txt" file extension).
- The first line of a log file is a plate image file name, which must conform to the conventions outlined in the previous sections - query,plateNumber,condition. The next lines of the log file are the quantitative data for every position on that plate in column order (from left to right) using a separate line for each value. Data for additional plates follow on successive lines using this same format (See **Supplementary Figure 4**).
- Plates do not need to be listed in the log file in any particular order.
- Only 96, 384 and 1536 array formats are accepted.
- The array format in a log file should be homogenous (only 96 or only 384 or only 1536 array format).
- Blank positions in a plate should be listed in the log file as '0'.
- The number of lines in a log file should be equal to: number of plates in the log file + (number of plates in the log file x the array format).
    - For example, if your log file contained data for 5 plates in a 384 array format, it should contain 1925 lines (5 + [5 x 384]).

An example log file may be downloaded from the Rothstein lab website (http://www.rothsteinlab.com/tools/screen_mill/log_file_info).

***DR Engine* - Key File Format Information**

To associate the measurements in the log file with the appropriate contextual information (e.g., strain information), the *DR Engine* maps data within the log file to data contained in a text-based key file that reflects the configuration of the library. The key file minimally contains position information and an identifier for every strain in the library. Position information is defined by "plate," "row," and "column." The identifier (ID) is user-defined and can be information such as "strain name," "ORF," "gene" etc. Key files for several strain libraries are available directly from the *DR Engine* web interface, however custom key files for other libraries can be uploaded prior to analysis.

The rules for the key file format are as follows:

- Key files must be in plain text (".txt" file extension).
- Data in key files must be tab-delimited or comma-separated.
- The key file data starts with a header row that contains a label for every column of data. The header row does need to be the first line of the file; however, only the lines following the header are used to map the data in the log file.
- The header row must contain columns labeled "plate", "row" and "column" as well as one user-defined ID column.
    - Plate (plate number): A unique identifier used to identify the plates in the key file. Integer values are recommended to facilitate proper data sorting within the *DR Engine.*
    - Row: these values must be letters (e.g. A-Z then Aa-Az then Ba-Bz, etc.). These values identify the row positions of each plate.
    - Column: these values must be integers. These values identify the column positions in each plate.
    - ID column: user-defined column of information about the position, (e.g., strain name). The ID column is also used to indicate "Positive Control" locations in the "Designated Controls" normalization method. Upon uploading a key file to the *DR Engine*, the user is prompted to enter which column label indicates the ID column.
- The number of rows for each plate in the key file should be equivalent to the number of samples in each plate divided by the number of replicates.
    - For example, a screen conducted with 1536 samples per plate and in a 2 x 2 array (4 replicates) should contain 384 rows for each plate in the key file to match the number of unique samples on the plate.

- Every plate number in the log file must be present in the key file. For example, if the following query,plate,condition labels are in your log file:
  - Top1-TA,1,Cu.tif
  - Top1-TA,2a,Cu.tif
  - Top1-TA,Three,Cu.tif

  Then the following plate numbers <u>must</u> be present in your key file:

  - 1
  - 2a
  - Three
- If using "Designated Controls" as the normalization method, key file rows with the phrase *Positive Control* in the ID Column will be considered as control data.
- Key files may contain additional information simply by adding extra columns. The information contained in these columns is appended to the *DR Engine* output files.

An example key file may be found on the Rothstein lab website (http://www.rothsteinlab.com/tools/screen_mill/key_file_info).

DR Engine Normalization Options

Upon starting the *DR Engine*, there are three parameters found in the "Data Normalization Method" section that may be modified to alter the way *DR Engine* normalizes data.

The options are:

- Plate Median (selected by default): Data on a given plate are divided by the median value of all values on the plate to normalize. Unless changed in the advanced options zero values are ignored.
- Designate Controls: Data on a plate are normalized to the median value of all designated control data on the same plate. In a key file, rows with the phrase "Positive Control" in the ID column will be considered as control data.
- Do NOT Normalize: No data normalization is performed. Note that for display purposes data will be normalized via the "Plate Median" method, however, no normalization is performed when calculating population statistics

DR Engine Advanced Options

Upon starting the *DR Engine*, there are four parameters found in the "Advanced options" section that may be modified to alter the behavior of the program. These options give the user flexibility in the data analysis performed by the *DR Engine*.

The options are:

- Global Exclusion Iterations (default value = 3): Number of times to run the global exclusion algorithm described in **Results**. Select "0" to disable Global Exclusion. Disabling this algorithm will prevent data from being highlighted for exclusion by the global exclusion algorithm, although the user may still manually exclude data as described in **Results.**

- Run Replicate Exclusion (default value = Yes): Allows for the replicate exclusion algorithm, described in **Results**, to be disabled. This parameter only affects processing when analyzing data from a screen with 4 replicates (2 x 2). Disabling this algorithm prevents the exclusion of data based on the comparative growth within a set of 4 replicates.

- Yellow Highlight Cutoff (default value = 25% of plate normalization value): Cutoff below which data will be highlighted in yellow in cartoon representations of plates. Modifying this parameter will affect the global exclusion algorithm (**See Results**). Increasing this value may increase the number of data highlighted in yellow and therefore may increase the number of data highlighted in red for exclusion. Decreasing it will have the opposite effect.

- Avoid 0 Values (default value = yes): This parameter affects the way data are normalized to the "Plate Median." Uncheck this box to include, "zero" values in the plate median calculation.

Supplementary Figure 1 – Screen Shot of the *CM Engine* Input Dialog

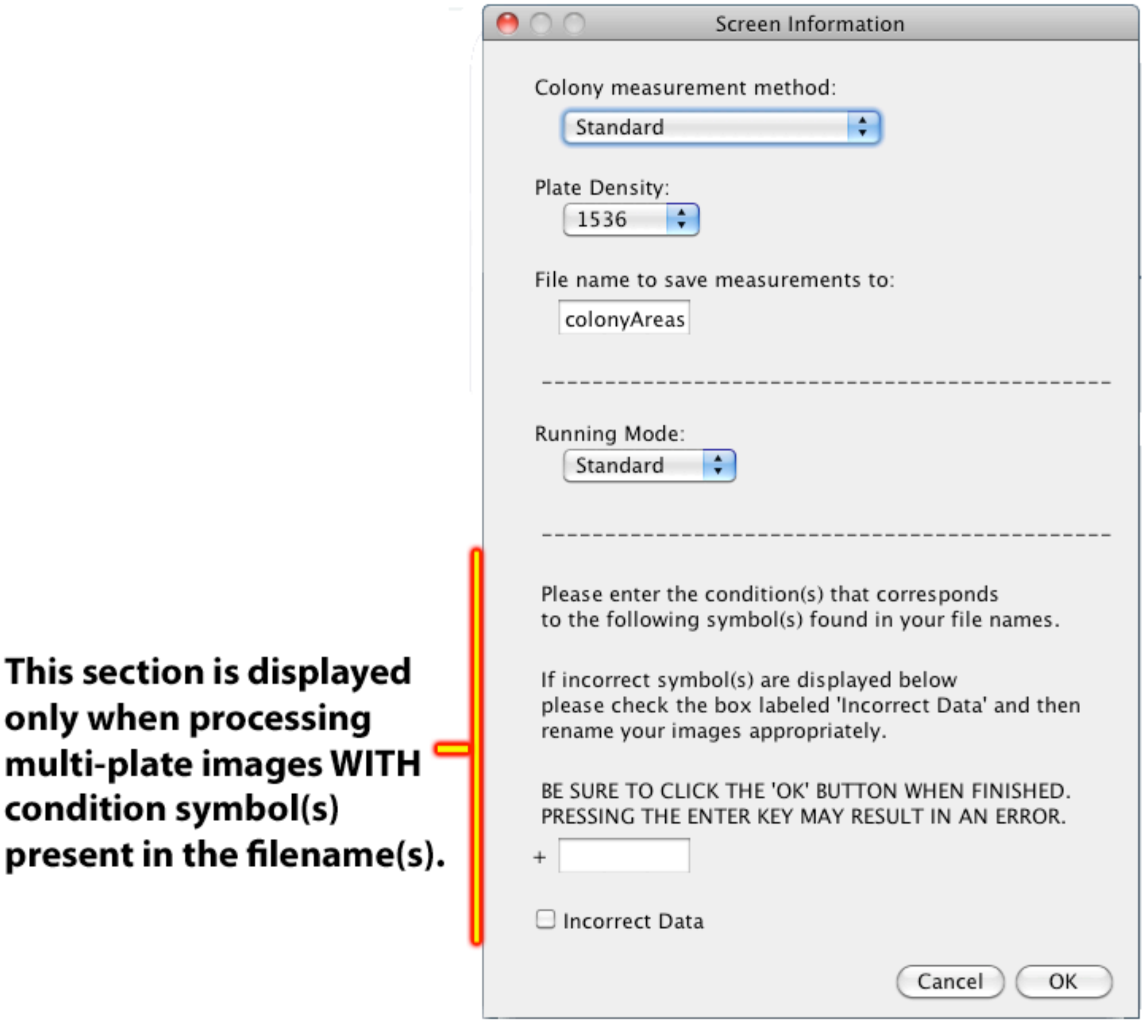

Supplementary Figure 2 - Elaboration of *CM Engine* Directory Structure

Directory Setup for multi-plate images

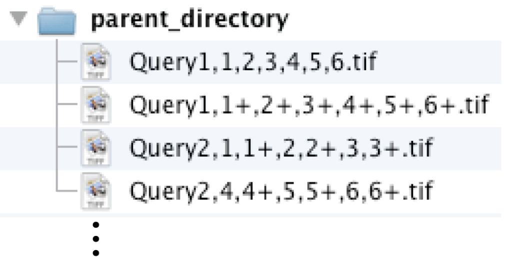

Directory Setup for rough crops

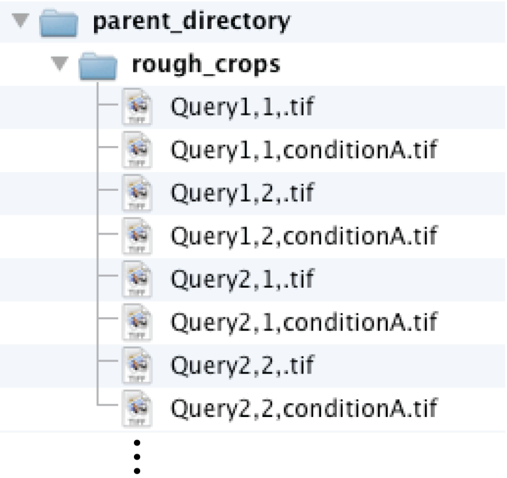

Directory Setup for fine crops

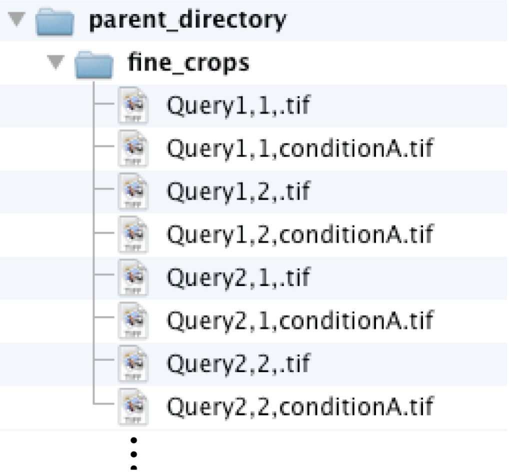

Directory Structure after run

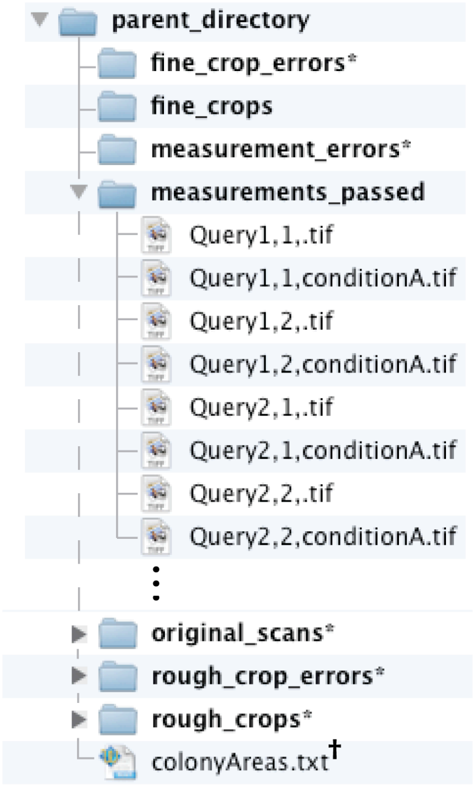

---

† log file

* depending on conditions, this file/directory may not be present

Supplementary Figure 3 - Elaboration of Log File Format

Line number	Lines from log file	Column position from corresponding plate
1	`TOP1,3,.tif`	
2	**457**	A1
3	**378**	B1
4	**323**	C1
•	•	•
•	•	•
•	•	•
383	**92**	N24
384	**385**	O24
385	**413**	P24
386	`TOP1,3,Cu.tif`	
387	**416**	A1
388	**329**	B1
389	**311**	C1
•	•	•
•	•	•
•	•	•
768	**85**	N24
769	**358**	O24
770	**406**	P24
771	`TOP1,4,.tif`	
772	**231**	A1
•	•	•
•	•	•
•	•	•
