# Supplementary material for: ScreenMill: A freely available software suite for growth measurement, analysis and visualization of high-throughput screen data
Source: BMC Bioinformatics. 2010 Jun 28;11:353. doi: 10.1186/1471-2105-11-353 (PMC2909220; doi:10.1186/1471-2105-11-353)

Additional file 2 – Sample images for use with *ScreenMill – CM Engine*

ScreenMill – CM Engine has the ability to process images in three different formats, multi-plate, “rough crops” and “fine crops”.

There are three .zip files included with this file, each which contains images in a different format:

- Additional File 2 - multiplate setup.zip – contains one multi-plate image, query1,1,1+,2,2+,3,3+,4,4+.tif. This plate contains 8 plates, see **Figure S1** for an explanation of the plate layout. In this image a condition is present in some of the plates as indicated by the ‘+’ symbol.
- Additional File 2 - rough crops setup.zip – contains eight images of the same plates in the multi-plate image described above. In these images the ‘+’ condition indicated in some plates has been translated into ‘Cu’, indicating the presence of $CuSO_4$ on those plates
- Additional File 2 - fine crops setup.zip – contains the same data as *Additional File 2 - rough crops setup.zip* except that these images are fine cropped instead of rough cropped

Each zip file contains files in the proper orientation and directories in order to be processed successfully with *CM Engine*. Each zip file contains a directory called “parentDirectory”. In all cases, this is the directory that must be selected when running *CM Engine* for proper processing to occur.

There is an additional file included with this file called *colonyAreas.txt*. This file contains the quantification results of running *CM Engine* on the multi-plate image in *Additional File 2 - multiplate setup.zip.* The quantifiactions were obtained by running *CM Engine* using the parameters found in **Figure S2**

Supplementary Figure 1 – Explanation of plates in query1,1,1+,2,2+,3,3+,4,4+.tif

For each plate position A1 is indicated with a red circle. Some plates contain the condition 'Cu', which was enter as the condition indicated by the '+' symbol when *CM Engine* was run (see **Figure S2**).

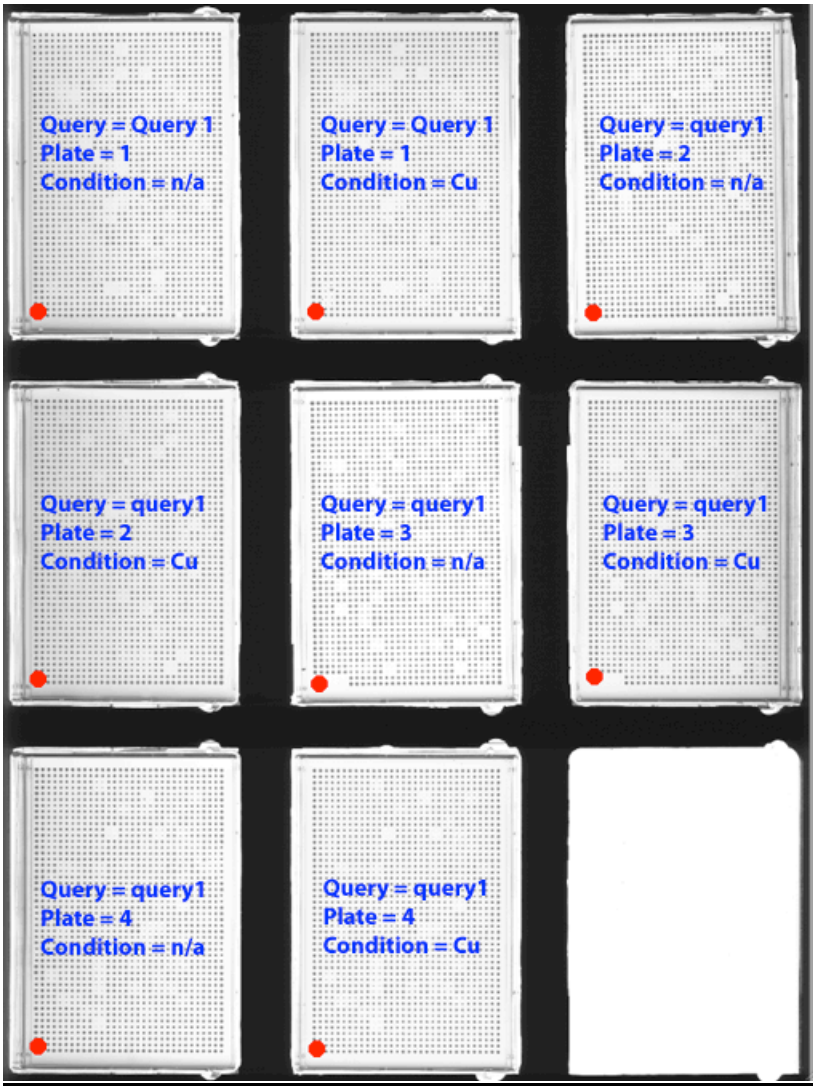

Supplementary Figure 2 – Parameters entered when running *CM Engine* with sample images provided.

Note that 'Cu' was enter as the condition corresponding to the '+' symbol in the filename of the multi-plate image.

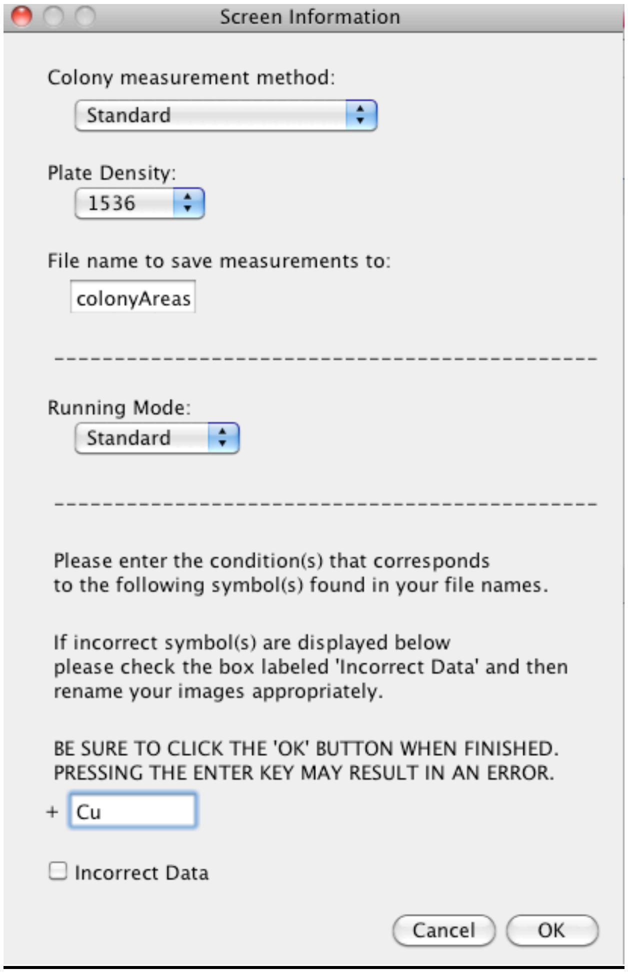

Supplement: Additional File 2 — Image formats for use with CM Engine (Dittmar et al, additional file 2.zip). This file includes 5 files: • Additional file 2 - readme.pdf: included instructions on how to process the example images with CM Engine • Additional File 2 - multiplate setup.zip: a sample multi-plate image (.tif). • Additional File 2 - rough crops setup.zip: sample "rough cropped" images (.tif). • Additional File 2 - fine crops setup.zip: sample "fine cropped" images (.tif). • Additional File 2 - colonyAreas.txt: contains the quantification of the multi-plate image in Additional File 2 - multiplate setup.zip using CM Engine - Standard mode. [file 1471-2105-11-353-S2.ZIP › Additional file 2 ΓÇô readme.pdf]
