# Supplementary material for: ScreenMill: A freely available software suite for growth measurement, analysis and visualization of high-throughput screen data
Source: BMC Bioinformatics. 2010 Jun 28;11:353. doi: 10.1186/1471-2105-11-353 (PMC2909220; doi:10.1186/1471-2105-11-353)

Additional File 3 - Optimization of the parameters used in exclusion algorithms

Global Exclusion Algorithm

The global algorithm recursively selects colonies for exclusion by examining the size of each colony and its adjacent neighbors. If at least six out of eight neighboring colonies fall below 25% of the plate growth median (parameter 1) or at least two neighboring colonies have already been excluded (parameter 2), the program highlights the colony in red for exclusion. Values in the range of 2-7 were assigned to parameter 1 and 2 and cartoon renderings of two samples plates were generated using *DR Engine*. After examining all cartoons values of 6 and 2 as the optimized parameter values (**Figure S1**). Images of the cartoons of all other parameter values are included with this file (**Additional File 3 – GlobalExclusionCartoons.zip**). In the image file names the first number indicates parameter 1 and then second indicates parameter two.

Replicate Exclusion Algorithm

The replicate exclusion algorithm is run after the global exclusion algorithm to exclude spurious data is applied to plates that have 4 replicate samples of each strain arranged in 2 x 2 arrays (n.b., it does not run with other replicate configurations). The algorithm starts by comparing the value of each replicate to one another. Significant differences are determined by comparing the normalized size of each colony to the median of the 4 replicates. If a colony is within 45% of this median value, it is highlighted in red for exclusion (parameter 3). In order to determine the optimized parameter value of 45%, values ranging from 5% to 95% were tested. Cartoon renderings of a sample plate were once again generated and analyzed to determine to most appropriate parameter value. A sample of these cartoons can be seen in **Figure S2**. **Additional File 3 – ReplicateExclusionCartoons.zip**, included with this file, contains images of all cartoons generated. The parameter value used to generate each cartoon is indicated in the filenames.

Supplementary Figure 1 – Global Exclusion Parameter Optimization

In this figure the bottom row of plates shows the optimal exclusion parameters of [6,2]. These values correctly exclude pinning errors while leaving normal blanks in the plate (e.g. plate 1 positions F4 and F5) untouched.

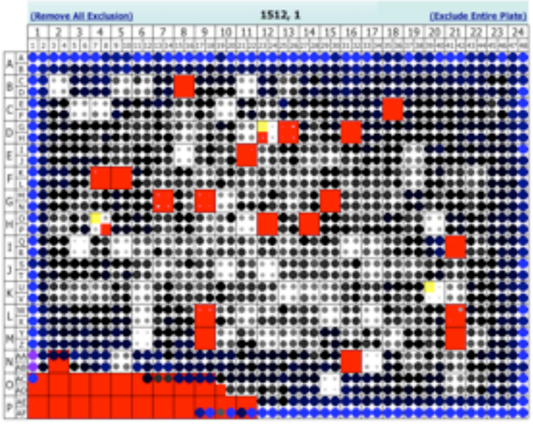

Plate 1, Parameters = [2,2]

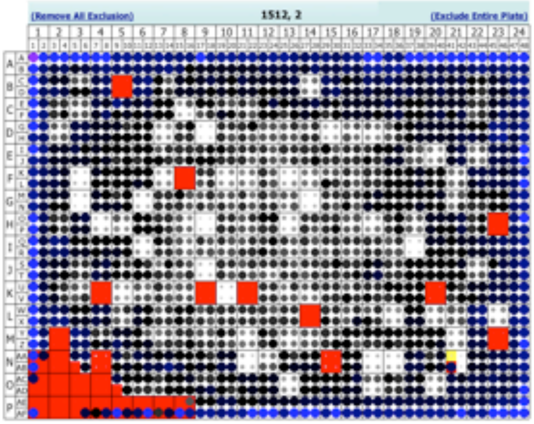

Plate 2, Parameters = [2,2]

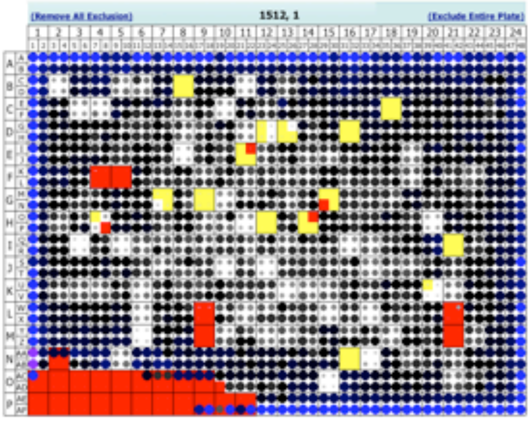

Plate 1, Parameters = [4,2]

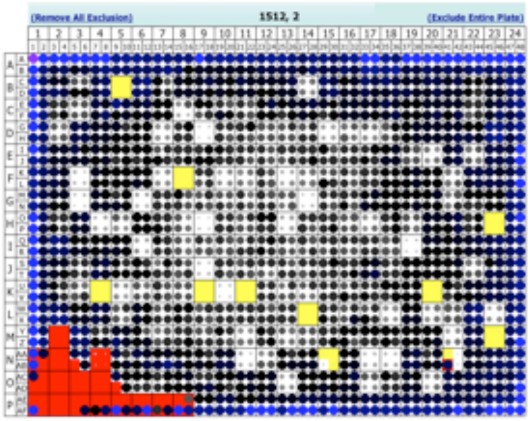

Plate 2, Parameters = [4,2]

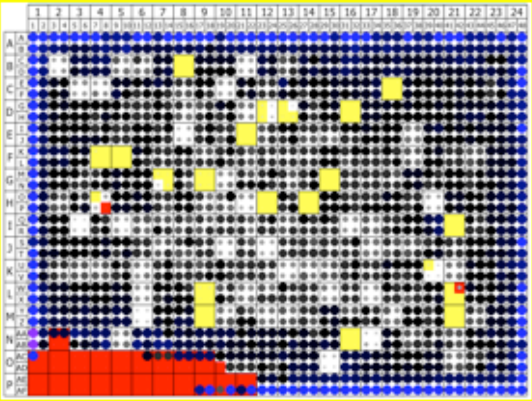

Plate 1, Parameters = [6,2]

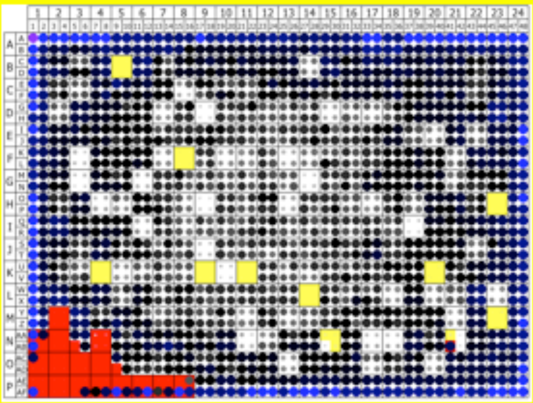

Plate 2, Parameters = [6,2]

<u>**Supplementary Figure 2 – Replicate Exclusion Parameter Optimization**</u>

In this figure the middle row shows the colonies excluded using the optimal exclusion parameter of 45%. This values correctly exclude spurious values while leaving normal values untouched.

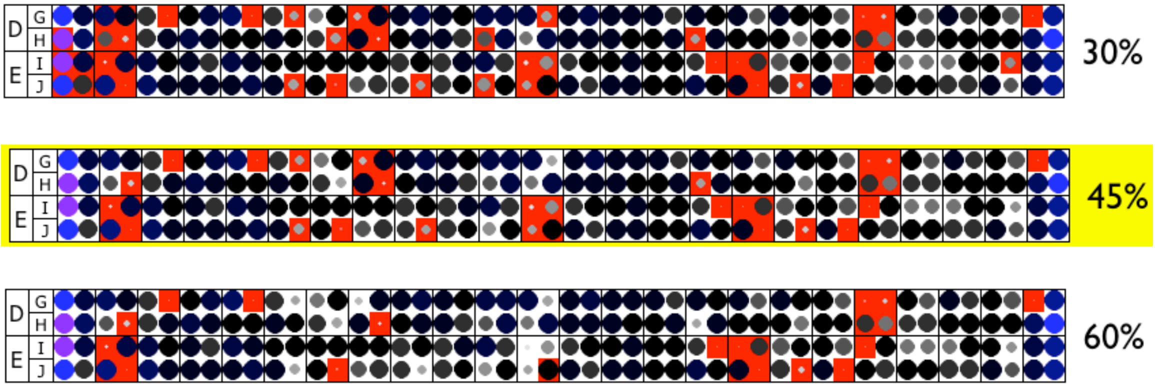

Supplement: Additional File 3 — Optimization of the parameters used in exclusion algorithms (Dittmar et al, additional file 3.zip) This file contains 3 files: • Additional File 3 - Exclusion Algorithm Parameters.pdf: explains the analysis performed. • Additional File 3 - GlobalExclusionCartoons.zip: contains all of the cartoons generated when optimizing the global exclusion parameters • Additional File 3 - ReplicateExclusionCartoons.zip: contains all of the cartoons generated when optimizing the global exclusion parameters [file 1471-2105-11-353-S3.ZIP › Additional File 3 - Exclusion Algorithm Parameters.pdf]
