# Supplementary material for: ScreenMill: A freely available software suite for growth measurement, analysis and visualization of high-throughput screen data
Source: BMC Bioinformatics. 2010 Jun 28;11:353. doi: 10.1186/1471-2105-11-353 (PMC2909220; doi:10.1186/1471-2105-11-353)
Supplement: Additional File 4 — Comparison of Measurement Modes (Dittmar et al, additional file 4.zip). This file contains data comparing CM Engine's three measurement modes to HT Colony Grid Analyzer [16] and Growth Detector [17]. This file contains data in several files: • Additional File 4 - Comparison of Measurement Modes.pdf: A summary of the results and notes on how the analysis was performed. • Cartoons: Cartoon representations of raw measurements generated in DR Engine (.png file formats). • CM Engine: the original images analyzed by CM Engine (.tif). • Growth Detector Data: Original images (.tif files) and results of running Growth Detector (.png files). • HT Colony Grid Data: Original images (.jpg) and results of running HT Colony Grid Analyzer (.dat and .png files). [file 1471-2105-11-353-S4.ZIP › Additional File 4 ΓÇô Comparison of Measurement Modes.pdf]

Additional File 4 – Comparison of Measurement Modes

To evaluate the performance of *CM Engine,* we quantified several plates with the three different measurement modes contained within *CM Engine* and with *Growth Detector* [1] and *HT Colony Grid Analyzer* [2].

The results of the analysis are included with this file:

- Cartoons – contains images of cartoon renditions of plates created with *DR Engine* base on the measurements calculated with the different programs. Image filenames are in the format: methodUsed – PlateName (e.g. CM_BackgroundSubtracted-384,1 is a cartoon of plate "384,1" quantified using the background subtracted method of *CM Engine*).

- CM Engine – contains copies of the fine-cropped images that were processed using *CM Engine*.

- Growth Detector Data – contains the data used and generated by *Growth Detector*. Files containing the phrase 'true colonies' were produced by *Growth Detector* and presumably indicate the colonies recognized. The remaining files are copies of the ones that served as input for *Growth Detector.* The quantifications *Growth Detector* returned for the images analyzed can be found in the folder labeled "Results", see below.

- HT Colony Grid Data – contains the data used and generated by *HT Colony Grid Analyzer*. Most images were analyzed by manually selecting the top row of colonies from within the program. Image 1536-3.jpg was analyzed via the manual and automatic methods available within the program. Filenames containing the phrase "mask" are "masked images" that *HT Colony Grid Analyzer* returns after manually processing an image. Files with the .dat file extension contain the measurements returned by *HT Colony Grid Analyzer*. The remaining files are copies of the ones that served as input for *HT Colony Grid Analyzer*.

- Results – contains text files that contain the results of all the images quantified using each method.

Analysis Conclusions

To assess which measurement method produces the most accurate measurements, cartoon rendering of images of plates were visually compared to the original images. In all cases *CM Engine* performed as well, if not better then *Growth Detector* or *HT Colony Grid Analyzer*.

Notes

- Images in 1536 format were not analyzed with *Growth Detector* as it only supports quantifying images in the 384 format. In addition, the 'true colony' images returned by *Growth Detector* depict colonies in the proper

orientation, however the quantifications returned were skewed such that images were rotated 180° when rendered in cartoons by *DR Engine.*

- When running *CM Engine* in Standard mode, the following images were not processed (*CM Engine* returned an error message):
    - 384,1 (reason: did not threshold well)
    - 384,5 (reason: number of particles assigned differed too much from those detected)
- When running *CM Engine* in Summation mode, image 384,1 was not quantified (*CM Engine* returned an error message) because it did not threshold well.

References:

1. Memarian N, Jessulat M, Alirezaie J, Mir-Rashed N, Xu J, Zareie M, Smith M, Golshani A: **Colony size measurement of the yeast gene deletion strains for functional genomics**. *BMC Bioinformatics* 2007, **8**:117.
2. **HT Colony Grid Analyzer** [http://sourceforge.net/project/showfiles.php?group_id=163953]
