# Supplementary figures and images for: ScreenMill: A freely available software suite for growth measurement, analysis and visualization of high-throughput screen data
Source: BMC Bioinformatics. 2010 Jun 28;11:353. doi: 10.1186/1471-2105-11-353 (PMC2909220; doi:10.1186/1471-2105-11-353)

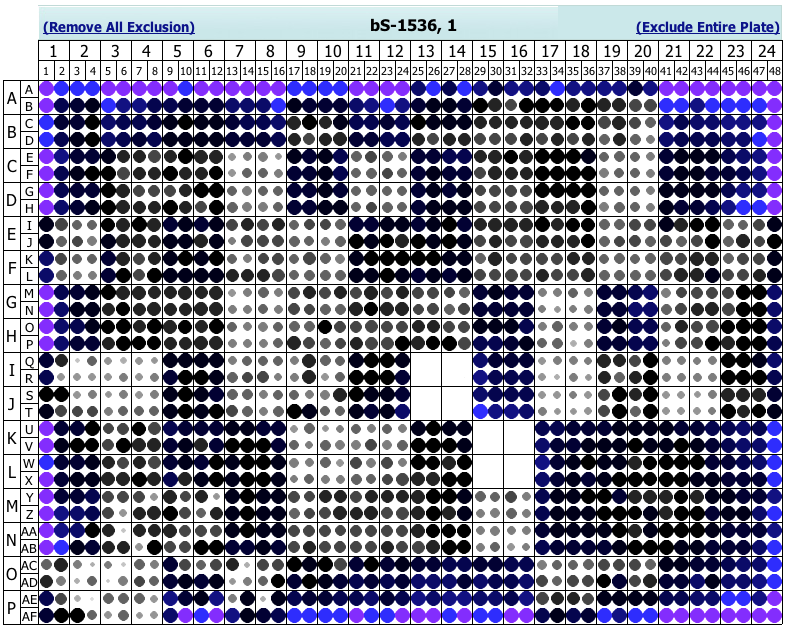

Supplement: Additional File 4 — Comparison of Measurement Modes (Dittmar et al, additional file 4.zip). This file contains data comparing CM Engine's three measurement modes to HT Colony Grid Analyzer [16] and Growth Detector [17]. This file contains data in several files: • Additional File 4 - Comparison of Measurement Modes.pdf: A summary of the results and notes on how the analysis was performed. • Cartoons: Cartoon representations of raw measurements generated in DR Engine (.png file formats). • CM Engine: the original images analyzed by CM Engine (.tif). • Growth Detector Data: Original images (.tif files) and results of running Growth Detector (.png files). • HT Colony Grid Data: Original images (.jpg) and results of running HT Colony Grid Analyzer (.dat and .png files). [file 1471-2105-11-353-S4.ZIP › Cartoons/1536/CM_BackgroundSubtracted-1536,1.png]

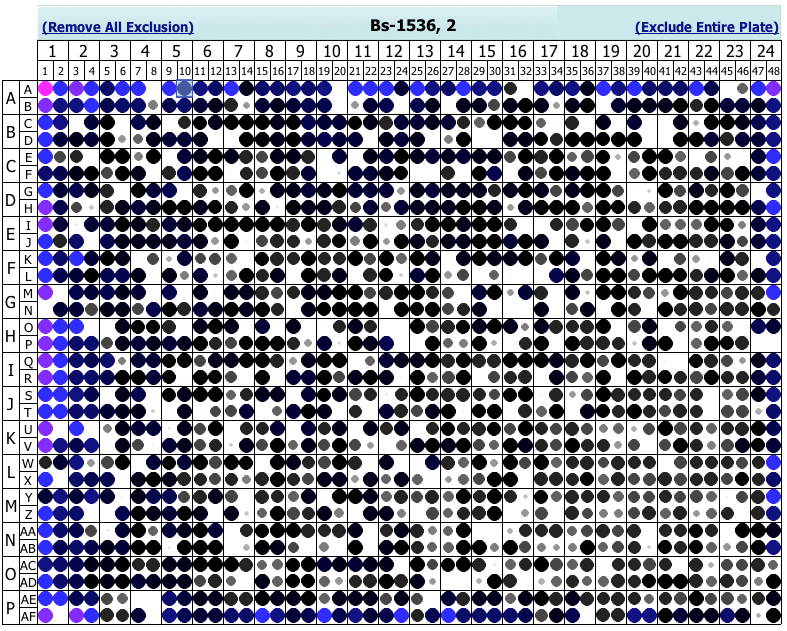

Supplement: Additional File 4 — Comparison of Measurement Modes (Dittmar et al, additional file 4.zip). This file contains data comparing CM Engine's three measurement modes to HT Colony Grid Analyzer [16] and Growth Detector [17]. This file contains data in several files: • Additional File 4 - Comparison of Measurement Modes.pdf: A summary of the results and notes on how the analysis was performed. • Cartoons: Cartoon representations of raw measurements generated in DR Engine (.png file formats). • CM Engine: the original images analyzed by CM Engine (.tif). • Growth Detector Data: Original images (.tif files) and results of running Growth Detector (.png files). • HT Colony Grid Data: Original images (.jpg) and results of running HT Colony Grid Analyzer (.dat and .png files). [file 1471-2105-11-353-S4.ZIP › Cartoons/1536/CM_BackgroundSubtracted-1536,2.png]

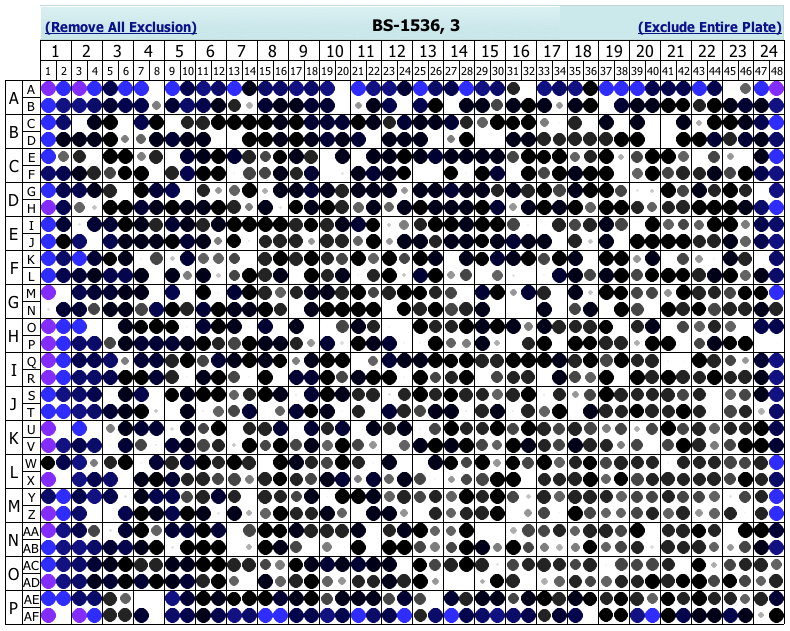

Supplement: Additional File 4 — Comparison of Measurement Modes (Dittmar et al, additional file 4.zip). This file contains data comparing CM Engine's three measurement modes to HT Colony Grid Analyzer [16] and Growth Detector [17]. This file contains data in several files: • Additional File 4 - Comparison of Measurement Modes.pdf: A summary of the results and notes on how the analysis was performed. • Cartoons: Cartoon representations of raw measurements generated in DR Engine (.png file formats). • CM Engine: the original images analyzed by CM Engine (.tif). • Growth Detector Data: Original images (.tif files) and results of running Growth Detector (.png files). • HT Colony Grid Data: Original images (.jpg) and results of running HT Colony Grid Analyzer (.dat and .png files). [file 1471-2105-11-353-S4.ZIP › Cartoons/1536/CM_BackgroundSubtracted-1536,3.png]

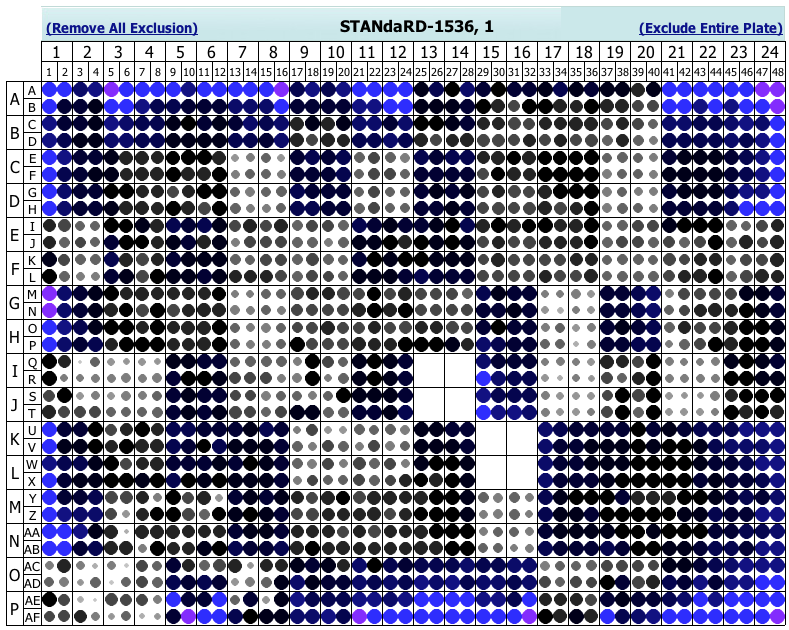

Supplement: Additional File 4 — Comparison of Measurement Modes (Dittmar et al, additional file 4.zip). This file contains data comparing CM Engine's three measurement modes to HT Colony Grid Analyzer [16] and Growth Detector [17]. This file contains data in several files: • Additional File 4 - Comparison of Measurement Modes.pdf: A summary of the results and notes on how the analysis was performed. • Cartoons: Cartoon representations of raw measurements generated in DR Engine (.png file formats). • CM Engine: the original images analyzed by CM Engine (.tif). • Growth Detector Data: Original images (.tif files) and results of running Growth Detector (.png files). • HT Colony Grid Data: Original images (.jpg) and results of running HT Colony Grid Analyzer (.dat and .png files). [file 1471-2105-11-353-S4.ZIP › Cartoons/1536/CM_Standard-1536,1.png]

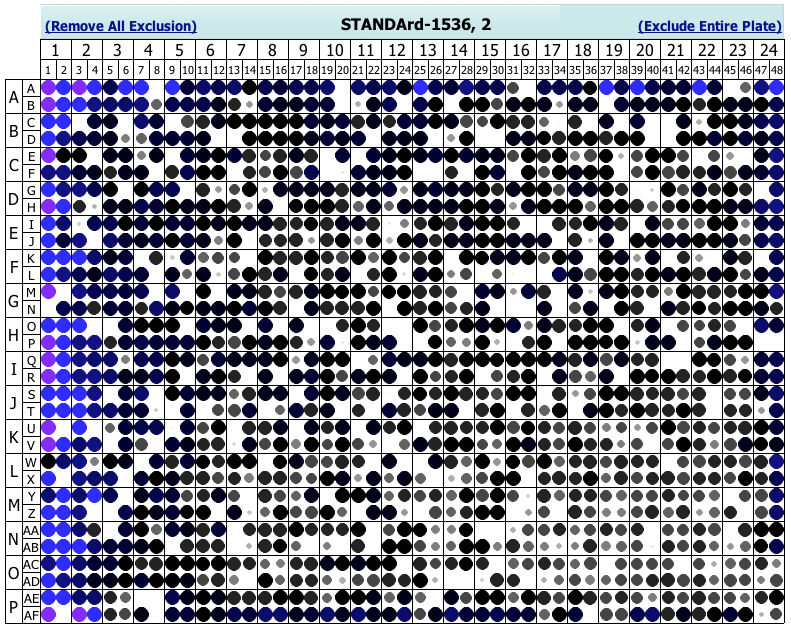

Supplement: Additional File 4 — Comparison of Measurement Modes (Dittmar et al, additional file 4.zip). This file contains data comparing CM Engine's three measurement modes to HT Colony Grid Analyzer [16] and Growth Detector [17]. This file contains data in several files: • Additional File 4 - Comparison of Measurement Modes.pdf: A summary of the results and notes on how the analysis was performed. • Cartoons: Cartoon representations of raw measurements generated in DR Engine (.png file formats). • CM Engine: the original images analyzed by CM Engine (.tif). • Growth Detector Data: Original images (.tif files) and results of running Growth Detector (.png files). • HT Colony Grid Data: Original images (.jpg) and results of running HT Colony Grid Analyzer (.dat and .png files). [file 1471-2105-11-353-S4.ZIP › Cartoons/1536/CM_Standard-1536,2.png]

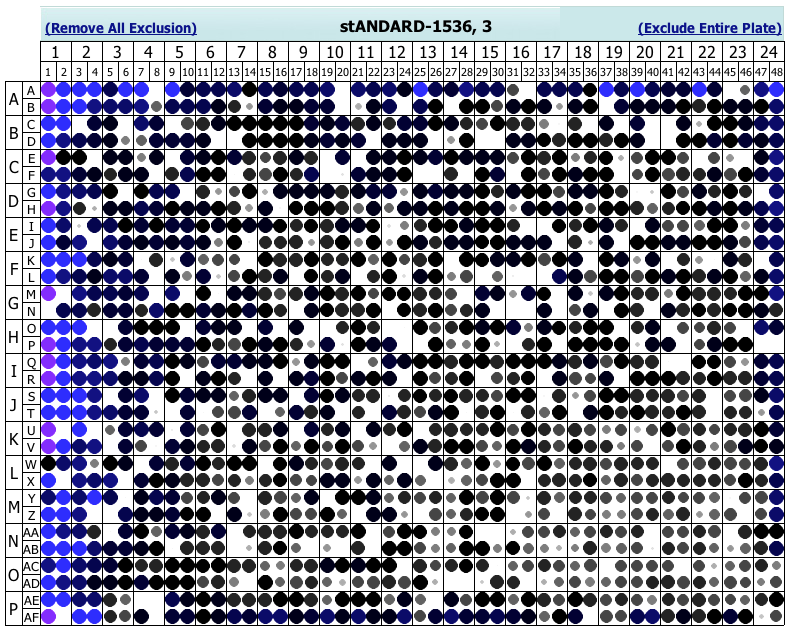

Supplement: Additional File 4 — Comparison of Measurement Modes (Dittmar et al, additional file 4.zip). This file contains data comparing CM Engine's three measurement modes to HT Colony Grid Analyzer [16] and Growth Detector [17]. This file contains data in several files: • Additional File 4 - Comparison of Measurement Modes.pdf: A summary of the results and notes on how the analysis was performed. • Cartoons: Cartoon representations of raw measurements generated in DR Engine (.png file formats). • CM Engine: the original images analyzed by CM Engine (.tif). • Growth Detector Data: Original images (.tif files) and results of running Growth Detector (.png files). • HT Colony Grid Data: Original images (.jpg) and results of running HT Colony Grid Analyzer (.dat and .png files). [file 1471-2105-11-353-S4.ZIP › Cartoons/1536/CM_Standard-1536,3.png]

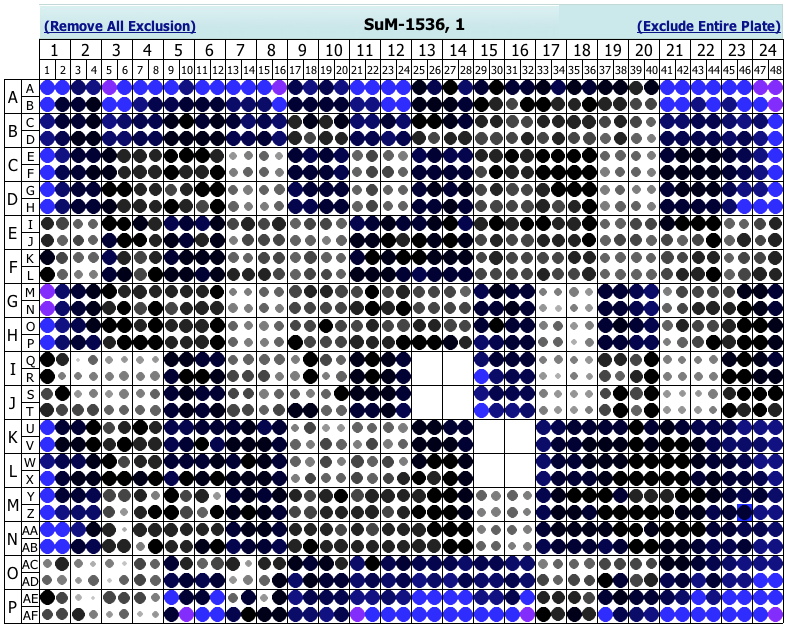

Supplement: Additional File 4 — Comparison of Measurement Modes (Dittmar et al, additional file 4.zip). This file contains data comparing CM Engine's three measurement modes to HT Colony Grid Analyzer [16] and Growth Detector [17]. This file contains data in several files: • Additional File 4 - Comparison of Measurement Modes.pdf: A summary of the results and notes on how the analysis was performed. • Cartoons: Cartoon representations of raw measurements generated in DR Engine (.png file formats). • CM Engine: the original images analyzed by CM Engine (.tif). • Growth Detector Data: Original images (.tif files) and results of running Growth Detector (.png files). • HT Colony Grid Data: Original images (.jpg) and results of running HT Colony Grid Analyzer (.dat and .png files). [file 1471-2105-11-353-S4.ZIP › Cartoons/1536/CM_Summation-1536,1.png]

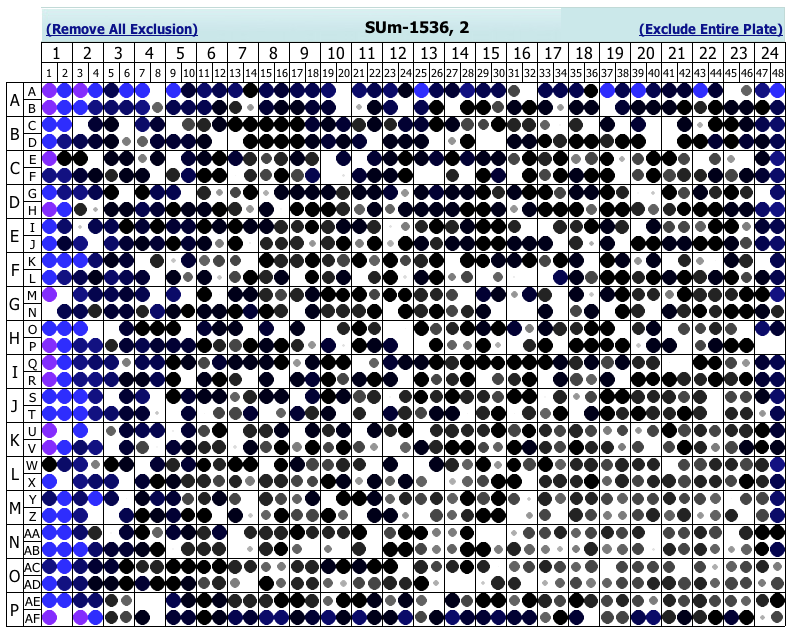

Supplement: Additional File 4 — Comparison of Measurement Modes (Dittmar et al, additional file 4.zip). This file contains data comparing CM Engine's three measurement modes to HT Colony Grid Analyzer [16] and Growth Detector [17]. This file contains data in several files: • Additional File 4 - Comparison of Measurement Modes.pdf: A summary of the results and notes on how the analysis was performed. • Cartoons: Cartoon representations of raw measurements generated in DR Engine (.png file formats). • CM Engine: the original images analyzed by CM Engine (.tif). • Growth Detector Data: Original images (.tif files) and results of running Growth Detector (.png files). • HT Colony Grid Data: Original images (.jpg) and results of running HT Colony Grid Analyzer (.dat and .png files). [file 1471-2105-11-353-S4.ZIP › Cartoons/1536/CM_Summation-1536,2.png]

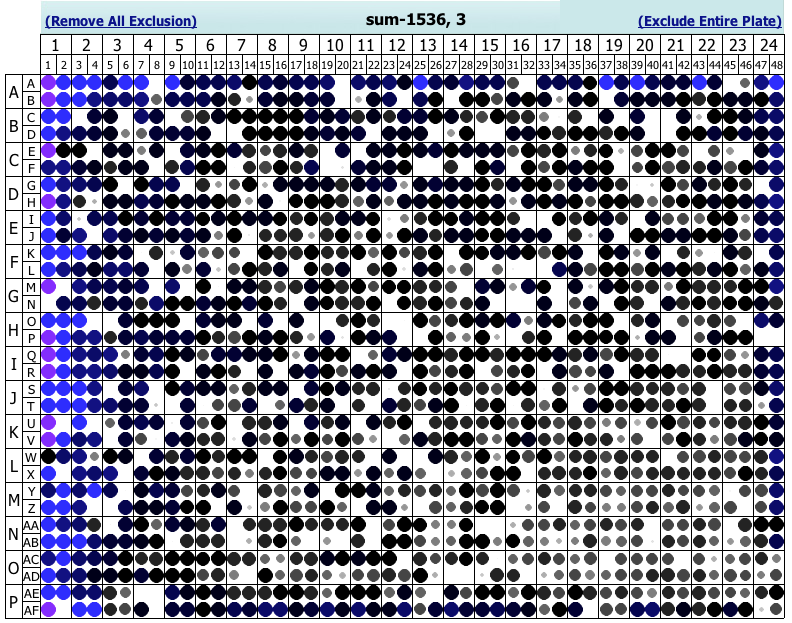

Supplement: Additional File 4 — Comparison of Measurement Modes (Dittmar et al, additional file 4.zip). This file contains data comparing CM Engine's three measurement modes to HT Colony Grid Analyzer [16] and Growth Detector [17]. This file contains data in several files: • Additional File 4 - Comparison of Measurement Modes.pdf: A summary of the results and notes on how the analysis was performed. • Cartoons: Cartoon representations of raw measurements generated in DR Engine (.png file formats). • CM Engine: the original images analyzed by CM Engine (.tif). • Growth Detector Data: Original images (.tif files) and results of running Growth Detector (.png files). • HT Colony Grid Data: Original images (.jpg) and results of running HT Colony Grid Analyzer (.dat and .png files). [file 1471-2105-11-353-S4.ZIP › Cartoons/1536/CM_Summation-1536,3.png]

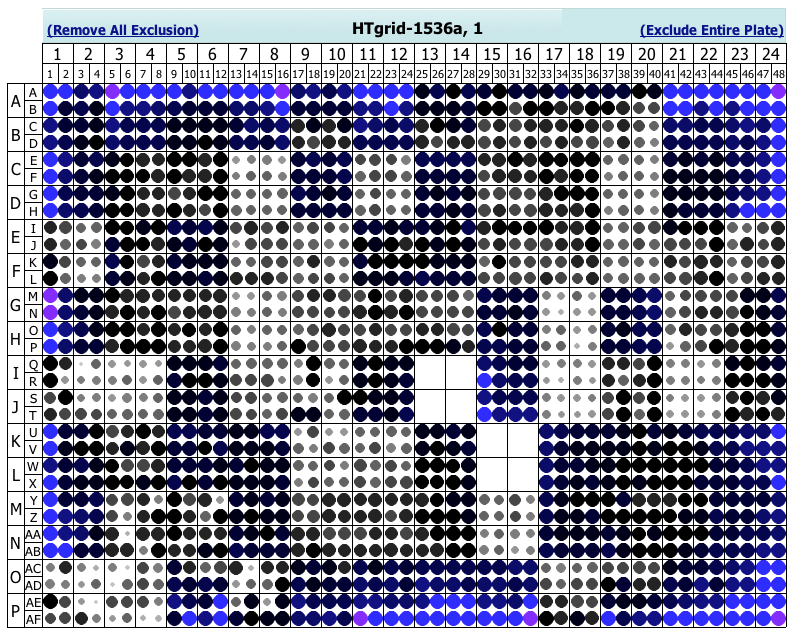

Supplement: Additional File 4 — Comparison of Measurement Modes (Dittmar et al, additional file 4.zip). This file contains data comparing CM Engine's three measurement modes to HT Colony Grid Analyzer [16] and Growth Detector [17]. This file contains data in several files: • Additional File 4 - Comparison of Measurement Modes.pdf: A summary of the results and notes on how the analysis was performed. • Cartoons: Cartoon representations of raw measurements generated in DR Engine (.png file formats). • CM Engine: the original images analyzed by CM Engine (.tif). • Growth Detector Data: Original images (.tif files) and results of running Growth Detector (.png files). • HT Colony Grid Data: Original images (.jpg) and results of running HT Colony Grid Analyzer (.dat and .png files). [file 1471-2105-11-353-S4.ZIP › Cartoons/1536/HT_ColonyGrid-1536,1.png]

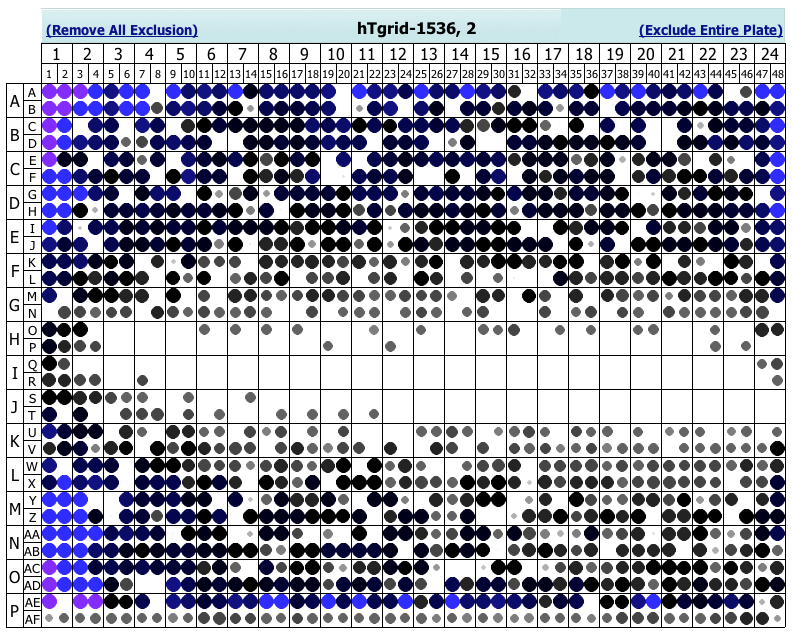

Supplement: Additional File 4 — Comparison of Measurement Modes (Dittmar et al, additional file 4.zip). This file contains data comparing CM Engine's three measurement modes to HT Colony Grid Analyzer [16] and Growth Detector [17]. This file contains data in several files: • Additional File 4 - Comparison of Measurement Modes.pdf: A summary of the results and notes on how the analysis was performed. • Cartoons: Cartoon representations of raw measurements generated in DR Engine (.png file formats). • CM Engine: the original images analyzed by CM Engine (.tif). • Growth Detector Data: Original images (.tif files) and results of running Growth Detector (.png files). • HT Colony Grid Data: Original images (.jpg) and results of running HT Colony Grid Analyzer (.dat and .png files). [file 1471-2105-11-353-S4.ZIP › Cartoons/1536/HT_ColonyGrid-1536,2.png]

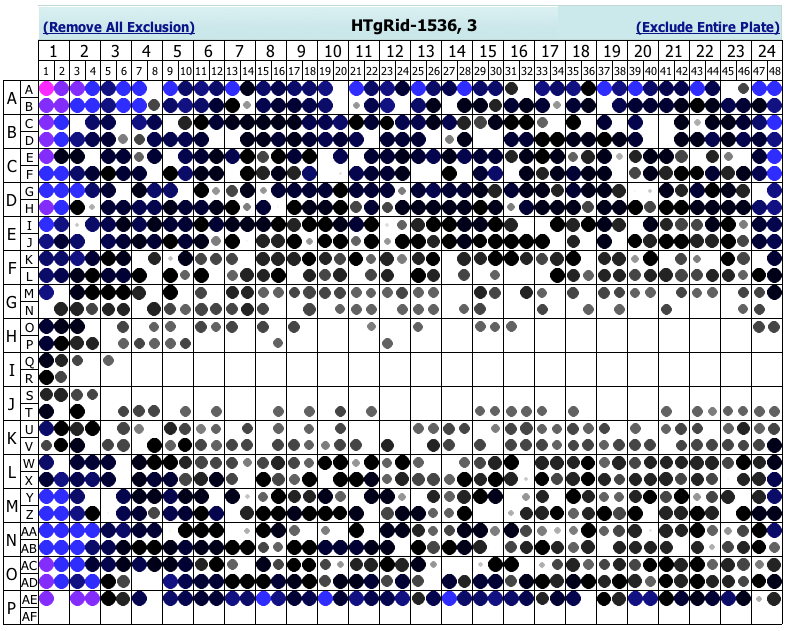

Supplement: Additional File 4 — Comparison of Measurement Modes (Dittmar et al, additional file 4.zip). This file contains data comparing CM Engine's three measurement modes to HT Colony Grid Analyzer [16] and Growth Detector [17]. This file contains data in several files: • Additional File 4 - Comparison of Measurement Modes.pdf: A summary of the results and notes on how the analysis was performed. • Cartoons: Cartoon representations of raw measurements generated in DR Engine (.png file formats). • CM Engine: the original images analyzed by CM Engine (.tif). • Growth Detector Data: Original images (.tif files) and results of running Growth Detector (.png files). • HT Colony Grid Data: Original images (.jpg) and results of running HT Colony Grid Analyzer (.dat and .png files). [file 1471-2105-11-353-S4.ZIP › Cartoons/1536/HT_ColonyGrid-1536,3.png]

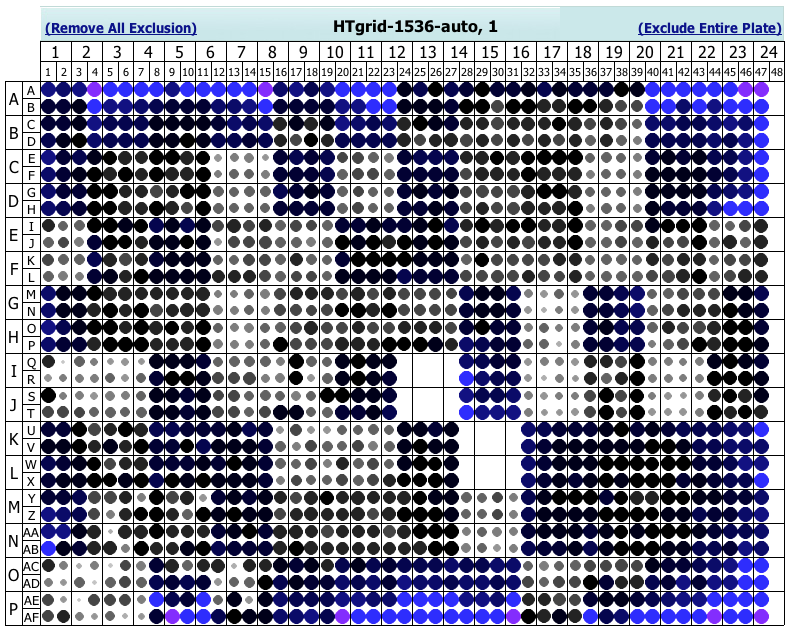

Supplement: Additional File 4 — Comparison of Measurement Modes (Dittmar et al, additional file 4.zip). This file contains data comparing CM Engine's three measurement modes to HT Colony Grid Analyzer [16] and Growth Detector [17]. This file contains data in several files: • Additional File 4 - Comparison of Measurement Modes.pdf: A summary of the results and notes on how the analysis was performed. • Cartoons: Cartoon representations of raw measurements generated in DR Engine (.png file formats). • CM Engine: the original images analyzed by CM Engine (.tif). • Growth Detector Data: Original images (.tif files) and results of running Growth Detector (.png files). • HT Colony Grid Data: Original images (.jpg) and results of running HT Colony Grid Analyzer (.dat and .png files). [file 1471-2105-11-353-S4.ZIP › Cartoons/1536/HT_ColonyGrid-1536_auto,1.png]

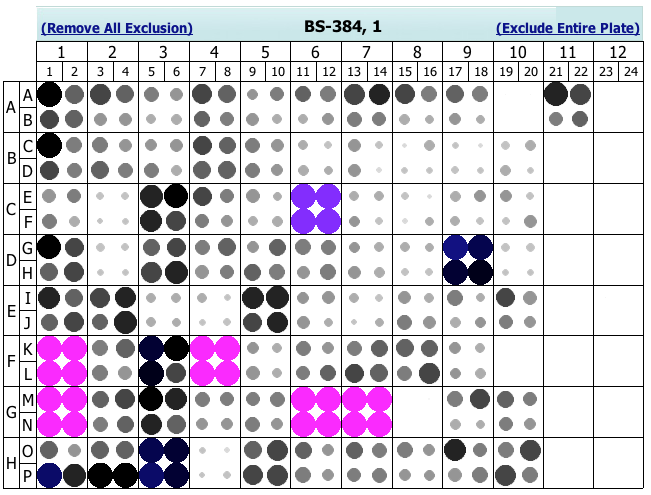

Supplement: Additional File 4 — Comparison of Measurement Modes (Dittmar et al, additional file 4.zip). This file contains data comparing CM Engine's three measurement modes to HT Colony Grid Analyzer [16] and Growth Detector [17]. This file contains data in several files: • Additional File 4 - Comparison of Measurement Modes.pdf: A summary of the results and notes on how the analysis was performed. • Cartoons: Cartoon representations of raw measurements generated in DR Engine (.png file formats). • CM Engine: the original images analyzed by CM Engine (.tif). • Growth Detector Data: Original images (.tif files) and results of running Growth Detector (.png files). • HT Colony Grid Data: Original images (.jpg) and results of running HT Colony Grid Analyzer (.dat and .png files). [file 1471-2105-11-353-S4.ZIP › Cartoons/384/CM_BackgroundSubtracted-384,1.PNG]

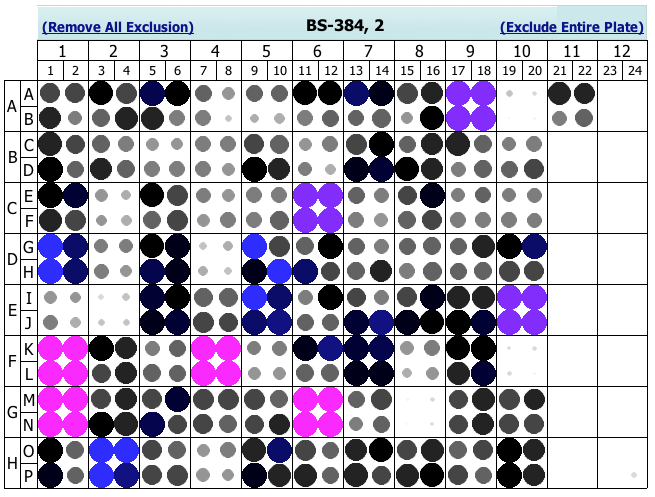

Supplement: Additional File 4 — Comparison of Measurement Modes (Dittmar et al, additional file 4.zip). This file contains data comparing CM Engine's three measurement modes to HT Colony Grid Analyzer [16] and Growth Detector [17]. This file contains data in several files: • Additional File 4 - Comparison of Measurement Modes.pdf: A summary of the results and notes on how the analysis was performed. • Cartoons: Cartoon representations of raw measurements generated in DR Engine (.png file formats). • CM Engine: the original images analyzed by CM Engine (.tif). • Growth Detector Data: Original images (.tif files) and results of running Growth Detector (.png files). • HT Colony Grid Data: Original images (.jpg) and results of running HT Colony Grid Analyzer (.dat and .png files). [file 1471-2105-11-353-S4.ZIP › Cartoons/384/CM_BackgroundSubtracted-384,2.PNG]

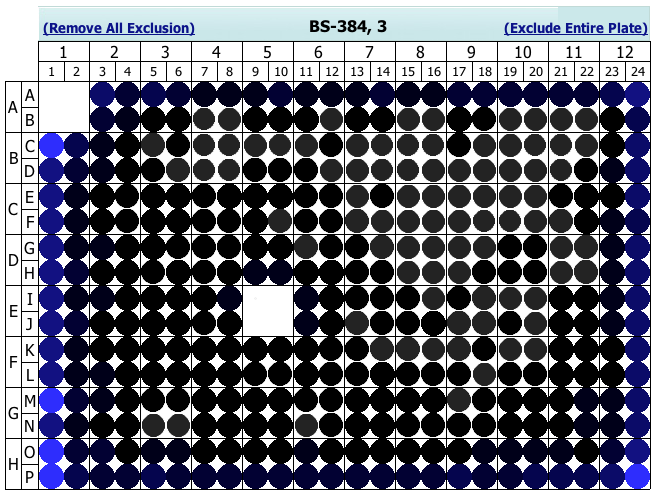

Supplement: Additional File 4 — Comparison of Measurement Modes (Dittmar et al, additional file 4.zip). This file contains data comparing CM Engine's three measurement modes to HT Colony Grid Analyzer [16] and Growth Detector [17]. This file contains data in several files: • Additional File 4 - Comparison of Measurement Modes.pdf: A summary of the results and notes on how the analysis was performed. • Cartoons: Cartoon representations of raw measurements generated in DR Engine (.png file formats). • CM Engine: the original images analyzed by CM Engine (.tif). • Growth Detector Data: Original images (.tif files) and results of running Growth Detector (.png files). • HT Colony Grid Data: Original images (.jpg) and results of running HT Colony Grid Analyzer (.dat and .png files). [file 1471-2105-11-353-S4.ZIP › Cartoons/384/CM_BackgroundSubtracted-384,3.PNG]

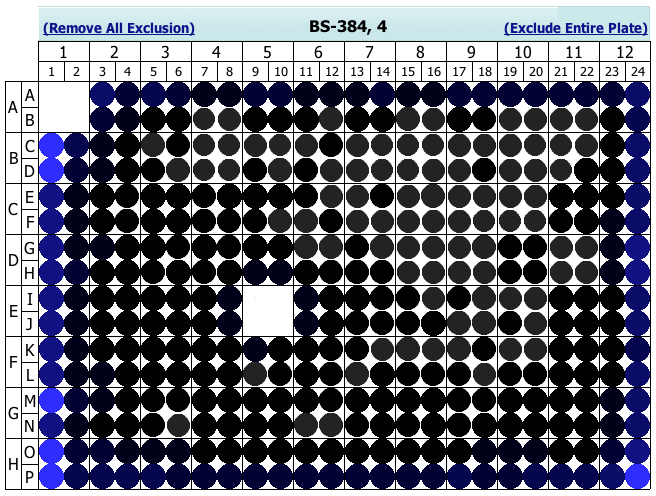

Supplement: Additional File 4 — Comparison of Measurement Modes (Dittmar et al, additional file 4.zip). This file contains data comparing CM Engine's three measurement modes to HT Colony Grid Analyzer [16] and Growth Detector [17]. This file contains data in several files: • Additional File 4 - Comparison of Measurement Modes.pdf: A summary of the results and notes on how the analysis was performed. • Cartoons: Cartoon representations of raw measurements generated in DR Engine (.png file formats). • CM Engine: the original images analyzed by CM Engine (.tif). • Growth Detector Data: Original images (.tif files) and results of running Growth Detector (.png files). • HT Colony Grid Data: Original images (.jpg) and results of running HT Colony Grid Analyzer (.dat and .png files). [file 1471-2105-11-353-S4.ZIP › Cartoons/384/CM_BackgroundSubtracted-384,4.PNG]

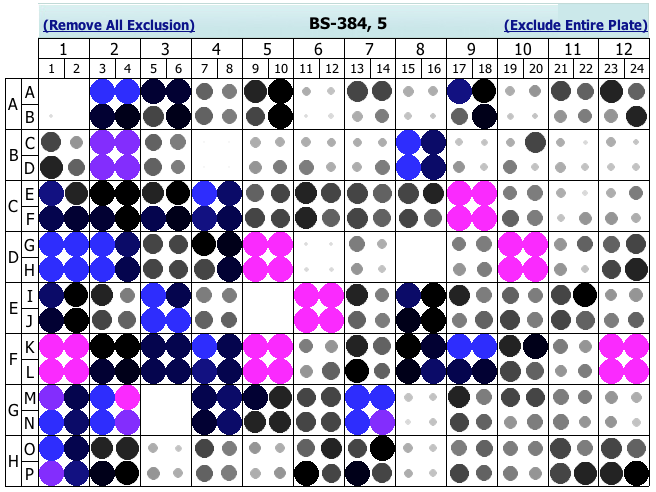

Supplement: Additional File 4 — Comparison of Measurement Modes (Dittmar et al, additional file 4.zip). This file contains data comparing CM Engine's three measurement modes to HT Colony Grid Analyzer [16] and Growth Detector [17]. This file contains data in several files: • Additional File 4 - Comparison of Measurement Modes.pdf: A summary of the results and notes on how the analysis was performed. • Cartoons: Cartoon representations of raw measurements generated in DR Engine (.png file formats). • CM Engine: the original images analyzed by CM Engine (.tif). • Growth Detector Data: Original images (.tif files) and results of running Growth Detector (.png files). • HT Colony Grid Data: Original images (.jpg) and results of running HT Colony Grid Analyzer (.dat and .png files). [file 1471-2105-11-353-S4.ZIP › Cartoons/384/CM_BackgroundSubtracted-384,5.PNG]

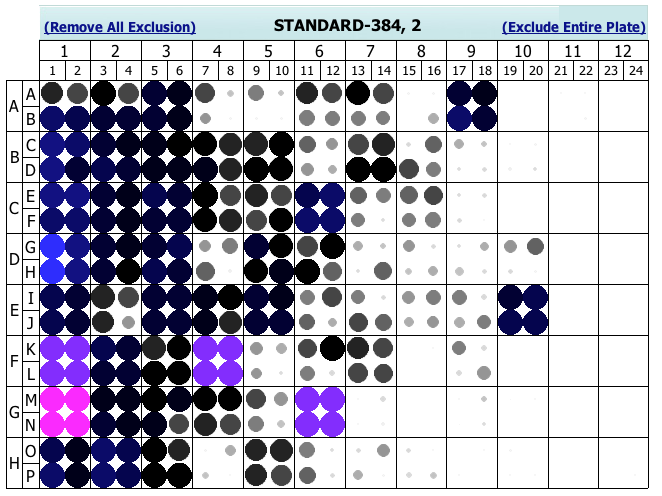

Supplement: Additional File 4 — Comparison of Measurement Modes (Dittmar et al, additional file 4.zip). This file contains data comparing CM Engine's three measurement modes to HT Colony Grid Analyzer [16] and Growth Detector [17]. This file contains data in several files: • Additional File 4 - Comparison of Measurement Modes.pdf: A summary of the results and notes on how the analysis was performed. • Cartoons: Cartoon representations of raw measurements generated in DR Engine (.png file formats). • CM Engine: the original images analyzed by CM Engine (.tif). • Growth Detector Data: Original images (.tif files) and results of running Growth Detector (.png files). • HT Colony Grid Data: Original images (.jpg) and results of running HT Colony Grid Analyzer (.dat and .png files). [file 1471-2105-11-353-S4.ZIP › Cartoons/384/CM_Standard-384,2.PNG]

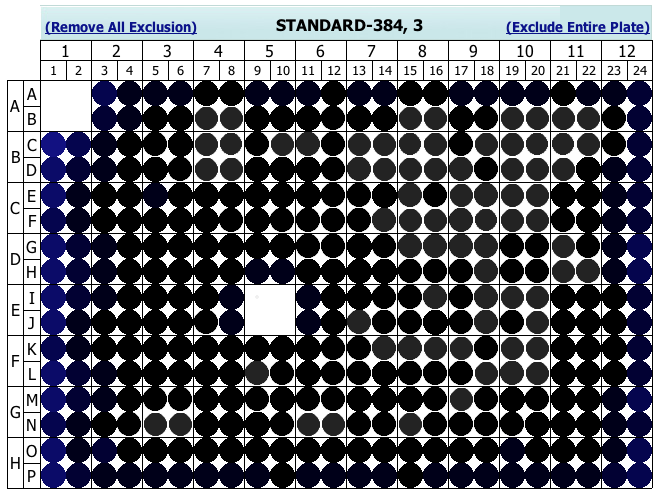

Supplement: Additional File 4 — Comparison of Measurement Modes (Dittmar et al, additional file 4.zip). This file contains data comparing CM Engine's three measurement modes to HT Colony Grid Analyzer [16] and Growth Detector [17]. This file contains data in several files: • Additional File 4 - Comparison of Measurement Modes.pdf: A summary of the results and notes on how the analysis was performed. • Cartoons: Cartoon representations of raw measurements generated in DR Engine (.png file formats). • CM Engine: the original images analyzed by CM Engine (.tif). • Growth Detector Data: Original images (.tif files) and results of running Growth Detector (.png files). • HT Colony Grid Data: Original images (.jpg) and results of running HT Colony Grid Analyzer (.dat and .png files). [file 1471-2105-11-353-S4.ZIP › Cartoons/384/CM_Standard-384,3.PNG]

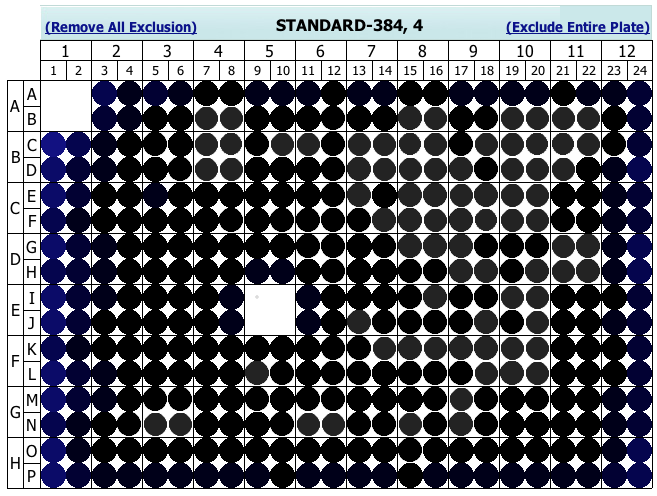

Supplement: Additional File 4 — Comparison of Measurement Modes (Dittmar et al, additional file 4.zip). This file contains data comparing CM Engine's three measurement modes to HT Colony Grid Analyzer [16] and Growth Detector [17]. This file contains data in several files: • Additional File 4 - Comparison of Measurement Modes.pdf: A summary of the results and notes on how the analysis was performed. • Cartoons: Cartoon representations of raw measurements generated in DR Engine (.png file formats). • CM Engine: the original images analyzed by CM Engine (.tif). • Growth Detector Data: Original images (.tif files) and results of running Growth Detector (.png files). • HT Colony Grid Data: Original images (.jpg) and results of running HT Colony Grid Analyzer (.dat and .png files). [file 1471-2105-11-353-S4.ZIP › Cartoons/384/CM_Standard-384,4.PNG]

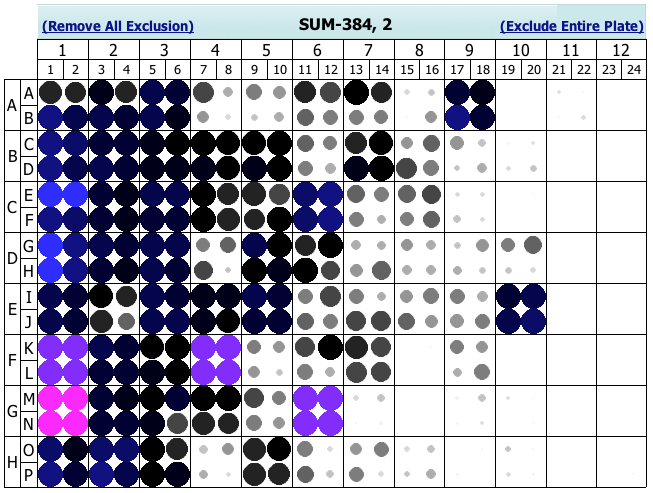

Supplement: Additional File 4 — Comparison of Measurement Modes (Dittmar et al, additional file 4.zip). This file contains data comparing CM Engine's three measurement modes to HT Colony Grid Analyzer [16] and Growth Detector [17]. This file contains data in several files: • Additional File 4 - Comparison of Measurement Modes.pdf: A summary of the results and notes on how the analysis was performed. • Cartoons: Cartoon representations of raw measurements generated in DR Engine (.png file formats). • CM Engine: the original images analyzed by CM Engine (.tif). • Growth Detector Data: Original images (.tif files) and results of running Growth Detector (.png files). • HT Colony Grid Data: Original images (.jpg) and results of running HT Colony Grid Analyzer (.dat and .png files). [file 1471-2105-11-353-S4.ZIP › Cartoons/384/CM_Summation-384,2.PNG]

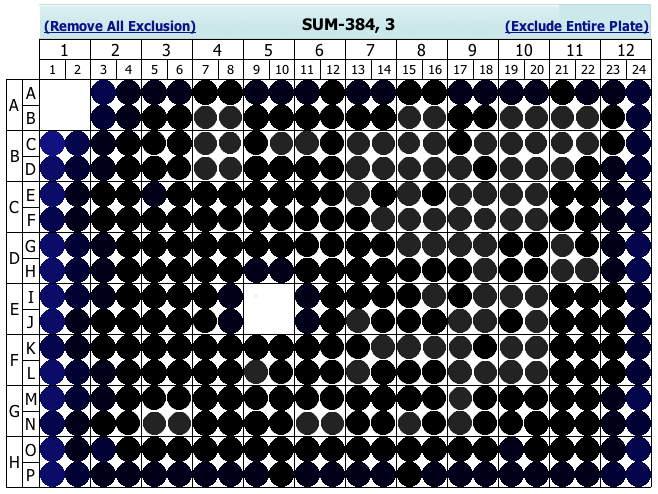

Supplement: Additional File 4 — Comparison of Measurement Modes (Dittmar et al, additional file 4.zip). This file contains data comparing CM Engine's three measurement modes to HT Colony Grid Analyzer [16] and Growth Detector [17]. This file contains data in several files: • Additional File 4 - Comparison of Measurement Modes.pdf: A summary of the results and notes on how the analysis was performed. • Cartoons: Cartoon representations of raw measurements generated in DR Engine (.png file formats). • CM Engine: the original images analyzed by CM Engine (.tif). • Growth Detector Data: Original images (.tif files) and results of running Growth Detector (.png files). • HT Colony Grid Data: Original images (.jpg) and results of running HT Colony Grid Analyzer (.dat and .png files). [file 1471-2105-11-353-S4.ZIP › Cartoons/384/CM_Summation-384,3.PNG]

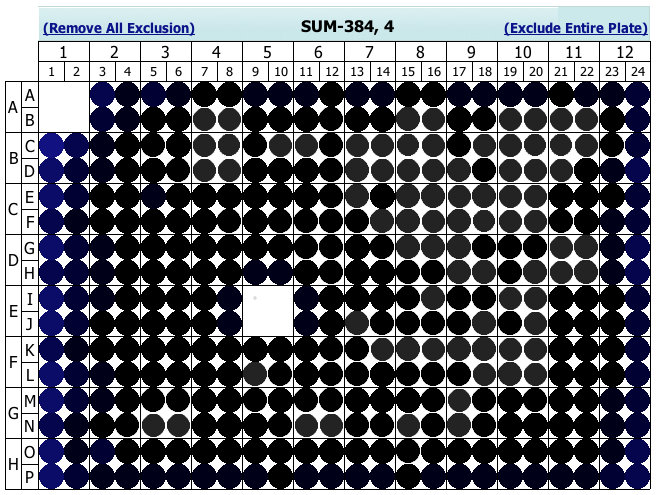

Supplement: Additional File 4 — Comparison of Measurement Modes (Dittmar et al, additional file 4.zip). This file contains data comparing CM Engine's three measurement modes to HT Colony Grid Analyzer [16] and Growth Detector [17]. This file contains data in several files: • Additional File 4 - Comparison of Measurement Modes.pdf: A summary of the results and notes on how the analysis was performed. • Cartoons: Cartoon representations of raw measurements generated in DR Engine (.png file formats). • CM Engine: the original images analyzed by CM Engine (.tif). • Growth Detector Data: Original images (.tif files) and results of running Growth Detector (.png files). • HT Colony Grid Data: Original images (.jpg) and results of running HT Colony Grid Analyzer (.dat and .png files). [file 1471-2105-11-353-S4.ZIP › Cartoons/384/CM_Summation-384,4.PNG]

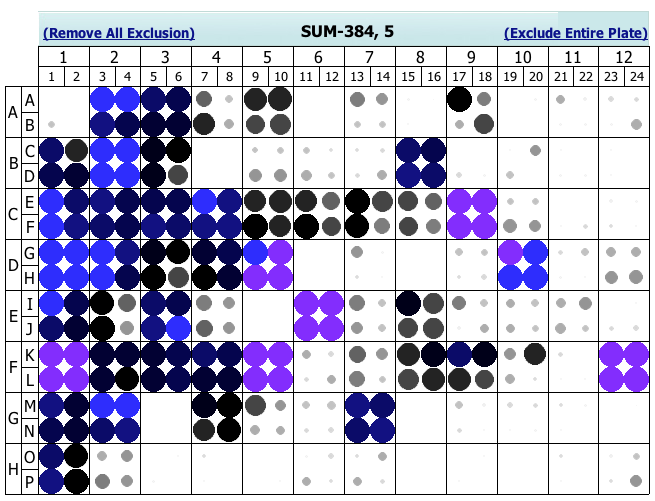

Supplement: Additional File 4 — Comparison of Measurement Modes (Dittmar et al, additional file 4.zip). This file contains data comparing CM Engine's three measurement modes to HT Colony Grid Analyzer [16] and Growth Detector [17]. This file contains data in several files: • Additional File 4 - Comparison of Measurement Modes.pdf: A summary of the results and notes on how the analysis was performed. • Cartoons: Cartoon representations of raw measurements generated in DR Engine (.png file formats). • CM Engine: the original images analyzed by CM Engine (.tif). • Growth Detector Data: Original images (.tif files) and results of running Growth Detector (.png files). • HT Colony Grid Data: Original images (.jpg) and results of running HT Colony Grid Analyzer (.dat and .png files). [file 1471-2105-11-353-S4.ZIP › Cartoons/384/CM_Summation-384,5.PNG]

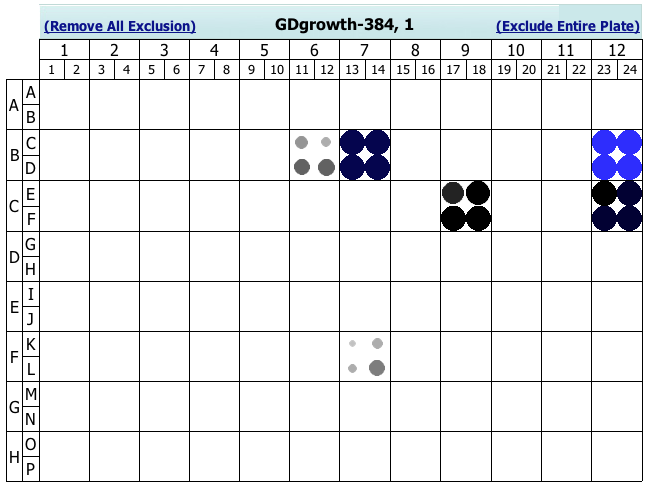

Supplement: Additional File 4 — Comparison of Measurement Modes (Dittmar et al, additional file 4.zip). This file contains data comparing CM Engine's three measurement modes to HT Colony Grid Analyzer [16] and Growth Detector [17]. This file contains data in several files: • Additional File 4 - Comparison of Measurement Modes.pdf: A summary of the results and notes on how the analysis was performed. • Cartoons: Cartoon representations of raw measurements generated in DR Engine (.png file formats). • CM Engine: the original images analyzed by CM Engine (.tif). • Growth Detector Data: Original images (.tif files) and results of running Growth Detector (.png files). • HT Colony Grid Data: Original images (.jpg) and results of running HT Colony Grid Analyzer (.dat and .png files). [file 1471-2105-11-353-S4.ZIP › Cartoons/384/GrowthDetector-384,1.PNG]

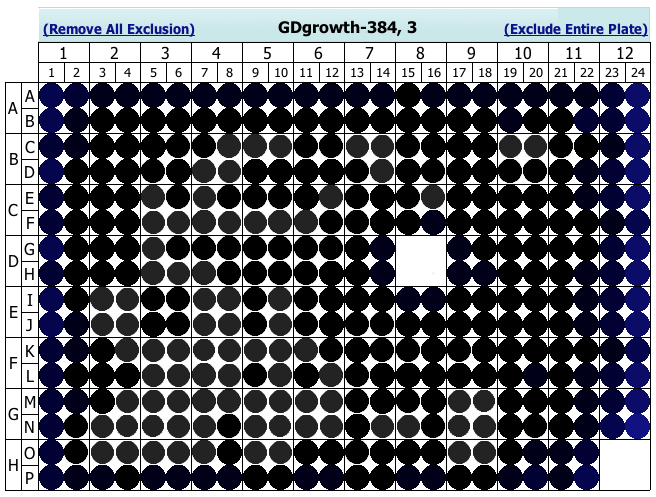

Supplement: Additional File 4 — Comparison of Measurement Modes (Dittmar et al, additional file 4.zip). This file contains data comparing CM Engine's three measurement modes to HT Colony Grid Analyzer [16] and Growth Detector [17]. This file contains data in several files: • Additional File 4 - Comparison of Measurement Modes.pdf: A summary of the results and notes on how the analysis was performed. • Cartoons: Cartoon representations of raw measurements generated in DR Engine (.png file formats). • CM Engine: the original images analyzed by CM Engine (.tif). • Growth Detector Data: Original images (.tif files) and results of running Growth Detector (.png files). • HT Colony Grid Data: Original images (.jpg) and results of running HT Colony Grid Analyzer (.dat and .png files). [file 1471-2105-11-353-S4.ZIP › Cartoons/384/GrowthDetector-384,3.PNG]

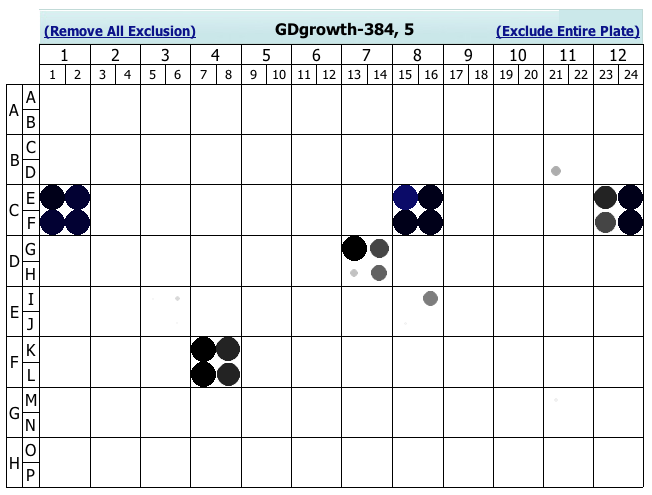

Supplement: Additional File 4 — Comparison of Measurement Modes (Dittmar et al, additional file 4.zip). This file contains data comparing CM Engine's three measurement modes to HT Colony Grid Analyzer [16] and Growth Detector [17]. This file contains data in several files: • Additional File 4 - Comparison of Measurement Modes.pdf: A summary of the results and notes on how the analysis was performed. • Cartoons: Cartoon representations of raw measurements generated in DR Engine (.png file formats). • CM Engine: the original images analyzed by CM Engine (.tif). • Growth Detector Data: Original images (.tif files) and results of running Growth Detector (.png files). • HT Colony Grid Data: Original images (.jpg) and results of running HT Colony Grid Analyzer (.dat and .png files). [file 1471-2105-11-353-S4.ZIP › Cartoons/384/GrowthDetector-384,5.PNG]

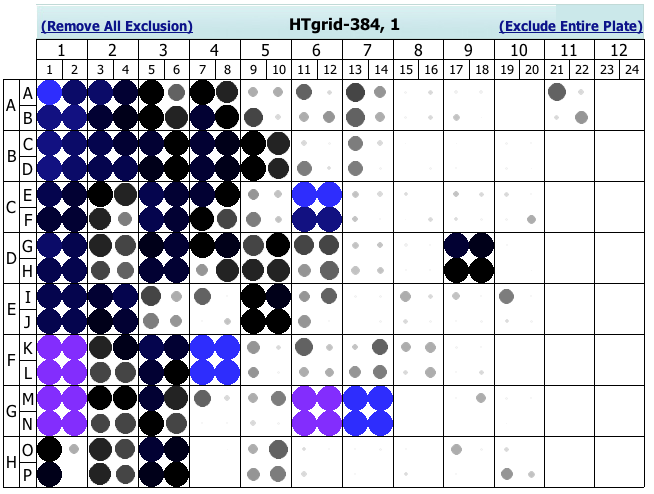

Supplement: Additional File 4 — Comparison of Measurement Modes (Dittmar et al, additional file 4.zip). This file contains data comparing CM Engine's three measurement modes to HT Colony Grid Analyzer [16] and Growth Detector [17]. This file contains data in several files: • Additional File 4 - Comparison of Measurement Modes.pdf: A summary of the results and notes on how the analysis was performed. • Cartoons: Cartoon representations of raw measurements generated in DR Engine (.png file formats). • CM Engine: the original images analyzed by CM Engine (.tif). • Growth Detector Data: Original images (.tif files) and results of running Growth Detector (.png files). • HT Colony Grid Data: Original images (.jpg) and results of running HT Colony Grid Analyzer (.dat and .png files). [file 1471-2105-11-353-S4.ZIP › Cartoons/384/HT_ColonyGrid-384,1.PNG]

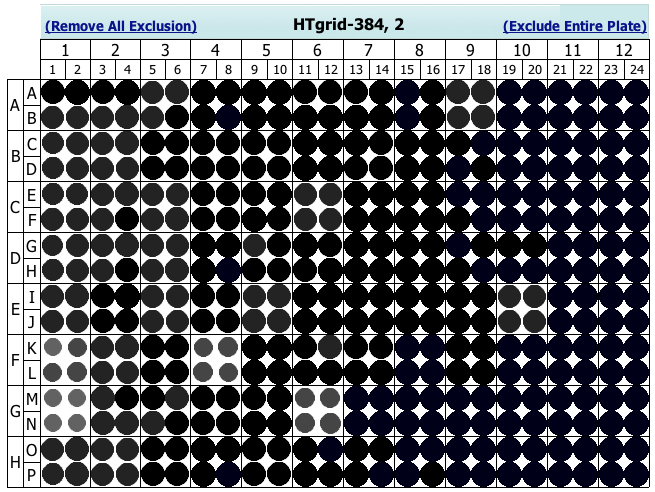

Supplement: Additional File 4 — Comparison of Measurement Modes (Dittmar et al, additional file 4.zip). This file contains data comparing CM Engine's three measurement modes to HT Colony Grid Analyzer [16] and Growth Detector [17]. This file contains data in several files: • Additional File 4 - Comparison of Measurement Modes.pdf: A summary of the results and notes on how the analysis was performed. • Cartoons: Cartoon representations of raw measurements generated in DR Engine (.png file formats). • CM Engine: the original images analyzed by CM Engine (.tif). • Growth Detector Data: Original images (.tif files) and results of running Growth Detector (.png files). • HT Colony Grid Data: Original images (.jpg) and results of running HT Colony Grid Analyzer (.dat and .png files). [file 1471-2105-11-353-S4.ZIP › Cartoons/384/HT_ColonyGrid-384,2.PNG]

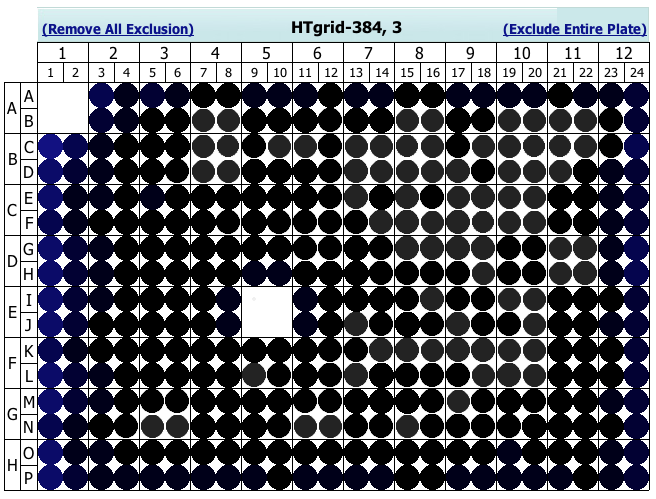

Supplement: Additional File 4 — Comparison of Measurement Modes (Dittmar et al, additional file 4.zip). This file contains data comparing CM Engine's three measurement modes to HT Colony Grid Analyzer [16] and Growth Detector [17]. This file contains data in several files: • Additional File 4 - Comparison of Measurement Modes.pdf: A summary of the results and notes on how the analysis was performed. • Cartoons: Cartoon representations of raw measurements generated in DR Engine (.png file formats). • CM Engine: the original images analyzed by CM Engine (.tif). • Growth Detector Data: Original images (.tif files) and results of running Growth Detector (.png files). • HT Colony Grid Data: Original images (.jpg) and results of running HT Colony Grid Analyzer (.dat and .png files). [file 1471-2105-11-353-S4.ZIP › Cartoons/384/HT_ColonyGrid-384,3.PNG]

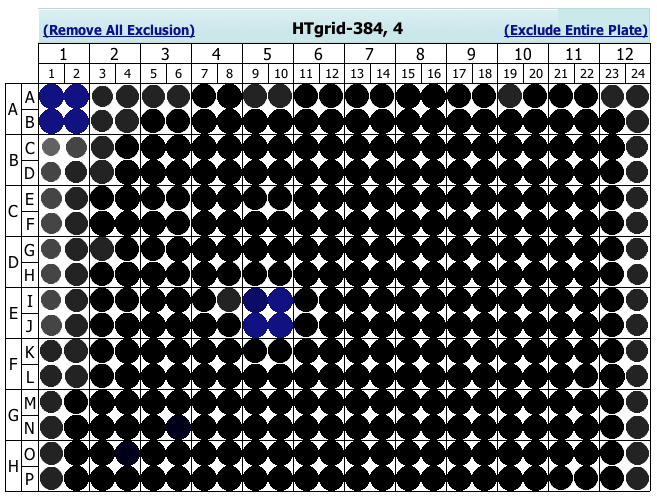

Supplement: Additional File 4 — Comparison of Measurement Modes (Dittmar et al, additional file 4.zip). This file contains data comparing CM Engine's three measurement modes to HT Colony Grid Analyzer [16] and Growth Detector [17]. This file contains data in several files: • Additional File 4 - Comparison of Measurement Modes.pdf: A summary of the results and notes on how the analysis was performed. • Cartoons: Cartoon representations of raw measurements generated in DR Engine (.png file formats). • CM Engine: the original images analyzed by CM Engine (.tif). • Growth Detector Data: Original images (.tif files) and results of running Growth Detector (.png files). • HT Colony Grid Data: Original images (.jpg) and results of running HT Colony Grid Analyzer (.dat and .png files). [file 1471-2105-11-353-S4.ZIP › Cartoons/384/HT_ColonyGrid-384,4.PNG]

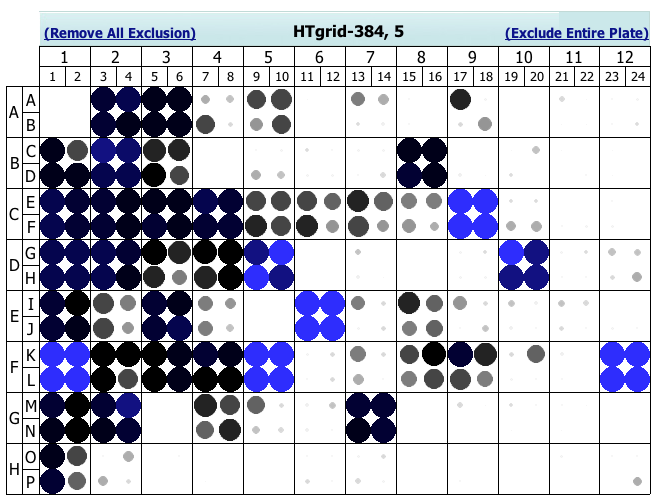

Supplement: Additional File 4 — Comparison of Measurement Modes (Dittmar et al, additional file 4.zip). This file contains data comparing CM Engine's three measurement modes to HT Colony Grid Analyzer [16] and Growth Detector [17]. This file contains data in several files: • Additional File 4 - Comparison of Measurement Modes.pdf: A summary of the results and notes on how the analysis was performed. • Cartoons: Cartoon representations of raw measurements generated in DR Engine (.png file formats). • CM Engine: the original images analyzed by CM Engine (.tif). • Growth Detector Data: Original images (.tif files) and results of running Growth Detector (.png files). • HT Colony Grid Data: Original images (.jpg) and results of running HT Colony Grid Analyzer (.dat and .png files). [file 1471-2105-11-353-S4.ZIP › Cartoons/384/HT_ColonyGrid-384,5.PNG]

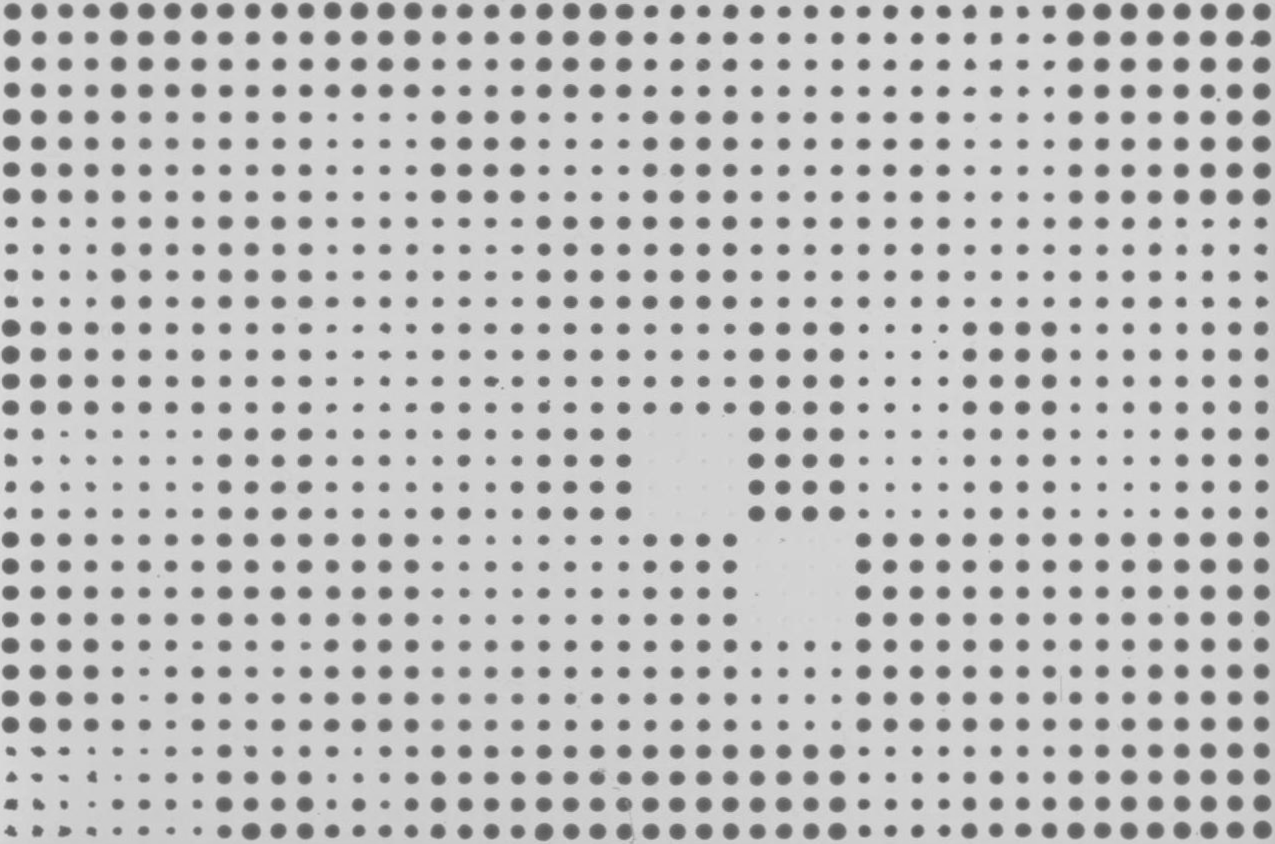

Supplement: Additional File 4 — Comparison of Measurement Modes (Dittmar et al, additional file 4.zip). This file contains data comparing CM Engine's three measurement modes to HT Colony Grid Analyzer [16] and Growth Detector [17]. This file contains data in several files: • Additional File 4 - Comparison of Measurement Modes.pdf: A summary of the results and notes on how the analysis was performed. • Cartoons: Cartoon representations of raw measurements generated in DR Engine (.png file formats). • CM Engine: the original images analyzed by CM Engine (.tif). • Growth Detector Data: Original images (.tif files) and results of running Growth Detector (.png files). • HT Colony Grid Data: Original images (.jpg) and results of running HT Colony Grid Analyzer (.dat and .png files). [file 1471-2105-11-353-S4.ZIP › CM Engine/1536/1536,1.tif]

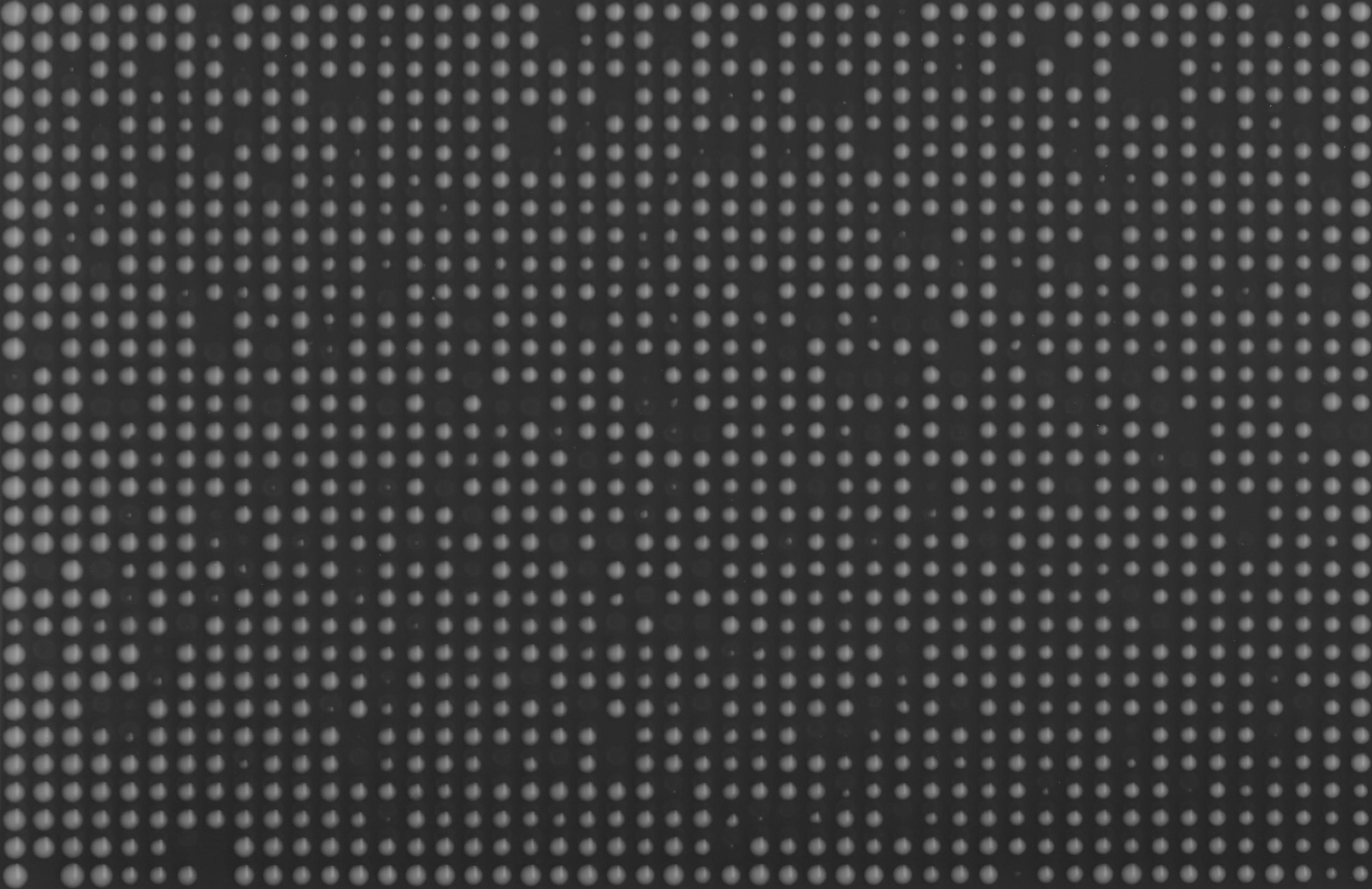

Supplement: Additional File 4 — Comparison of Measurement Modes (Dittmar et al, additional file 4.zip). This file contains data comparing CM Engine's three measurement modes to HT Colony Grid Analyzer [16] and Growth Detector [17]. This file contains data in several files: • Additional File 4 - Comparison of Measurement Modes.pdf: A summary of the results and notes on how the analysis was performed. • Cartoons: Cartoon representations of raw measurements generated in DR Engine (.png file formats). • CM Engine: the original images analyzed by CM Engine (.tif). • Growth Detector Data: Original images (.tif files) and results of running Growth Detector (.png files). • HT Colony Grid Data: Original images (.jpg) and results of running HT Colony Grid Analyzer (.dat and .png files). [file 1471-2105-11-353-S4.ZIP › CM Engine/1536/1536,2.tif]

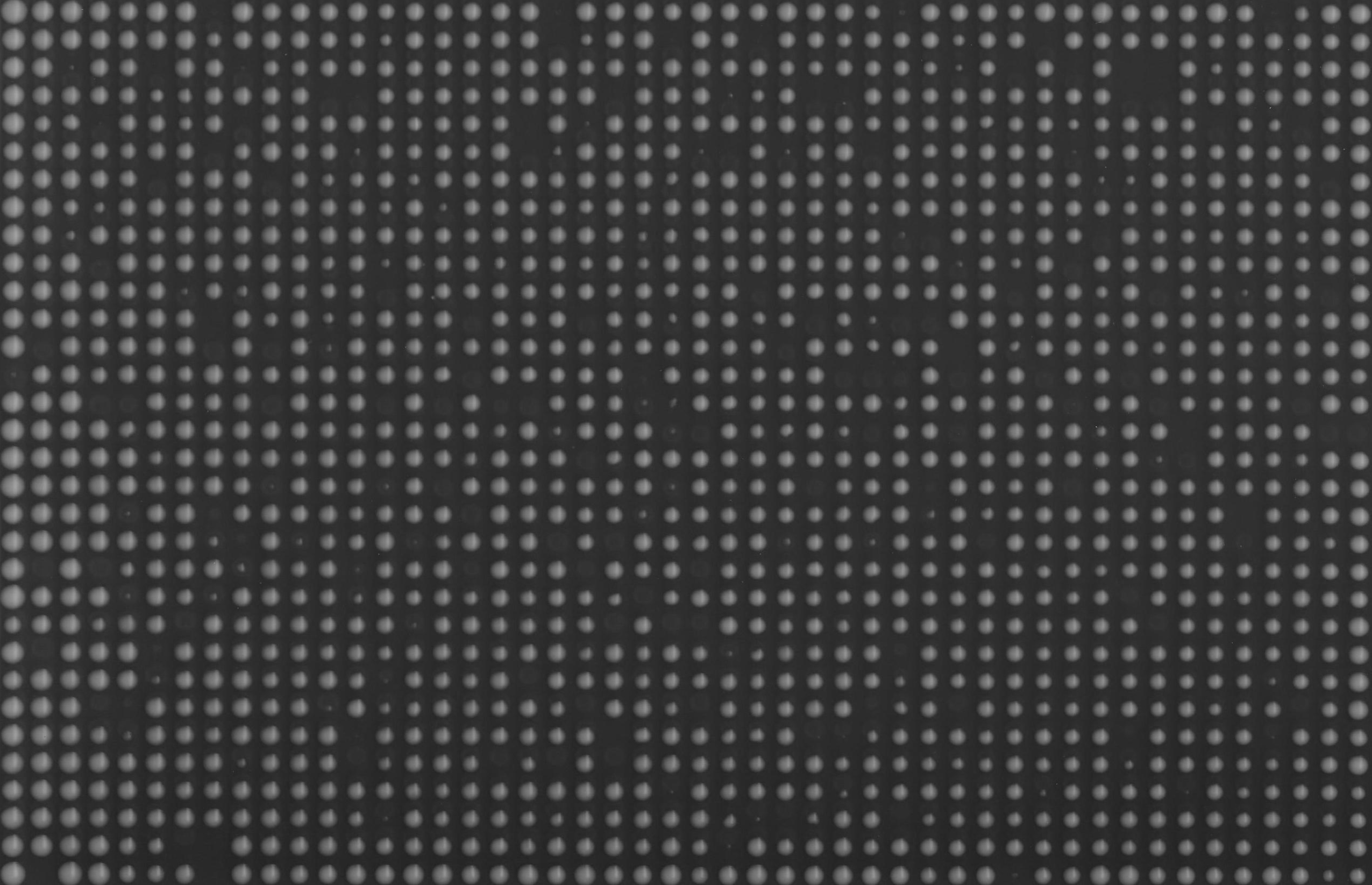

Supplement: Additional File 4 — Comparison of Measurement Modes (Dittmar et al, additional file 4.zip). This file contains data comparing CM Engine's three measurement modes to HT Colony Grid Analyzer [16] and Growth Detector [17]. This file contains data in several files: • Additional File 4 - Comparison of Measurement Modes.pdf: A summary of the results and notes on how the analysis was performed. • Cartoons: Cartoon representations of raw measurements generated in DR Engine (.png file formats). • CM Engine: the original images analyzed by CM Engine (.tif). • Growth Detector Data: Original images (.tif files) and results of running Growth Detector (.png files). • HT Colony Grid Data: Original images (.jpg) and results of running HT Colony Grid Analyzer (.dat and .png files). [file 1471-2105-11-353-S4.ZIP › CM Engine/1536/1536,3.tif]

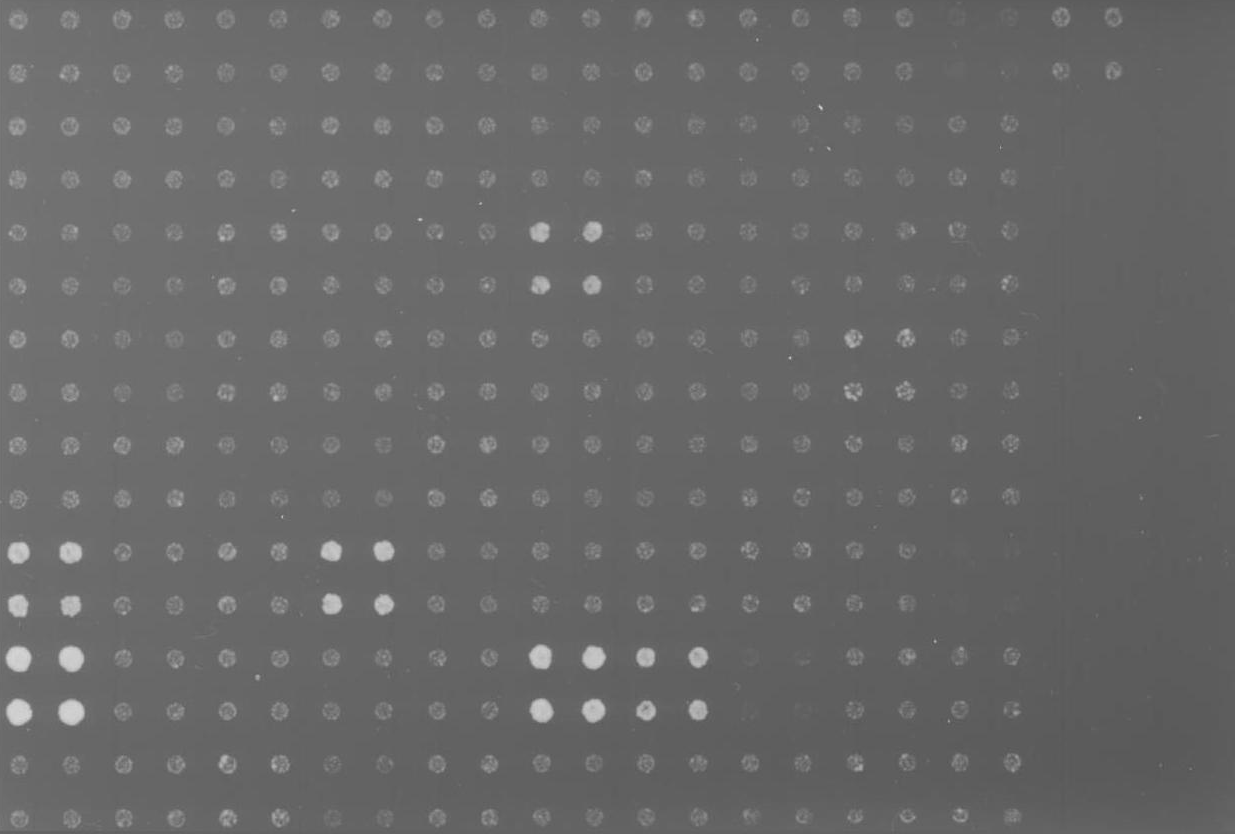

Supplement: Additional File 4 — Comparison of Measurement Modes (Dittmar et al, additional file 4.zip). This file contains data comparing CM Engine's three measurement modes to HT Colony Grid Analyzer [16] and Growth Detector [17]. This file contains data in several files: • Additional File 4 - Comparison of Measurement Modes.pdf: A summary of the results and notes on how the analysis was performed. • Cartoons: Cartoon representations of raw measurements generated in DR Engine (.png file formats). • CM Engine: the original images analyzed by CM Engine (.tif). • Growth Detector Data: Original images (.tif files) and results of running Growth Detector (.png files). • HT Colony Grid Data: Original images (.jpg) and results of running HT Colony Grid Analyzer (.dat and .png files). [file 1471-2105-11-353-S4.ZIP › CM Engine/384/384-1.tif]

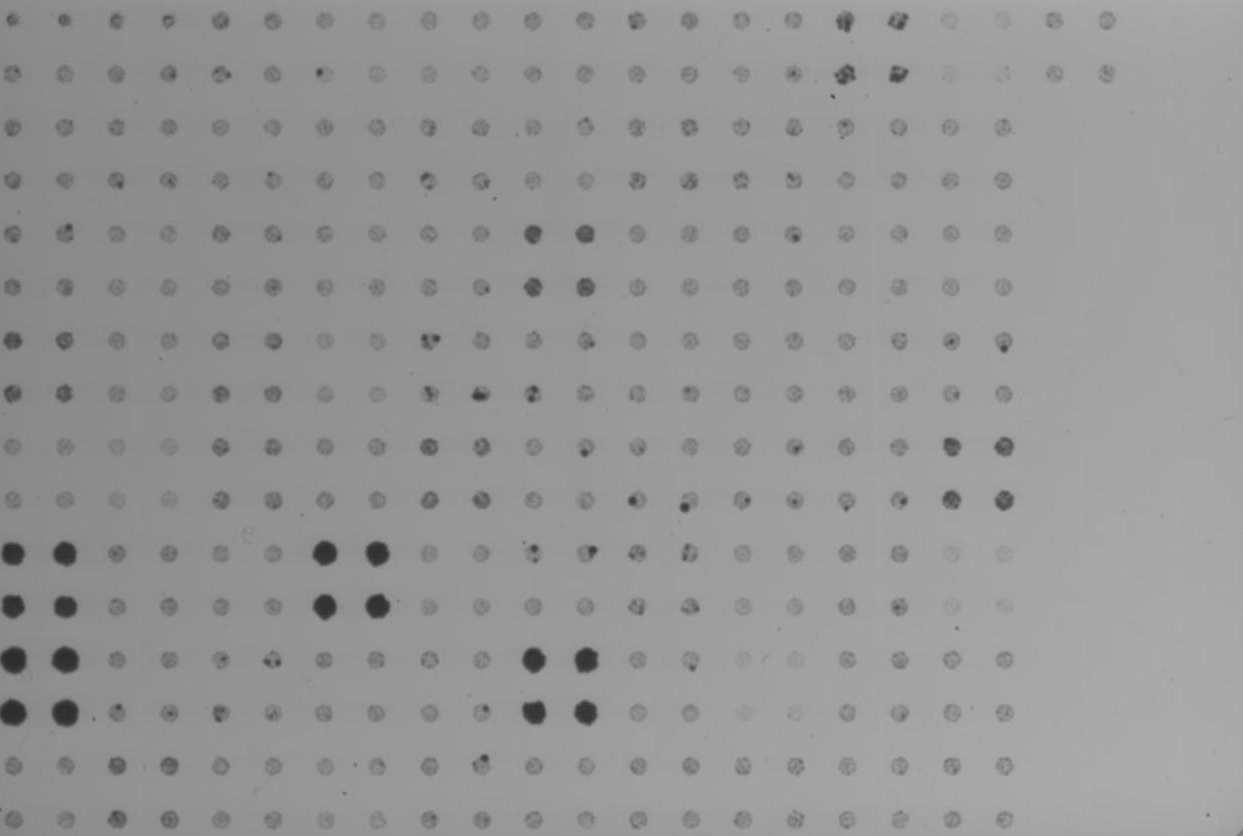

Supplement: Additional File 4 — Comparison of Measurement Modes (Dittmar et al, additional file 4.zip). This file contains data comparing CM Engine's three measurement modes to HT Colony Grid Analyzer [16] and Growth Detector [17]. This file contains data in several files: • Additional File 4 - Comparison of Measurement Modes.pdf: A summary of the results and notes on how the analysis was performed. • Cartoons: Cartoon representations of raw measurements generated in DR Engine (.png file formats). • CM Engine: the original images analyzed by CM Engine (.tif). • Growth Detector Data: Original images (.tif files) and results of running Growth Detector (.png files). • HT Colony Grid Data: Original images (.jpg) and results of running HT Colony Grid Analyzer (.dat and .png files). [file 1471-2105-11-353-S4.ZIP › CM Engine/384/384-2.tif]

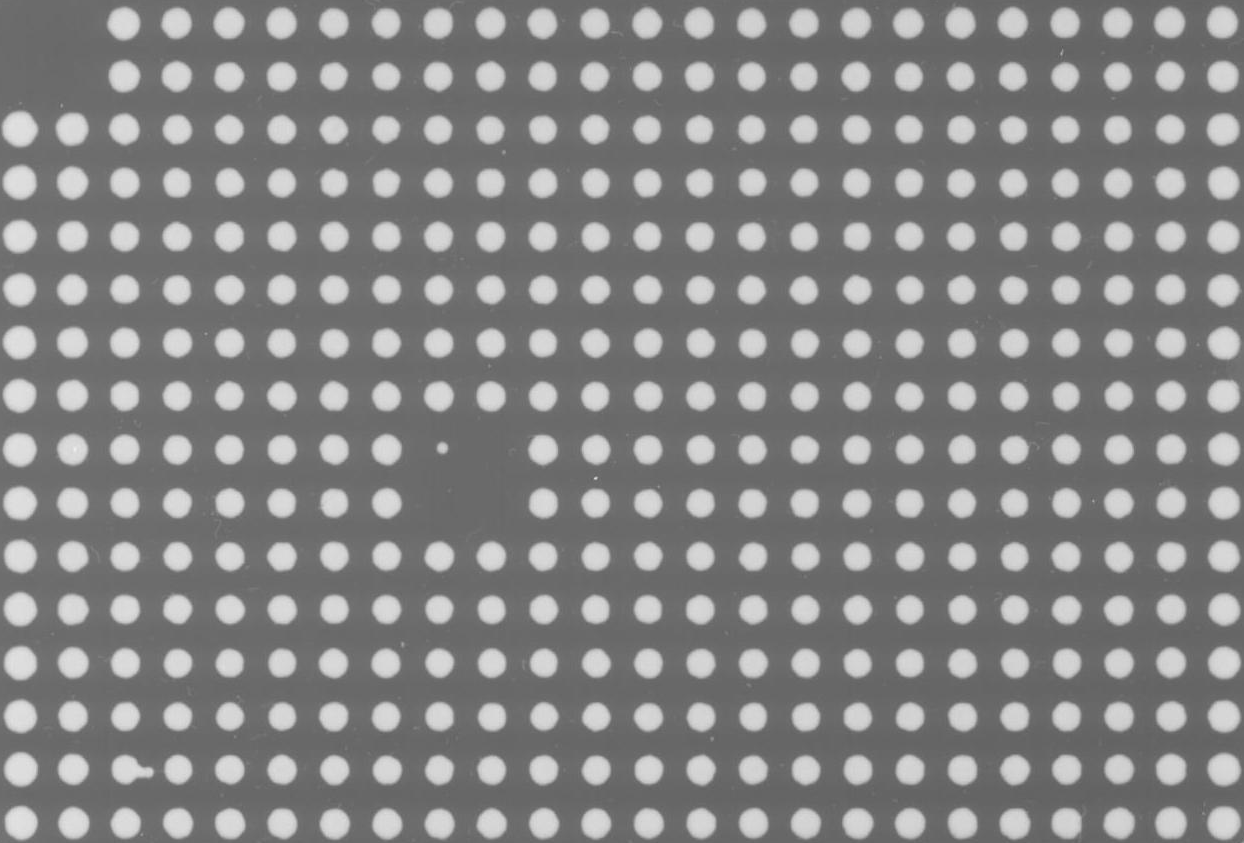

Supplement: Additional File 4 — Comparison of Measurement Modes (Dittmar et al, additional file 4.zip). This file contains data comparing CM Engine's three measurement modes to HT Colony Grid Analyzer [16] and Growth Detector [17]. This file contains data in several files: • Additional File 4 - Comparison of Measurement Modes.pdf: A summary of the results and notes on how the analysis was performed. • Cartoons: Cartoon representations of raw measurements generated in DR Engine (.png file formats). • CM Engine: the original images analyzed by CM Engine (.tif). • Growth Detector Data: Original images (.tif files) and results of running Growth Detector (.png files). • HT Colony Grid Data: Original images (.jpg) and results of running HT Colony Grid Analyzer (.dat and .png files). [file 1471-2105-11-353-S4.ZIP › CM Engine/384/384-3.tif]

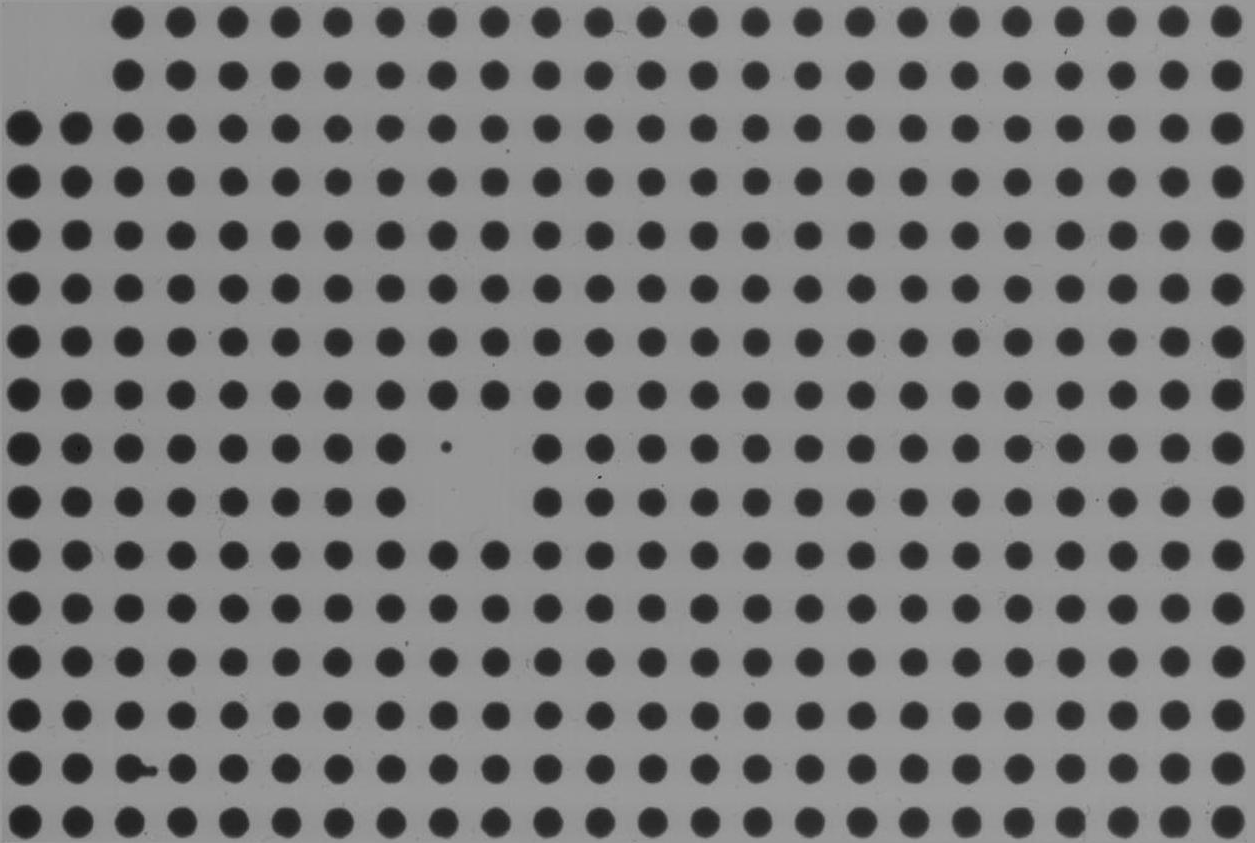

Supplement: Additional File 4 — Comparison of Measurement Modes (Dittmar et al, additional file 4.zip). This file contains data comparing CM Engine's three measurement modes to HT Colony Grid Analyzer [16] and Growth Detector [17]. This file contains data in several files: • Additional File 4 - Comparison of Measurement Modes.pdf: A summary of the results and notes on how the analysis was performed. • Cartoons: Cartoon representations of raw measurements generated in DR Engine (.png file formats). • CM Engine: the original images analyzed by CM Engine (.tif). • Growth Detector Data: Original images (.tif files) and results of running Growth Detector (.png files). • HT Colony Grid Data: Original images (.jpg) and results of running HT Colony Grid Analyzer (.dat and .png files). [file 1471-2105-11-353-S4.ZIP › CM Engine/384/384-4.tif]

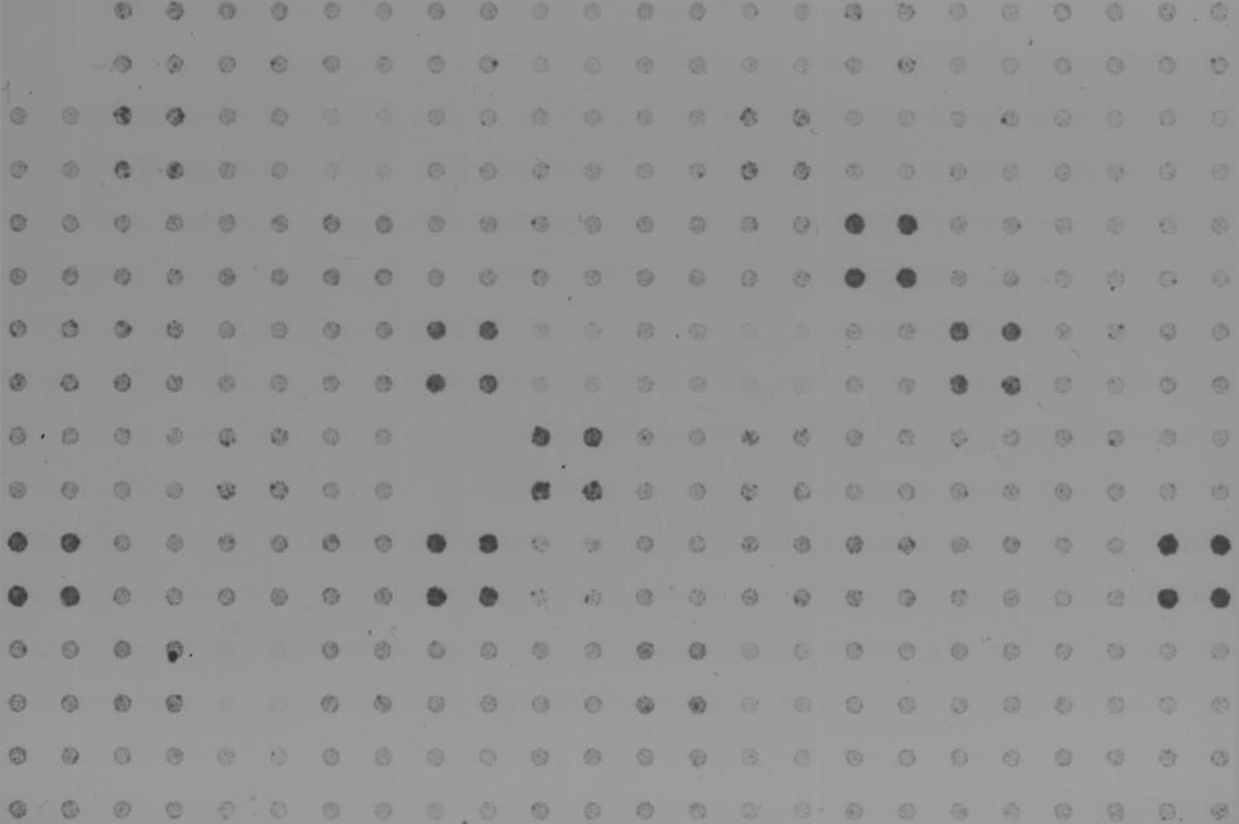

Supplement: Additional File 4 — Comparison of Measurement Modes (Dittmar et al, additional file 4.zip). This file contains data comparing CM Engine's three measurement modes to HT Colony Grid Analyzer [16] and Growth Detector [17]. This file contains data in several files: • Additional File 4 - Comparison of Measurement Modes.pdf: A summary of the results and notes on how the analysis was performed. • Cartoons: Cartoon representations of raw measurements generated in DR Engine (.png file formats). • CM Engine: the original images analyzed by CM Engine (.tif). • Growth Detector Data: Original images (.tif files) and results of running Growth Detector (.png files). • HT Colony Grid Data: Original images (.jpg) and results of running HT Colony Grid Analyzer (.dat and .png files). [file 1471-2105-11-353-S4.ZIP › CM Engine/384/384-5.tif]

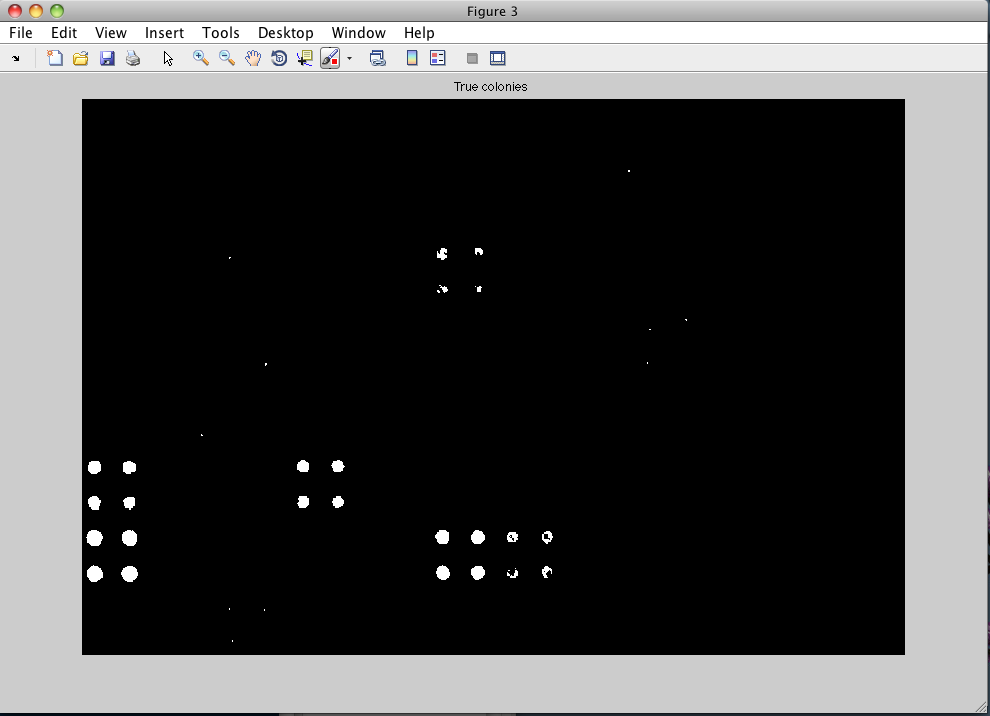

Supplement: Additional File 4 — Comparison of Measurement Modes (Dittmar et al, additional file 4.zip). This file contains data comparing CM Engine's three measurement modes to HT Colony Grid Analyzer [16] and Growth Detector [17]. This file contains data in several files: • Additional File 4 - Comparison of Measurement Modes.pdf: A summary of the results and notes on how the analysis was performed. • Cartoons: Cartoon representations of raw measurements generated in DR Engine (.png file formats). • CM Engine: the original images analyzed by CM Engine (.tif). • Growth Detector Data: Original images (.tif files) and results of running Growth Detector (.png files). • HT Colony Grid Data: Original images (.jpg) and results of running HT Colony Grid Analyzer (.dat and .png files). [file 1471-2105-11-353-S4.ZIP › Growth Detector Data/384-1 GrowthDetector 'true colonies'.PNG]

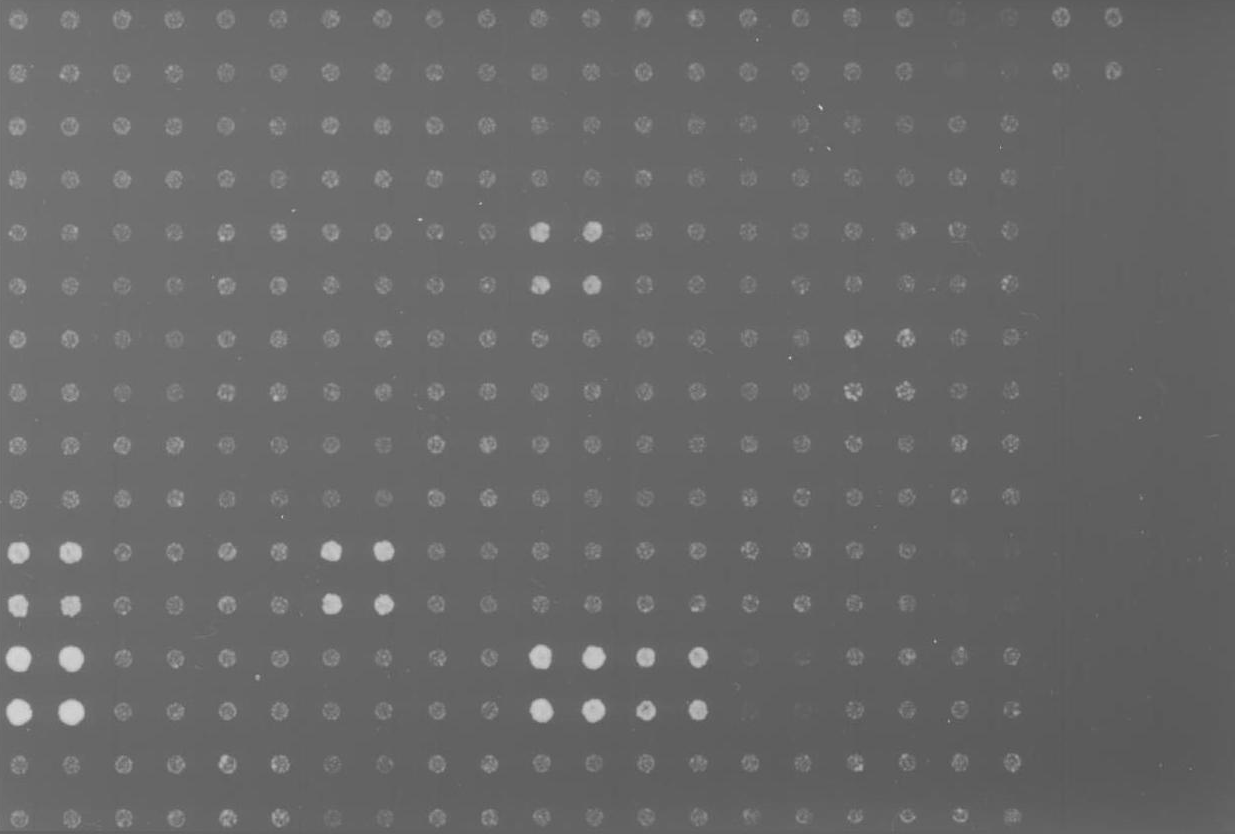

Supplement: Additional File 4 — Comparison of Measurement Modes (Dittmar et al, additional file 4.zip). This file contains data comparing CM Engine's three measurement modes to HT Colony Grid Analyzer [16] and Growth Detector [17]. This file contains data in several files: • Additional File 4 - Comparison of Measurement Modes.pdf: A summary of the results and notes on how the analysis was performed. • Cartoons: Cartoon representations of raw measurements generated in DR Engine (.png file formats). • CM Engine: the original images analyzed by CM Engine (.tif). • Growth Detector Data: Original images (.tif files) and results of running Growth Detector (.png files). • HT Colony Grid Data: Original images (.jpg) and results of running HT Colony Grid Analyzer (.dat and .png files). [file 1471-2105-11-353-S4.ZIP › Growth Detector Data/384-1.tif]

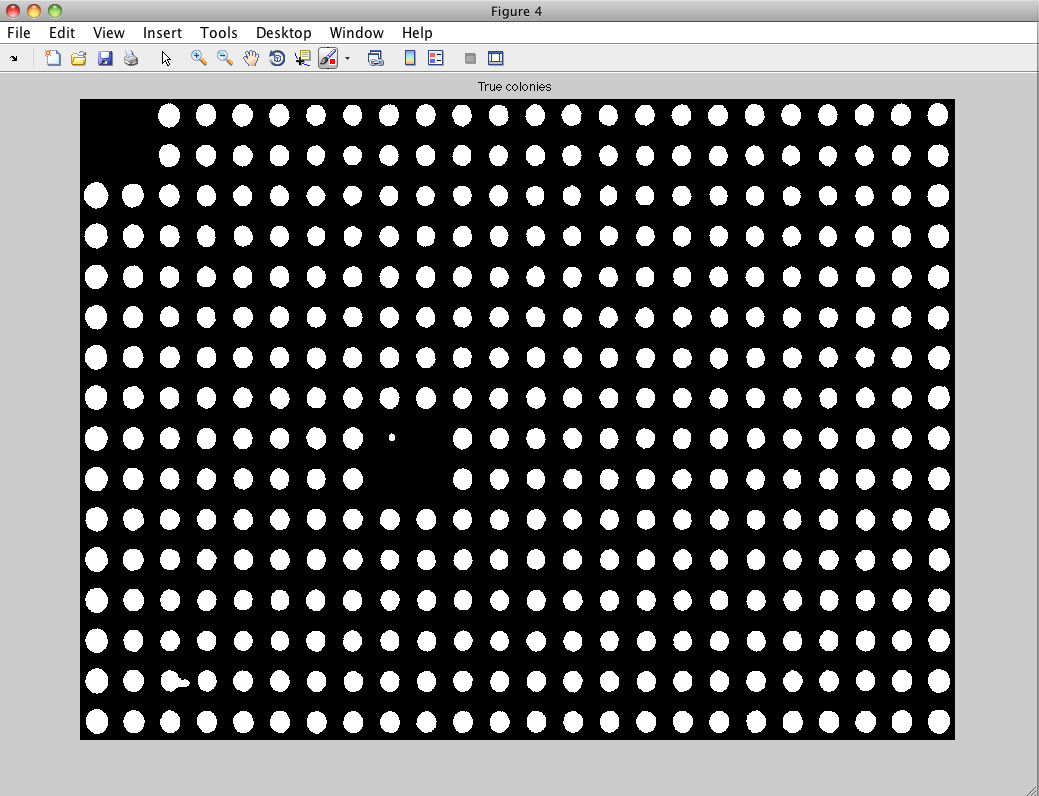

Supplement: Additional File 4 — Comparison of Measurement Modes (Dittmar et al, additional file 4.zip). This file contains data comparing CM Engine's three measurement modes to HT Colony Grid Analyzer [16] and Growth Detector [17]. This file contains data in several files: • Additional File 4 - Comparison of Measurement Modes.pdf: A summary of the results and notes on how the analysis was performed. • Cartoons: Cartoon representations of raw measurements generated in DR Engine (.png file formats). • CM Engine: the original images analyzed by CM Engine (.tif). • Growth Detector Data: Original images (.tif files) and results of running Growth Detector (.png files). • HT Colony Grid Data: Original images (.jpg) and results of running HT Colony Grid Analyzer (.dat and .png files). [file 1471-2105-11-353-S4.ZIP › Growth Detector Data/384-3 GrowthDetector 'true colonies'.PNG]

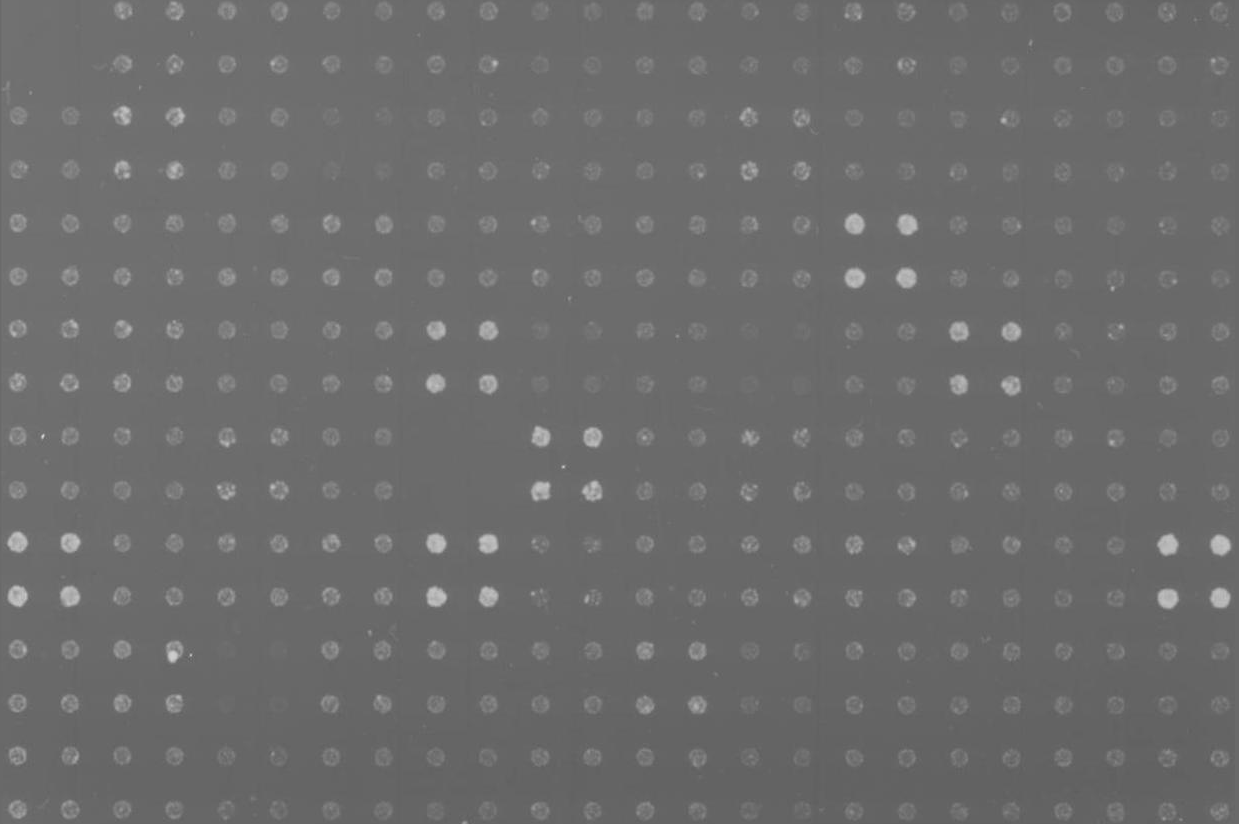

Supplement: Additional File 4 — Comparison of Measurement Modes (Dittmar et al, additional file 4.zip). This file contains data comparing CM Engine's three measurement modes to HT Colony Grid Analyzer [16] and Growth Detector [17]. This file contains data in several files: • Additional File 4 - Comparison of Measurement Modes.pdf: A summary of the results and notes on how the analysis was performed. • Cartoons: Cartoon representations of raw measurements generated in DR Engine (.png file formats). • CM Engine: the original images analyzed by CM Engine (.tif). • Growth Detector Data: Original images (.tif files) and results of running Growth Detector (.png files). • HT Colony Grid Data: Original images (.jpg) and results of running HT Colony Grid Analyzer (.dat and .png files). [file 1471-2105-11-353-S4.ZIP › Growth Detector Data/384-3.tif]

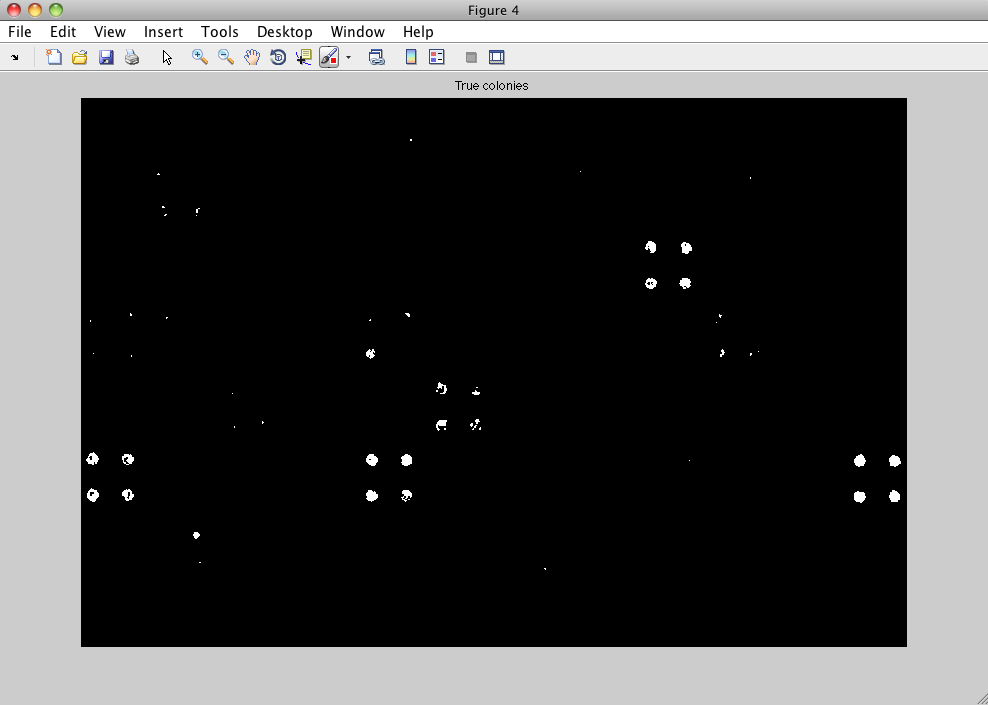

Supplement: Additional File 4 — Comparison of Measurement Modes (Dittmar et al, additional file 4.zip). This file contains data comparing CM Engine's three measurement modes to HT Colony Grid Analyzer [16] and Growth Detector [17]. This file contains data in several files: • Additional File 4 - Comparison of Measurement Modes.pdf: A summary of the results and notes on how the analysis was performed. • Cartoons: Cartoon representations of raw measurements generated in DR Engine (.png file formats). • CM Engine: the original images analyzed by CM Engine (.tif). • Growth Detector Data: Original images (.tif files) and results of running Growth Detector (.png files). • HT Colony Grid Data: Original images (.jpg) and results of running HT Colony Grid Analyzer (.dat and .png files). [file 1471-2105-11-353-S4.ZIP › Growth Detector Data/384-5 GrowthDetector 'true colonies'.PNG]

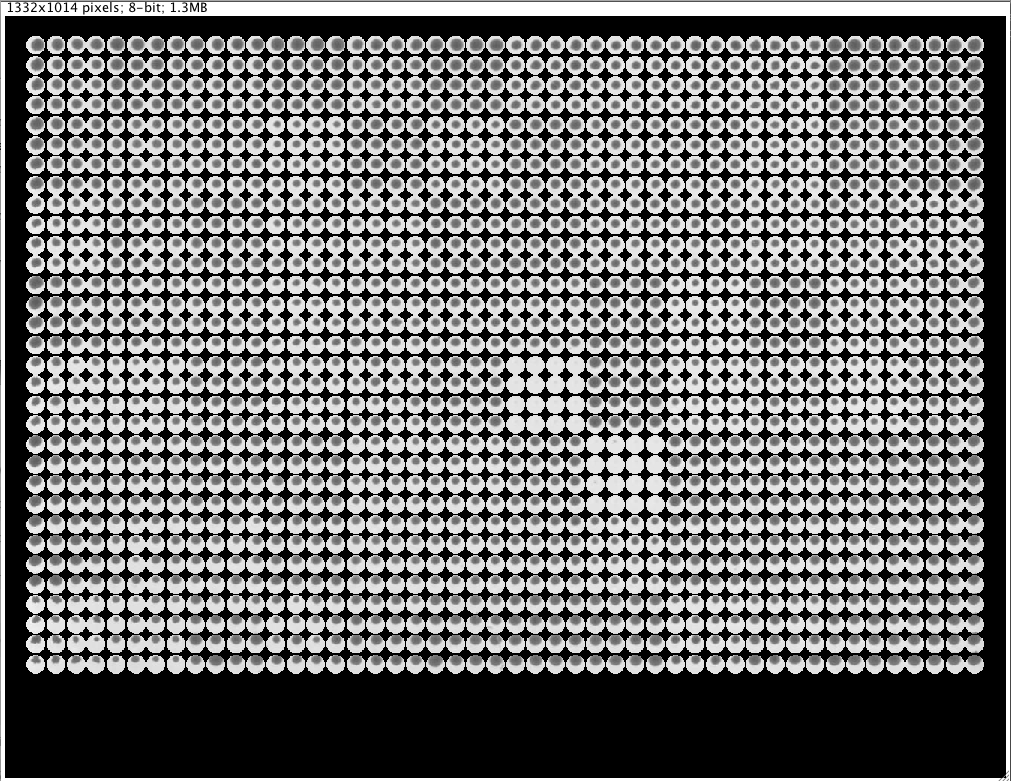

Supplement: Additional File 4 — Comparison of Measurement Modes (Dittmar et al, additional file 4.zip). This file contains data comparing CM Engine's three measurement modes to HT Colony Grid Analyzer [16] and Growth Detector [17]. This file contains data in several files: • Additional File 4 - Comparison of Measurement Modes.pdf: A summary of the results and notes on how the analysis was performed. • Cartoons: Cartoon representations of raw measurements generated in DR Engine (.png file formats). • CM Engine: the original images analyzed by CM Engine (.tif). • Growth Detector Data: Original images (.tif files) and results of running Growth Detector (.png files). • HT Colony Grid Data: Original images (.jpg) and results of running HT Colony Grid Analyzer (.dat and .png files). [file 1471-2105-11-353-S4.ZIP › HT Colony Grid Data/1536-1-mask.png]

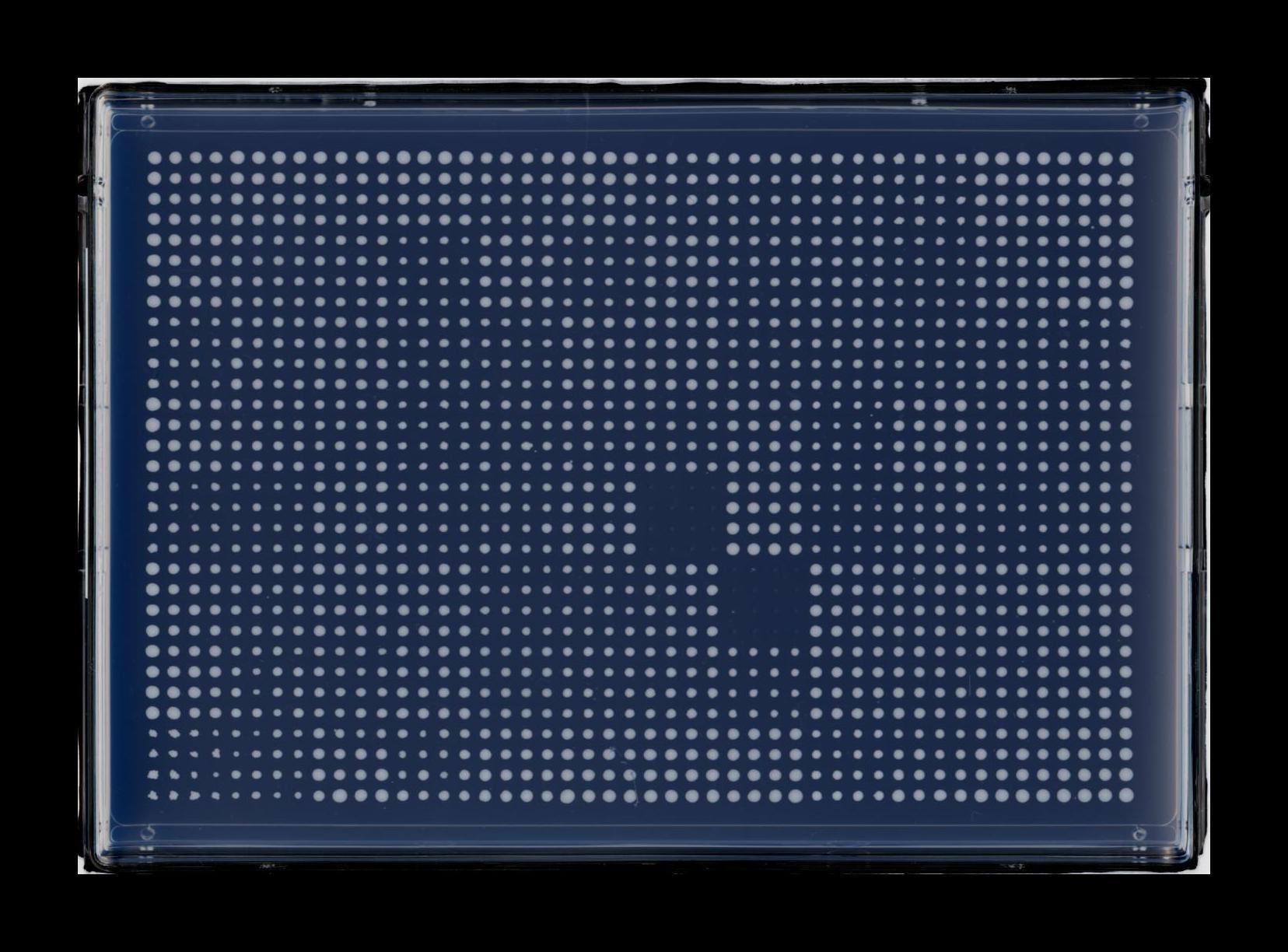

Supplement: Additional File 4 — Comparison of Measurement Modes (Dittmar et al, additional file 4.zip). This file contains data comparing CM Engine's three measurement modes to HT Colony Grid Analyzer [16] and Growth Detector [17]. This file contains data in several files: • Additional File 4 - Comparison of Measurement Modes.pdf: A summary of the results and notes on how the analysis was performed. • Cartoons: Cartoon representations of raw measurements generated in DR Engine (.png file formats). • CM Engine: the original images analyzed by CM Engine (.tif). • Growth Detector Data: Original images (.tif files) and results of running Growth Detector (.png files). • HT Colony Grid Data: Original images (.jpg) and results of running HT Colony Grid Analyzer (.dat and .png files). [file 1471-2105-11-353-S4.ZIP › HT Colony Grid Data/1536-1.jpg]

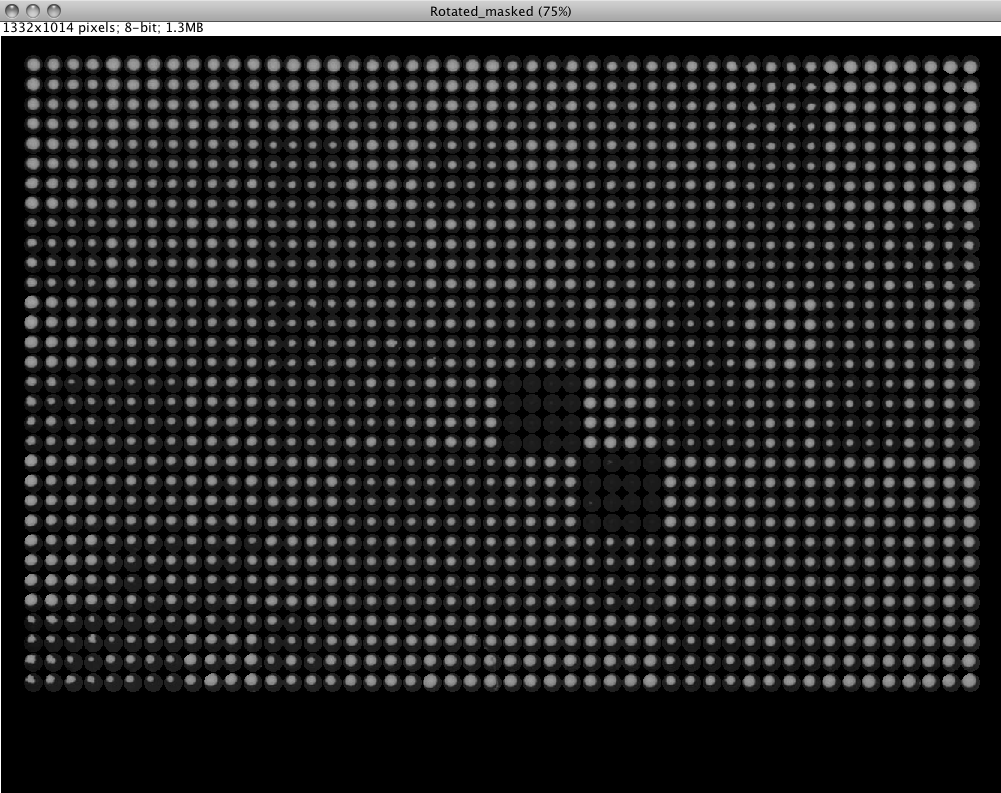

Supplement: Additional File 4 — Comparison of Measurement Modes (Dittmar et al, additional file 4.zip). This file contains data comparing CM Engine's three measurement modes to HT Colony Grid Analyzer [16] and Growth Detector [17]. This file contains data in several files: • Additional File 4 - Comparison of Measurement Modes.pdf: A summary of the results and notes on how the analysis was performed. • Cartoons: Cartoon representations of raw measurements generated in DR Engine (.png file formats). • CM Engine: the original images analyzed by CM Engine (.tif). • Growth Detector Data: Original images (.tif files) and results of running Growth Detector (.png files). • HT Colony Grid Data: Original images (.jpg) and results of running HT Colony Grid Analyzer (.dat and .png files). [file 1471-2105-11-353-S4.ZIP › HT Colony Grid Data/1536-1a mask.png]

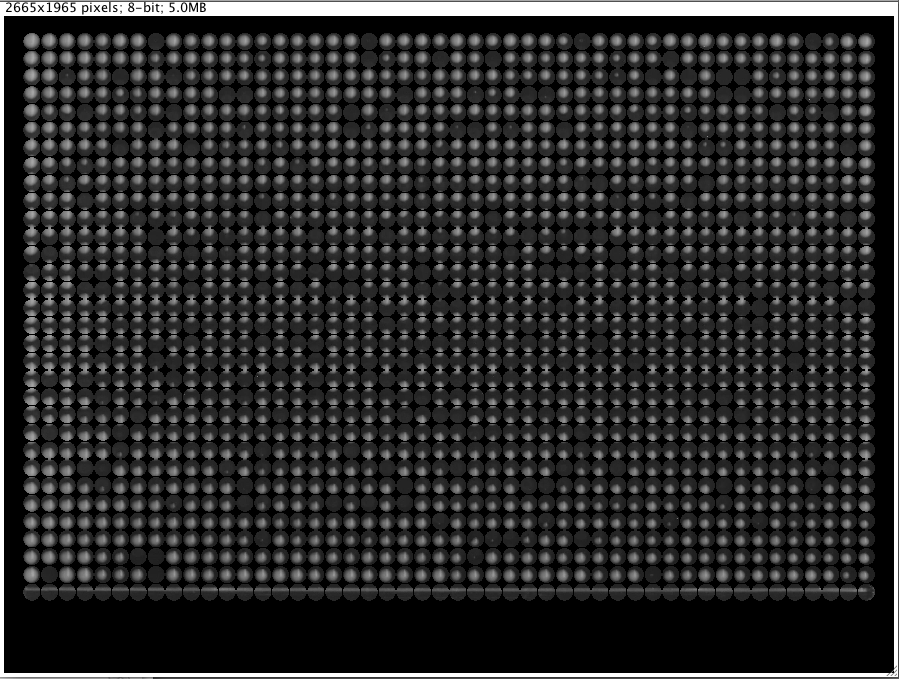

Supplement: Additional File 4 — Comparison of Measurement Modes (Dittmar et al, additional file 4.zip). This file contains data comparing CM Engine's three measurement modes to HT Colony Grid Analyzer [16] and Growth Detector [17]. This file contains data in several files: • Additional File 4 - Comparison of Measurement Modes.pdf: A summary of the results and notes on how the analysis was performed. • Cartoons: Cartoon representations of raw measurements generated in DR Engine (.png file formats). • CM Engine: the original images analyzed by CM Engine (.tif). • Growth Detector Data: Original images (.tif files) and results of running Growth Detector (.png files). • HT Colony Grid Data: Original images (.jpg) and results of running HT Colony Grid Analyzer (.dat and .png files). [file 1471-2105-11-353-S4.ZIP › HT Colony Grid Data/1536-2 mask.png]

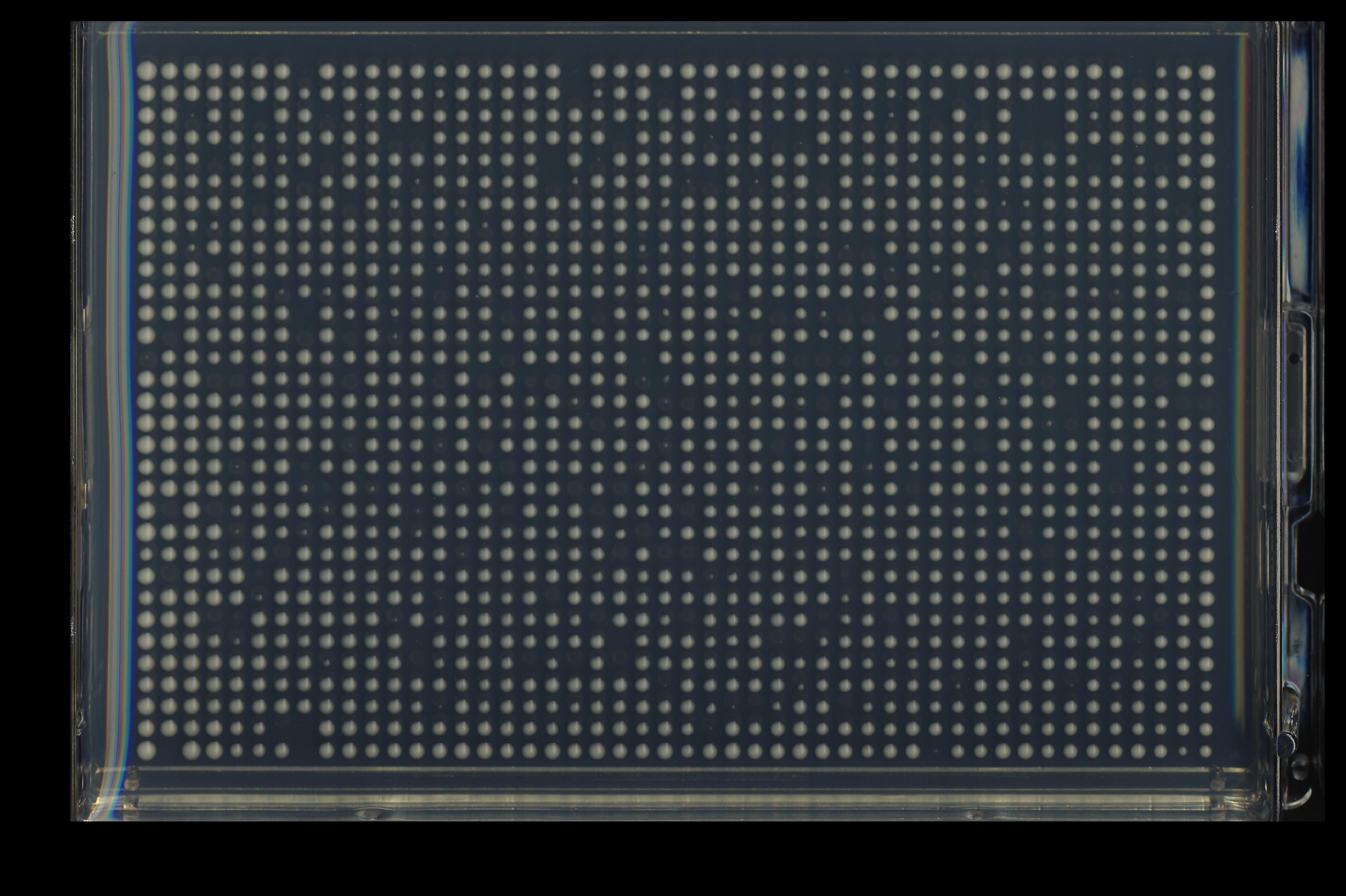

Supplement: Additional File 4 — Comparison of Measurement Modes (Dittmar et al, additional file 4.zip). This file contains data comparing CM Engine's three measurement modes to HT Colony Grid Analyzer [16] and Growth Detector [17]. This file contains data in several files: • Additional File 4 - Comparison of Measurement Modes.pdf: A summary of the results and notes on how the analysis was performed. • Cartoons: Cartoon representations of raw measurements generated in DR Engine (.png file formats). • CM Engine: the original images analyzed by CM Engine (.tif). • Growth Detector Data: Original images (.tif files) and results of running Growth Detector (.png files). • HT Colony Grid Data: Original images (.jpg) and results of running HT Colony Grid Analyzer (.dat and .png files). [file 1471-2105-11-353-S4.ZIP › HT Colony Grid Data/1536-2.jpg]

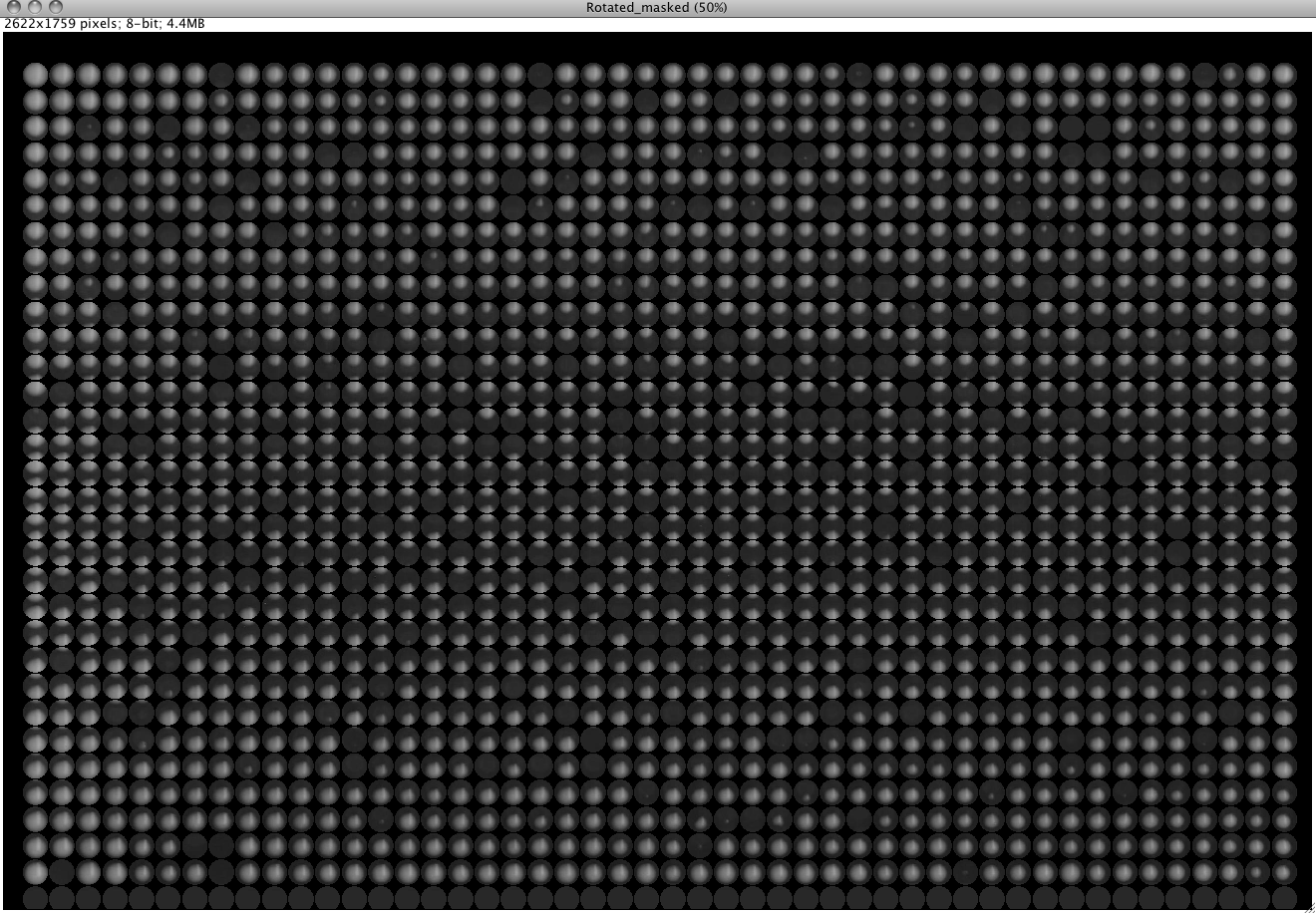

Supplement: Additional File 4 — Comparison of Measurement Modes (Dittmar et al, additional file 4.zip). This file contains data comparing CM Engine's three measurement modes to HT Colony Grid Analyzer [16] and Growth Detector [17]. This file contains data in several files: • Additional File 4 - Comparison of Measurement Modes.pdf: A summary of the results and notes on how the analysis was performed. • Cartoons: Cartoon representations of raw measurements generated in DR Engine (.png file formats). • CM Engine: the original images analyzed by CM Engine (.tif). • Growth Detector Data: Original images (.tif files) and results of running Growth Detector (.png files). • HT Colony Grid Data: Original images (.jpg) and results of running HT Colony Grid Analyzer (.dat and .png files). [file 1471-2105-11-353-S4.ZIP › HT Colony Grid Data/1536-3 mask.png]

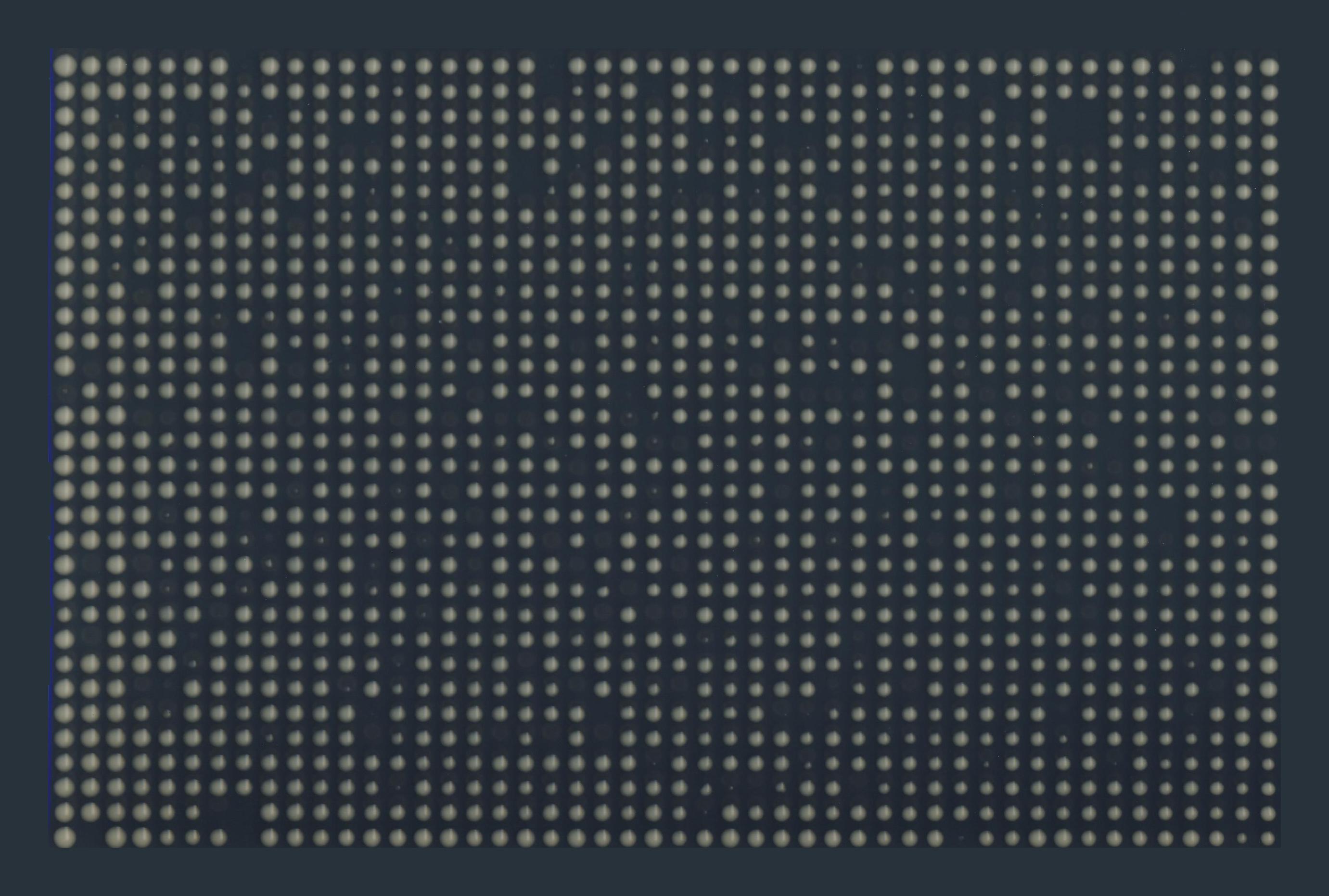

Supplement: Additional File 4 — Comparison of Measurement Modes (Dittmar et al, additional file 4.zip). This file contains data comparing CM Engine's three measurement modes to HT Colony Grid Analyzer [16] and Growth Detector [17]. This file contains data in several files: • Additional File 4 - Comparison of Measurement Modes.pdf: A summary of the results and notes on how the analysis was performed. • Cartoons: Cartoon representations of raw measurements generated in DR Engine (.png file formats). • CM Engine: the original images analyzed by CM Engine (.tif). • Growth Detector Data: Original images (.tif files) and results of running Growth Detector (.png files). • HT Colony Grid Data: Original images (.jpg) and results of running HT Colony Grid Analyzer (.dat and .png files). [file 1471-2105-11-353-S4.ZIP › HT Colony Grid Data/1536-3.jpg]

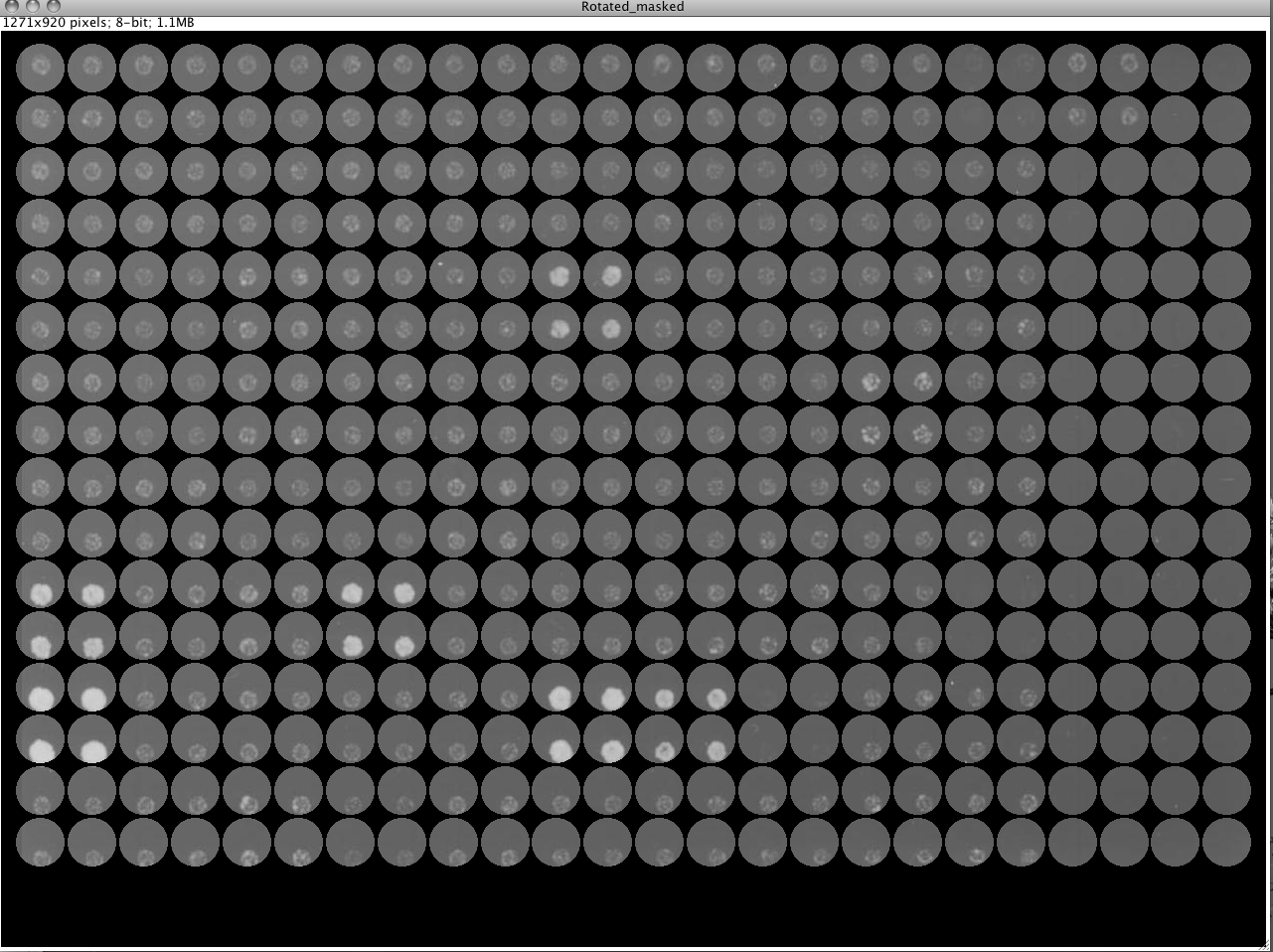

Supplement: Additional File 4 — Comparison of Measurement Modes (Dittmar et al, additional file 4.zip). This file contains data comparing CM Engine's three measurement modes to HT Colony Grid Analyzer [16] and Growth Detector [17]. This file contains data in several files: • Additional File 4 - Comparison of Measurement Modes.pdf: A summary of the results and notes on how the analysis was performed. • Cartoons: Cartoon representations of raw measurements generated in DR Engine (.png file formats). • CM Engine: the original images analyzed by CM Engine (.tif). • Growth Detector Data: Original images (.tif files) and results of running Growth Detector (.png files). • HT Colony Grid Data: Original images (.jpg) and results of running HT Colony Grid Analyzer (.dat and .png files). [file 1471-2105-11-353-S4.ZIP › HT Colony Grid Data/384-1 mask.png]

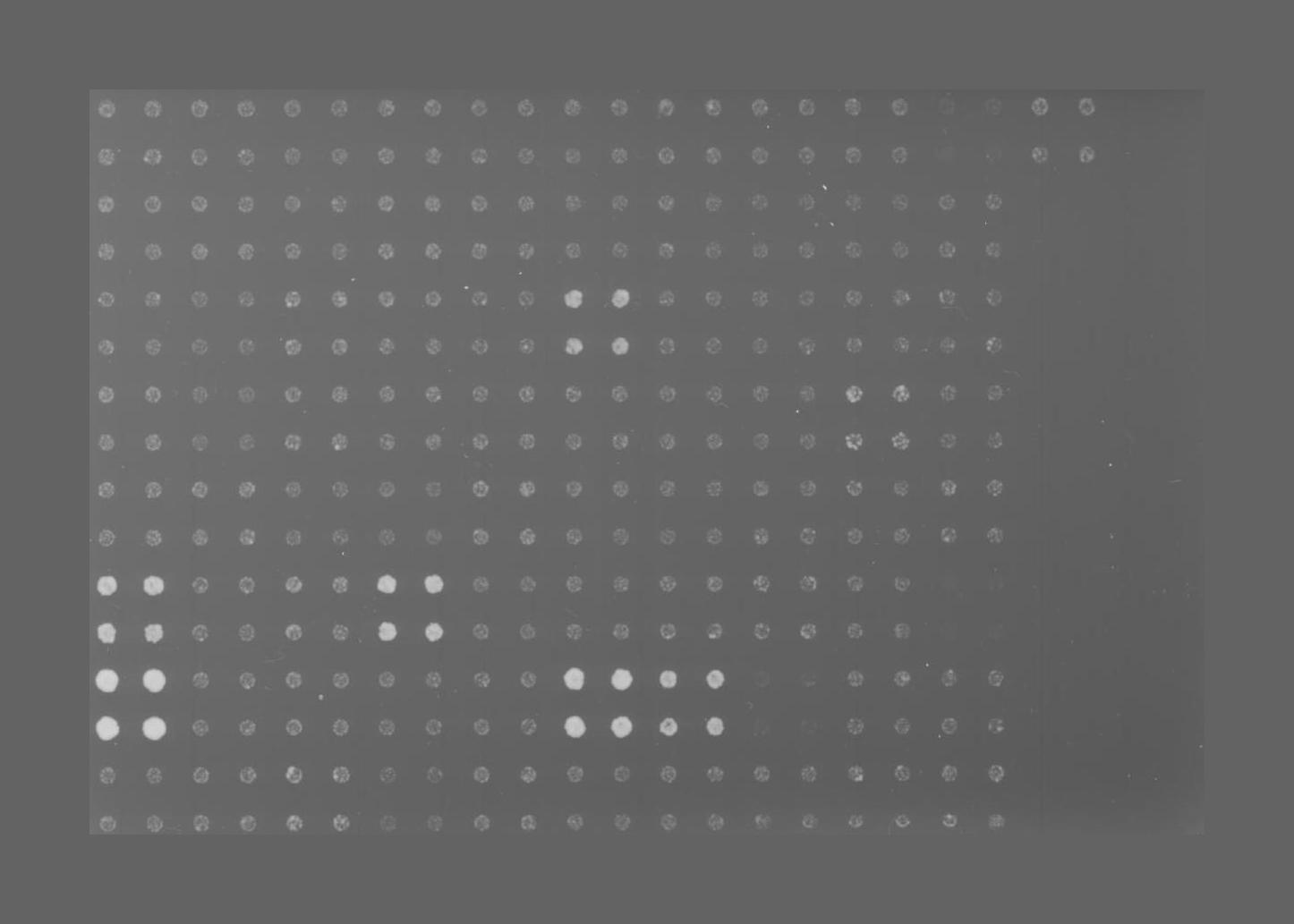

Supplement: Additional File 4 — Comparison of Measurement Modes (Dittmar et al, additional file 4.zip). This file contains data comparing CM Engine's three measurement modes to HT Colony Grid Analyzer [16] and Growth Detector [17]. This file contains data in several files: • Additional File 4 - Comparison of Measurement Modes.pdf: A summary of the results and notes on how the analysis was performed. • Cartoons: Cartoon representations of raw measurements generated in DR Engine (.png file formats). • CM Engine: the original images analyzed by CM Engine (.tif). • Growth Detector Data: Original images (.tif files) and results of running Growth Detector (.png files). • HT Colony Grid Data: Original images (.jpg) and results of running HT Colony Grid Analyzer (.dat and .png files). [file 1471-2105-11-353-S4.ZIP › HT Colony Grid Data/384-1.jpg]

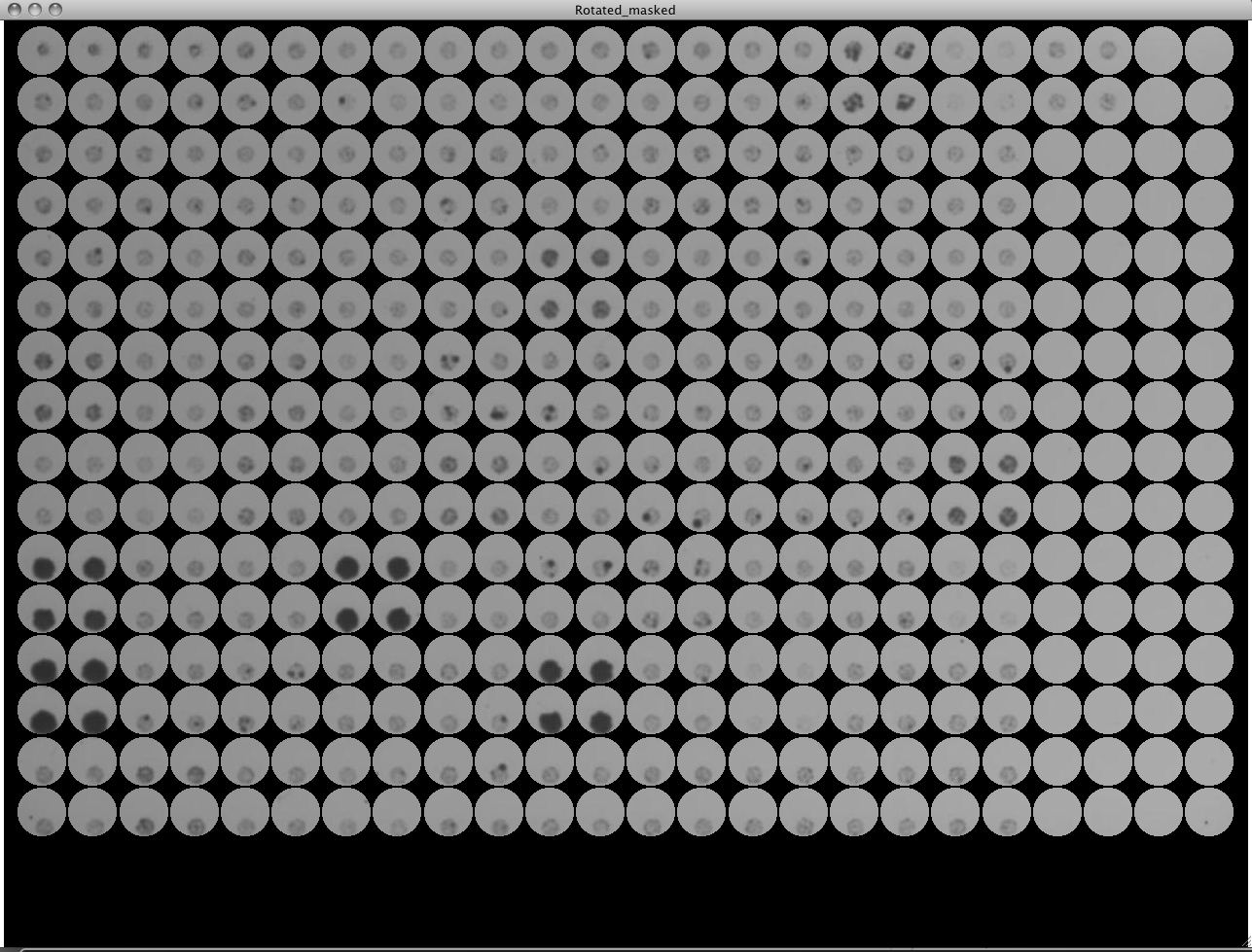

Supplement: Additional File 4 — Comparison of Measurement Modes (Dittmar et al, additional file 4.zip). This file contains data comparing CM Engine's three measurement modes to HT Colony Grid Analyzer [16] and Growth Detector [17]. This file contains data in several files: • Additional File 4 - Comparison of Measurement Modes.pdf: A summary of the results and notes on how the analysis was performed. • Cartoons: Cartoon representations of raw measurements generated in DR Engine (.png file formats). • CM Engine: the original images analyzed by CM Engine (.tif). • Growth Detector Data: Original images (.tif files) and results of running Growth Detector (.png files). • HT Colony Grid Data: Original images (.jpg) and results of running HT Colony Grid Analyzer (.dat and .png files). [file 1471-2105-11-353-S4.ZIP › HT Colony Grid Data/384-2 mask.png]

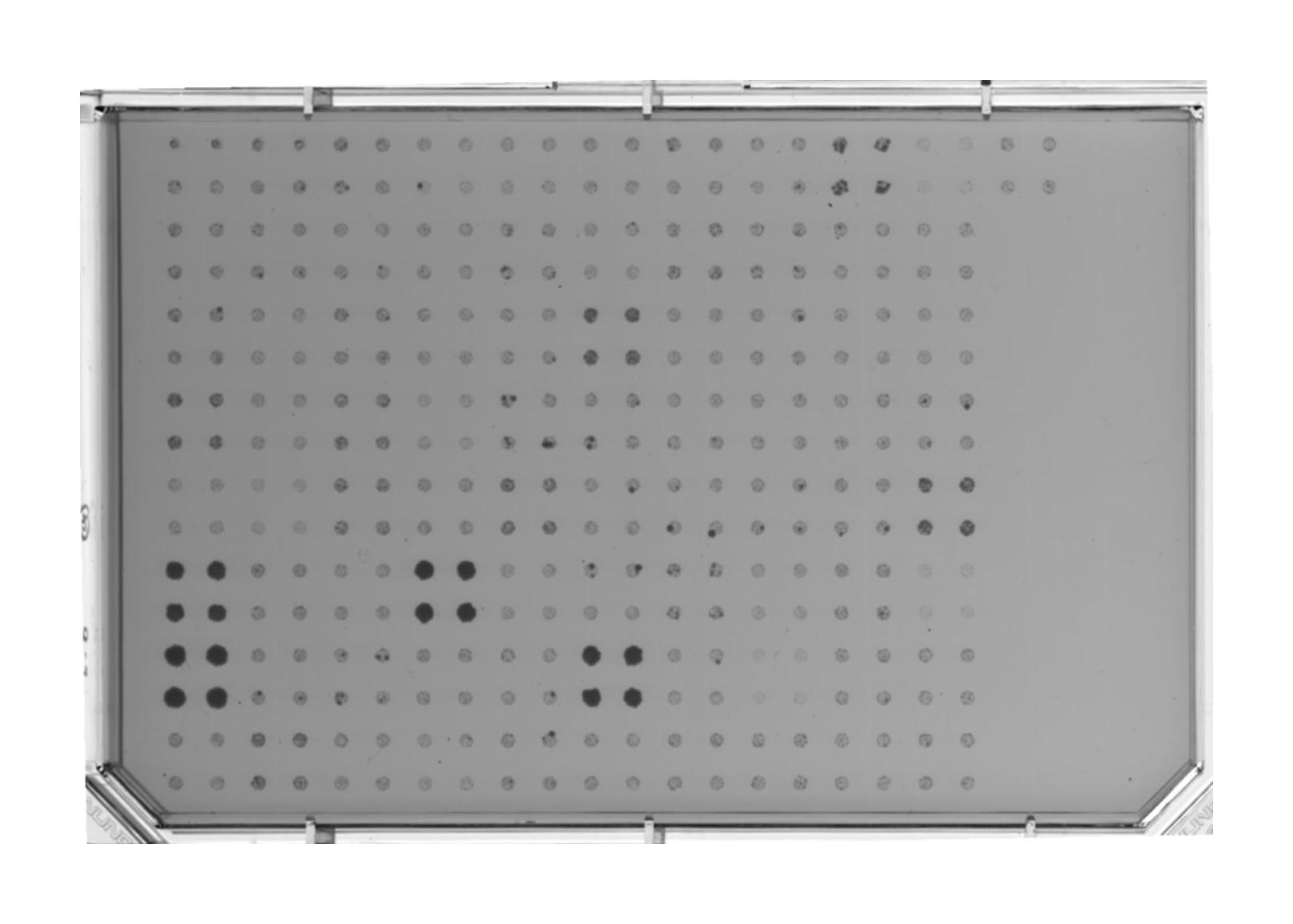

Supplement: Additional File 4 — Comparison of Measurement Modes (Dittmar et al, additional file 4.zip). This file contains data comparing CM Engine's three measurement modes to HT Colony Grid Analyzer [16] and Growth Detector [17]. This file contains data in several files: • Additional File 4 - Comparison of Measurement Modes.pdf: A summary of the results and notes on how the analysis was performed. • Cartoons: Cartoon representations of raw measurements generated in DR Engine (.png file formats). • CM Engine: the original images analyzed by CM Engine (.tif). • Growth Detector Data: Original images (.tif files) and results of running Growth Detector (.png files). • HT Colony Grid Data: Original images (.jpg) and results of running HT Colony Grid Analyzer (.dat and .png files). [file 1471-2105-11-353-S4.ZIP › HT Colony Grid Data/384-2.jpg]

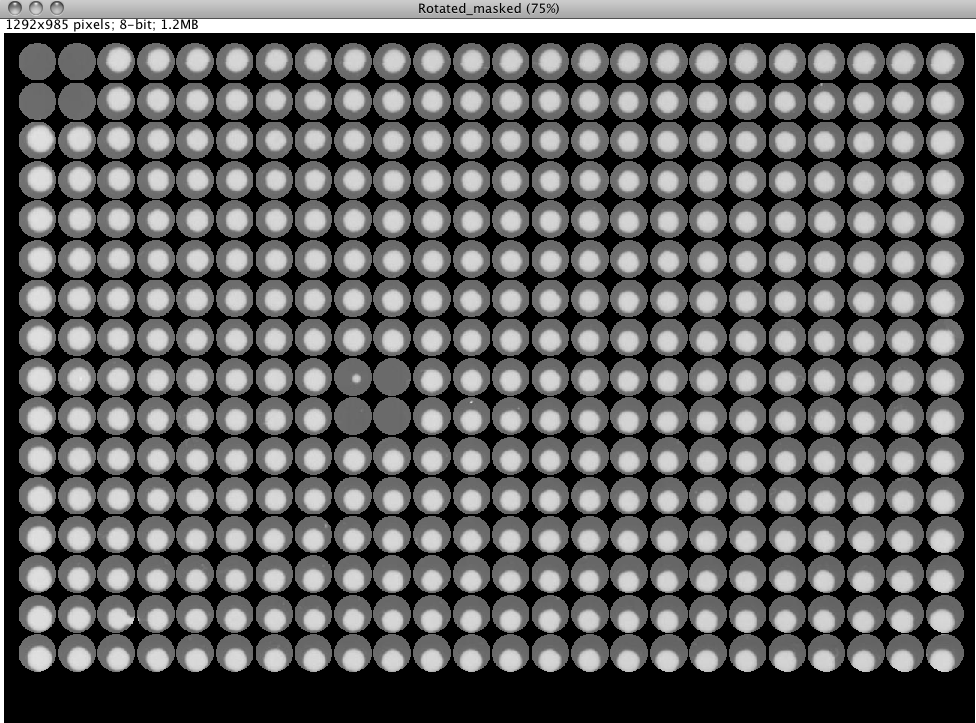

Supplement: Additional File 4 — Comparison of Measurement Modes (Dittmar et al, additional file 4.zip). This file contains data comparing CM Engine's three measurement modes to HT Colony Grid Analyzer [16] and Growth Detector [17]. This file contains data in several files: • Additional File 4 - Comparison of Measurement Modes.pdf: A summary of the results and notes on how the analysis was performed. • Cartoons: Cartoon representations of raw measurements generated in DR Engine (.png file formats). • CM Engine: the original images analyzed by CM Engine (.tif). • Growth Detector Data: Original images (.tif files) and results of running Growth Detector (.png files). • HT Colony Grid Data: Original images (.jpg) and results of running HT Colony Grid Analyzer (.dat and .png files). [file 1471-2105-11-353-S4.ZIP › HT Colony Grid Data/384-3 mask.png]

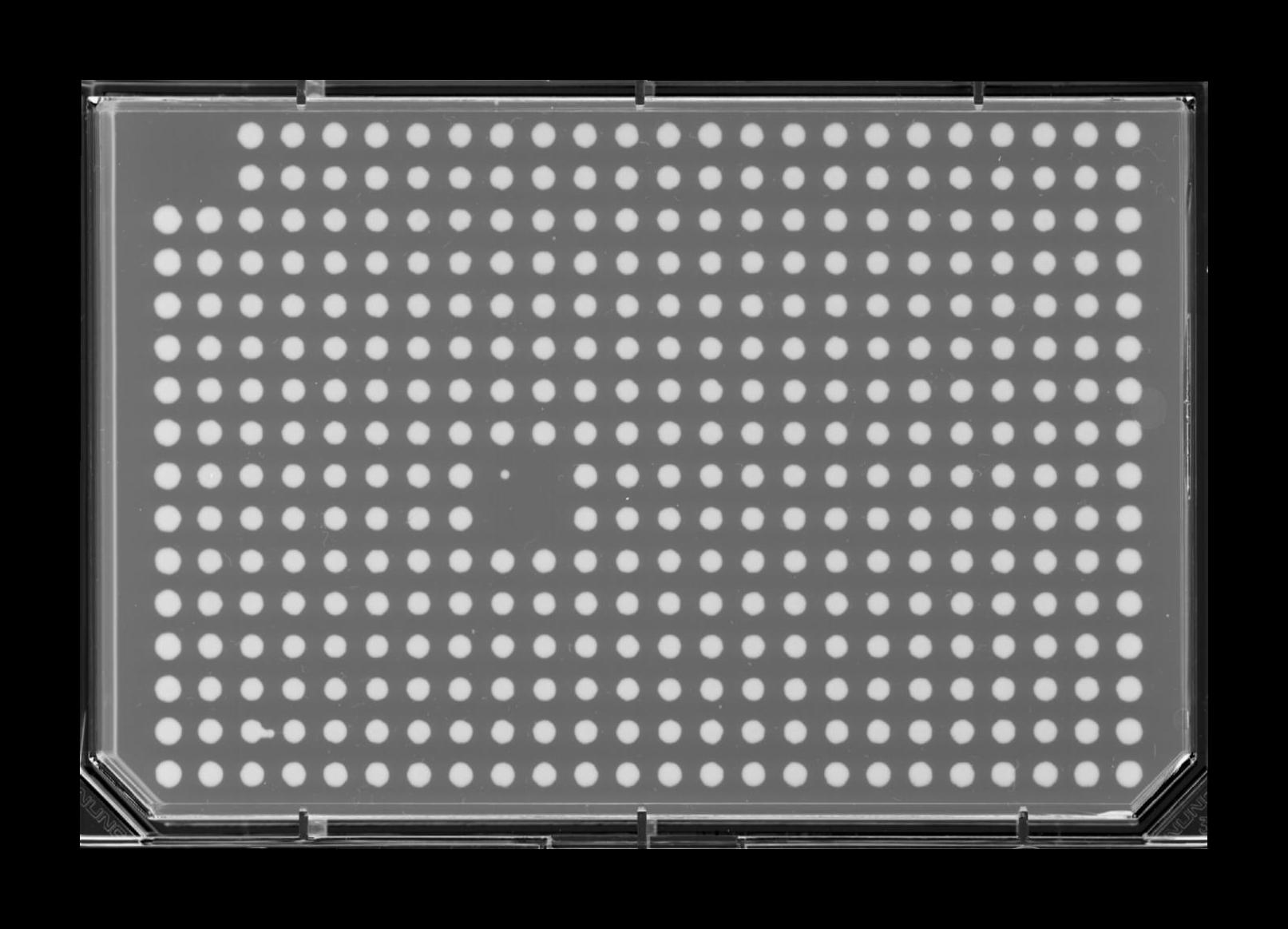

Supplement: Additional File 4 — Comparison of Measurement Modes (Dittmar et al, additional file 4.zip). This file contains data comparing CM Engine's three measurement modes to HT Colony Grid Analyzer [16] and Growth Detector [17]. This file contains data in several files: • Additional File 4 - Comparison of Measurement Modes.pdf: A summary of the results and notes on how the analysis was performed. • Cartoons: Cartoon representations of raw measurements generated in DR Engine (.png file formats). • CM Engine: the original images analyzed by CM Engine (.tif). • Growth Detector Data: Original images (.tif files) and results of running Growth Detector (.png files). • HT Colony Grid Data: Original images (.jpg) and results of running HT Colony Grid Analyzer (.dat and .png files). [file 1471-2105-11-353-S4.ZIP › HT Colony Grid Data/384-3.jpg]

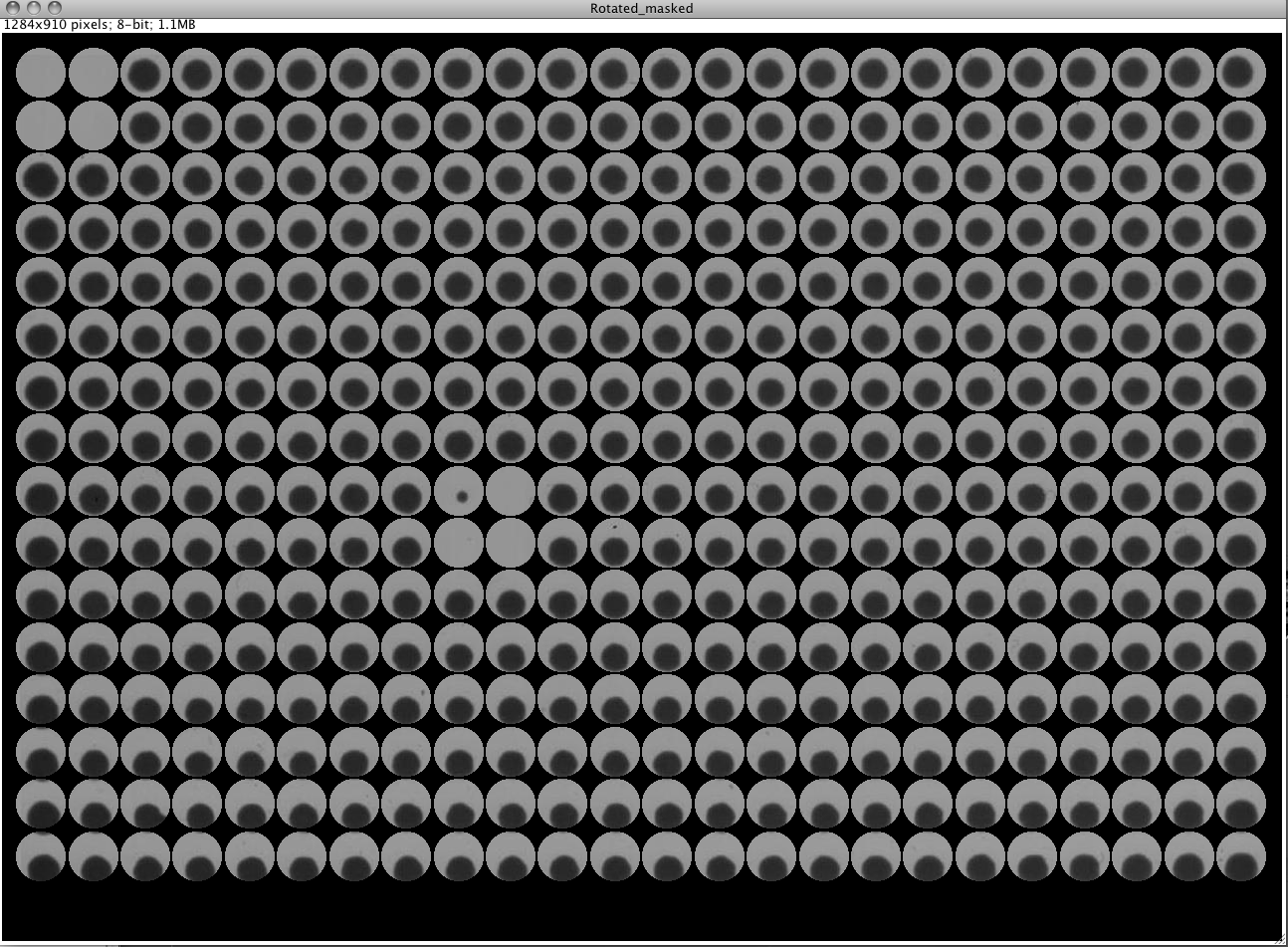

Supplement: Additional File 4 — Comparison of Measurement Modes (Dittmar et al, additional file 4.zip). This file contains data comparing CM Engine's three measurement modes to HT Colony Grid Analyzer [16] and Growth Detector [17]. This file contains data in several files: • Additional File 4 - Comparison of Measurement Modes.pdf: A summary of the results and notes on how the analysis was performed. • Cartoons: Cartoon representations of raw measurements generated in DR Engine (.png file formats). • CM Engine: the original images analyzed by CM Engine (.tif). • Growth Detector Data: Original images (.tif files) and results of running Growth Detector (.png files). • HT Colony Grid Data: Original images (.jpg) and results of running HT Colony Grid Analyzer (.dat and .png files). [file 1471-2105-11-353-S4.ZIP › HT Colony Grid Data/384-4 mask.png]

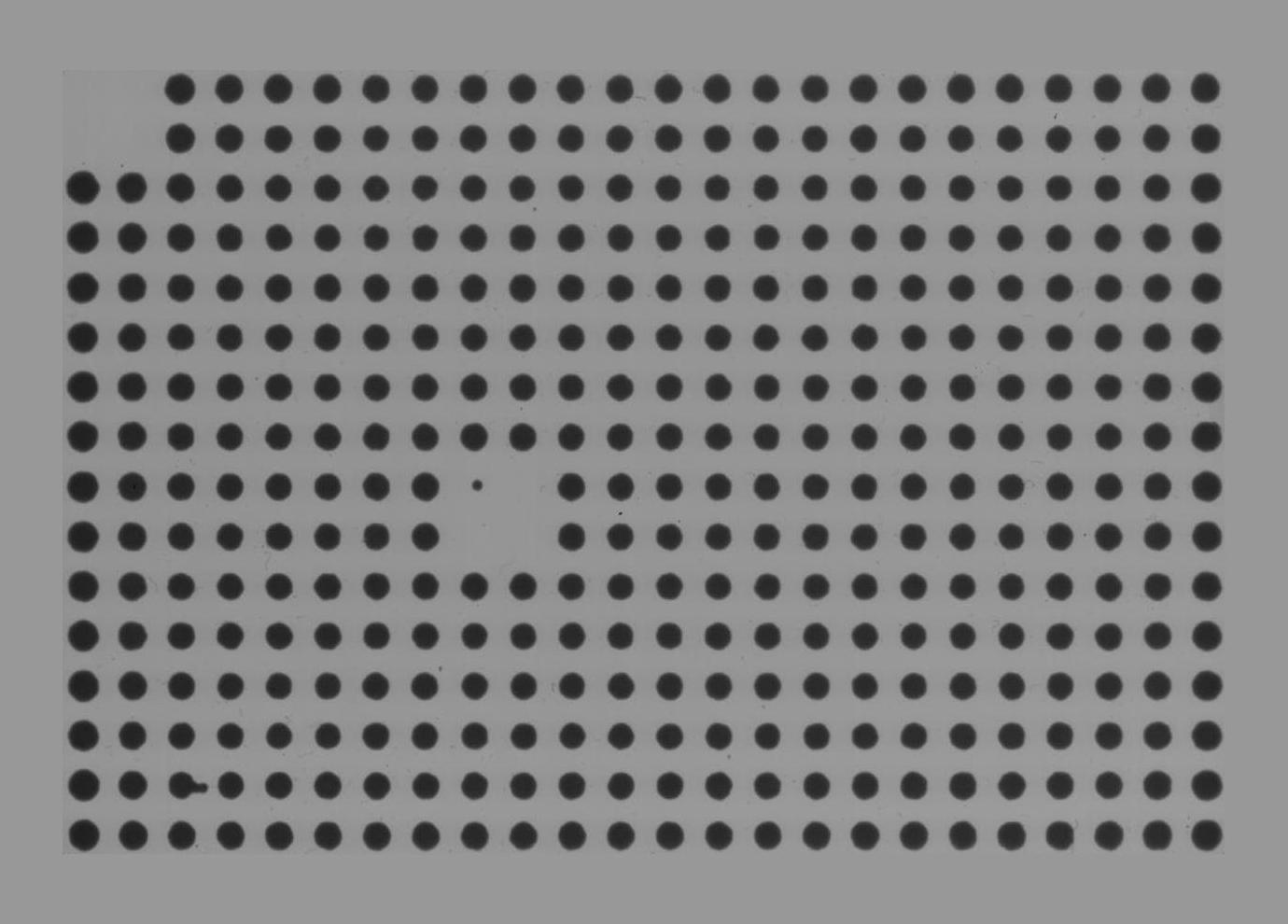

Supplement: Additional File 4 — Comparison of Measurement Modes (Dittmar et al, additional file 4.zip). This file contains data comparing CM Engine's three measurement modes to HT Colony Grid Analyzer [16] and Growth Detector [17]. This file contains data in several files: • Additional File 4 - Comparison of Measurement Modes.pdf: A summary of the results and notes on how the analysis was performed. • Cartoons: Cartoon representations of raw measurements generated in DR Engine (.png file formats). • CM Engine: the original images analyzed by CM Engine (.tif). • Growth Detector Data: Original images (.tif files) and results of running Growth Detector (.png files). • HT Colony Grid Data: Original images (.jpg) and results of running HT Colony Grid Analyzer (.dat and .png files). [file 1471-2105-11-353-S4.ZIP › HT Colony Grid Data/384-4.jpg]

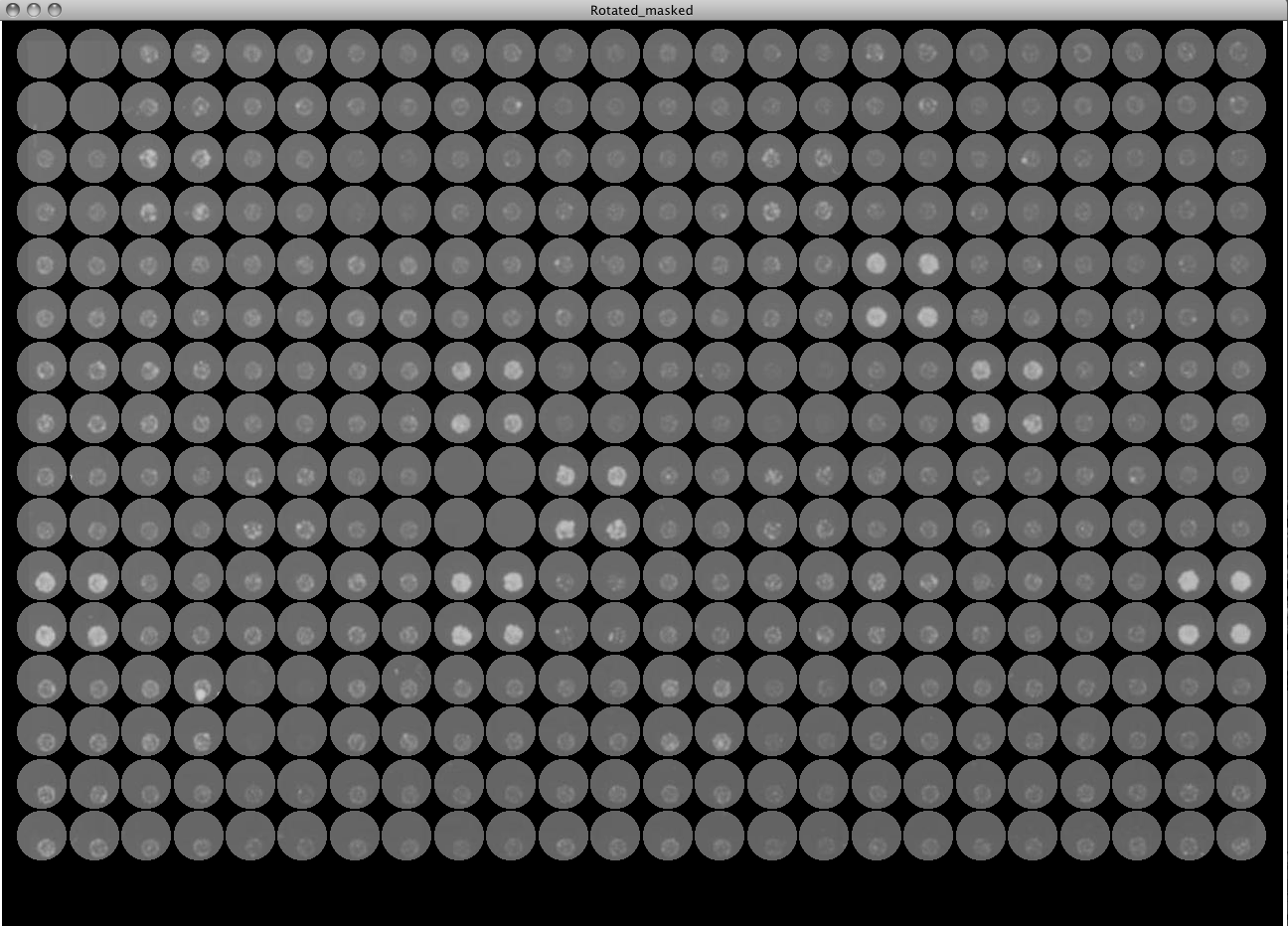

Supplement: Additional File 4 — Comparison of Measurement Modes (Dittmar et al, additional file 4.zip). This file contains data comparing CM Engine's three measurement modes to HT Colony Grid Analyzer [16] and Growth Detector [17]. This file contains data in several files: • Additional File 4 - Comparison of Measurement Modes.pdf: A summary of the results and notes on how the analysis was performed. • Cartoons: Cartoon representations of raw measurements generated in DR Engine (.png file formats). • CM Engine: the original images analyzed by CM Engine (.tif). • Growth Detector Data: Original images (.tif files) and results of running Growth Detector (.png files). • HT Colony Grid Data: Original images (.jpg) and results of running HT Colony Grid Analyzer (.dat and .png files). [file 1471-2105-11-353-S4.ZIP › HT Colony Grid Data/384-5 - mask.png]

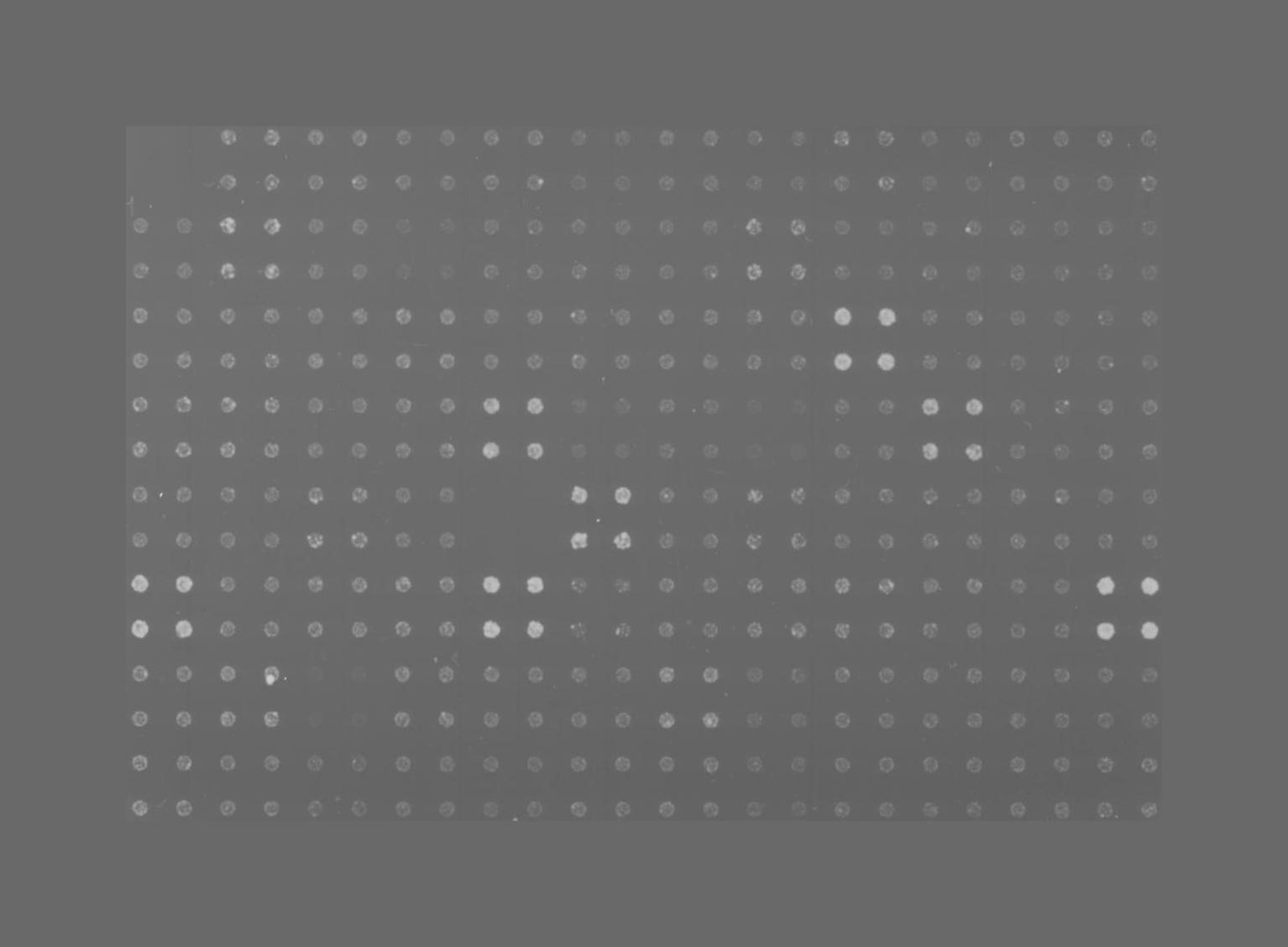

Supplement: Additional File 4 — Comparison of Measurement Modes (Dittmar et al, additional file 4.zip). This file contains data comparing CM Engine's three measurement modes to HT Colony Grid Analyzer [16] and Growth Detector [17]. This file contains data in several files: • Additional File 4 - Comparison of Measurement Modes.pdf: A summary of the results and notes on how the analysis was performed. • Cartoons: Cartoon representations of raw measurements generated in DR Engine (.png file formats). • CM Engine: the original images analyzed by CM Engine (.tif). • Growth Detector Data: Original images (.tif files) and results of running Growth Detector (.png files). • HT Colony Grid Data: Original images (.jpg) and results of running HT Colony Grid Analyzer (.dat and .png files). [file 1471-2105-11-353-S4.ZIP › HT Colony Grid Data/384-5.jpg]
