# Supplementary material for: ScreenMill: A freely available software suite for growth measurement, analysis and visualization of high-throughput screen data
Source: BMC Bioinformatics. 2010 Jun 28;11:353. doi: 10.1186/1471-2105-11-353 (PMC2909220; doi:10.1186/1471-2105-11-353)
Supplement: Additional File 5 — Source code for CM, DR and SV Engines (Dittmar et al, additional file 5.zip). This file include two zip files: • ScreenMill - CM Engine.zip: contains two copies the CM Engine. One for ImageJ version 1.42, one for version 1.43+ (.txt files). • ScreenMill - DR and SV Engines.zip: contains the HTML, CSS, JavaScript, Image, and CGI (Perl) files needed to run DR and SV Engines. [file 1471-2105-11-353-S5.ZIP › Dittmar et al, addtional file 5/ScreenMill DR and SV Engines.zip!/ScreenMill DR and SV Engines/DR Engine setup.html]

ScreenMill - Data Review Engine

# *ScreenMill - **D**ata **R**eview Engine*

File Information


|  |  |  |  |
| --- | --- | --- | --- |
| Log File: |  | Comparer Query: | Enter the query identifier to use as your comparer. This must be the same as it appears in your log file. |
| Key File: | Select the library screened. You may upload your own key file by selecting the "UPLOAD YOUR OWN" option. You may disconnect your data from a key file by selected the "NO KEY FILE" option.  Please select:  Rothstein HB-1536 (4 reps, MAT alpha) Rothstein HB-384 (1 reps, MAT alpha) Rothstein-96 (1 reps, MAT alpha)  Rothstein-384 (4 reps, MAT alpha)  Curie Institute-384 (4 reps, MAT alpha) Hieter Lab-1536 (1 reps, MAT a)  ⇒⇒ UPLOAD YOUR OWN ⇐⇐ ⇒⇒ NO KEY FILE ⇐⇐ | Description: | Enter a description that you will be able to remember. This will be used to help identify this analysis. Acceptable characters include a-z, 0-9, and the following symbols ' " - ( ) . , \_ ? ! |
|  | Rothstein HB-1536 (4 reps, MAT alpha) ⇒ Density: 1536. Replicates: 4. Library Mating Type: MAT alpha. | | |

  

Statistical Test (mouse-over for details)

|  |  |  |
| --- | --- | --- |
| Data is assumed to form a normal distribution, from this z-scores and p-values are calculated.  Normal Distribution | An unpaired t-test, assuming unequal variance among samples is performed between a given set of replicates on a comparer plate to the corresponding replicates on a experimental plate.  Unpaired T-Test | The Mann-Whitney test, with continuity correction, will be performed. This test is a non-parametric test for assessing whether two samples of observations come from the same distribution. No assumptions about the distribution of data are made.  Mann-Whitney Test |

|  |  |  |
| --- | --- | --- |
| Bonferroni correction *WILL* be performed.  Bonferroni correct p-values | Bonferroni correction *WILL NOT* be performed.  *DO NOT* Bonferroni correct p-values. | What is Bonferroni correction? |

  

Data Normalization Method (mouse-over for details)

|  |  |  |
| --- | --- | --- |
| Data on a given plate are divided by the median value on the plate to normalize. Unless changed in the advanced options, below, zero values are ignored  Plate Median | Data on a plate are normalized to the median value of designated control data on the same plate. In your key file, rows with the phrase "Positive Control" in the ID column will be considered as control data.  Designated Controls | No data normalization is performed.  Do NOT normalize |

  


Advanced options

|  |  |  |  |
| --- | --- | --- | --- |
| Run Global Exclusion? | Default value = "yes". Should global exclusion be run? Select "No" to disable Global Exclusion. Disabling this algorithm will prevent data from being highlighted for exclusion by the global exclusion algorithm although you may still manually exclude data.  Yes No | Yellow Highlight Cutoff: | Default = 25% of plate normalization value. Cutoff below which data will be highlighted in yellow in cartoon representations of plates. Modifying this parameter will affect the global exclusion algorithm. Increasing this value may increase the number of data highlighted in yellow and therefore may increase the number of data highlighted in red for exclusion. Decreasing it will have the opposite effect.  0% 5% 10% 15% 20% 25% 30% 35% 40% 45% 50% 55% 60% 65% 70% 75% 80% 85% 90% 95% |
| Run Replicate Exclusion? | Default value = yes. Allows for the replicate exclusion algorithm to be disabled. This parameter only affects processing when analyzing data from a screen with 4 replicates (2 x 2). Disabling this algorithm prevents the exclusion of data based on the comparative growth within a set of 4 replicates.  Yes No | Ignore Zero Values: | Default value = yes. This parameter affects the way data are normalized to the "Plate Median." Uncheck this box to include "zero" values in the plate median calculation.  Yes No |

  


Incubation Times (Rothstein Lab SDL screens)

|  |  |  |  |
| --- | --- | --- | --- |
| Check this box to store additional information | | | |
| Start Date: |  | 1st Gal -Leu Incubation: | select the amount of time you incubated during the 1st round of Gal -Leu selection.  Please select: 12 hours 18 hours 24 hours 30 hours 36 hours 42 hours 48 hours |
| Library Replicates: | select the number of times you replicated the library on to fresh media from 4°C prior to mating.  Please select: 1 2 3 | 2nd Gal -Leu Incubation: | select the amount of time you incubated during the 2nd round of Gal -Leu selection.  Please select: n/a 12 18 24 30 36 42 48 |
| Mating Time: | select amount of time you mated on YPD.  Please select: 4 hours 6 hours 8 hours 10 hours 12 hours 14 hours 16 hours | Final Incubation: | select the amount of time your final selection plates were incubated.  Please select: 24 30 36 42 48 54 60 66 72 78 84 |
