# Supplementary material for: ScreenMill: A freely available software suite for growth measurement, analysis and visualization of high-throughput screen data
Source: BMC Bioinformatics. 2010 Jun 28;11:353. doi: 10.1186/1471-2105-11-353 (PMC2909220; doi:10.1186/1471-2105-11-353)
Supplement: Additional File 5 — Source code for CM, DR and SV Engines (Dittmar et al, additional file 5.zip). This file include two zip files: • ScreenMill - CM Engine.zip: contains two copies the CM Engine. One for ImageJ version 1.42, one for version 1.43+ (.txt files). • ScreenMill - DR and SV Engines.zip: contains the HTML, CSS, JavaScript, Image, and CGI (Perl) files needed to run DR and SV Engines. [file 1471-2105-11-353-S5.ZIP › Dittmar et al, addtional file 5/ScreenMill DR and SV Engines.zip!/ScreenMill DR and SV Engines/DR Engine.html]

DR Engine - Page 1

# *Data Review Engine*

*Please select the colonies below that you would like to exclude from statistical consideration.*

**Notes**

- Analysis progress is automatically saved move to a new page. To save your progress without being redirected to a new page click the "Save Progress" button at the bottom of the page.
- Clicking on a colony will toggle its exclusion status.
- Clicking on a row heading (one of the letters on the left of a plate) will toggle the exclusion of the entire row.
- Clicking on a column heading (one of the numbers above a plate) will toggle the exclusion of the entire column.

***Yellow = dead***

***Red = excluded***

***Light Red = excluded on "comparer" plate***
