# Supplementary material for: ScreenMill: A freely available software suite for growth measurement, analysis and visualization of high-throughput screen data
Source: BMC Bioinformatics. 2010 Jun 28;11:353. doi: 10.1186/1471-2105-11-353 (PMC2909220; doi:10.1186/1471-2105-11-353)
Supplement: Additional File 5 — Source code for CM, DR and SV Engines (Dittmar et al, additional file 5.zip). This file include two zip files: • ScreenMill - CM Engine.zip: contains two copies the CM Engine. One for ImageJ version 1.42, one for version 1.43+ (.txt files). • ScreenMill - DR and SV Engines.zip: contains the HTML, CSS, JavaScript, Image, and CGI (Perl) files needed to run DR and SV Engines. [file 1471-2105-11-353-S5.ZIP › Dittmar et al, addtional file 5/ScreenMill DR and SV Engines.zip!/ScreenMill DR and SV Engines/SV Engine setup.html]

ScreenMill - Screen Visualization Engine

# *ScreenMill - Screen Visualization Engine*

*A tool to visually compare data.*

**Comparer Query:**

Enter the query you used as your comparer. This must be the same as it appears in your ScreenMillStats-All.txt and log files.

**Log File:**

**ScreenMillStats-All Data File:**
