# Supplementary material for: ScreenMill: A freely available software suite for growth measurement, analysis and visualization of high-throughput screen data
Source: BMC Bioinformatics. 2010 Jun 28;11:353. doi: 10.1186/1471-2105-11-353 (PMC2909220; doi:10.1186/1471-2105-11-353)
Supplement: Additional File 5 — Source code for CM, DR and SV Engines (Dittmar et al, additional file 5.zip). This file include two zip files: • ScreenMill - CM Engine.zip: contains two copies the CM Engine. One for ImageJ version 1.42, one for version 1.43+ (.txt files). • ScreenMill - DR and SV Engines.zip: contains the HTML, CSS, JavaScript, Image, and CGI (Perl) files needed to run DR and SV Engines. [file 1471-2105-11-353-S5.ZIP › Dittmar et al, addtional file 5/ScreenMill DR and SV Engines.zip!/ScreenMill DR and SV Engines/SV Engine.html]

ScreenMill - Screen Visualization Engine - Page 1

# *ScreenMill - Screen Visualization Engine*

*A tool to visually compare data.*

***Yellow border = active***

***Blue = user selected***

***Red = statistically significant***

***Green = excluded***

***Orange = dead on both***
